# Supplementary material for: Epithelial NOTCH Signaling Rewires the Tumor Microenvironment of Colorectal Cancer to Drive Poor-Prognosis Subtypes and Metastasis
Source: Cancer Cell. 2019 Sep 16;36(3):319–336.e7. doi: 10.1016/j.ccell.2019.08.003 (PMC6853173; doi:10.1016/j.ccell.2019.08.003)
Supplement: Document S1. Figures S1–S8 [file mmc1.pdf]

Cancer Cell, Volume *36*

Supplemental Information

Epithelial NOTCH Signaling Rewires the Tumor Microenvironment of Colorectal Cancer to Drive Poor-Prognosis Subtypes and Metastasis

Rene Jackstadt, Sander R. van Hooff, Joshua D. Leach, Xabier Cortes-Lavaud, Jeroen O. Lohuis, Rachel A. Ridgway, Valérie M. Wouters, Jatin Roper, Timothy J. Kendall, Campbell S. Roxburgh, Paul G. Horgan, Colin Nixon, Craig Nourse, Matthias Gunzer, William Clark, Ann Hedley, Omer H. Yilmaz, Mamunur Rashid, Peter Bailey, Andrew V. Biankin, Andrew D. Campbell, David J. Adams, Simon T. Barry, Colin W. Steele, Jan Paul Medema, and Owen J. Sansom

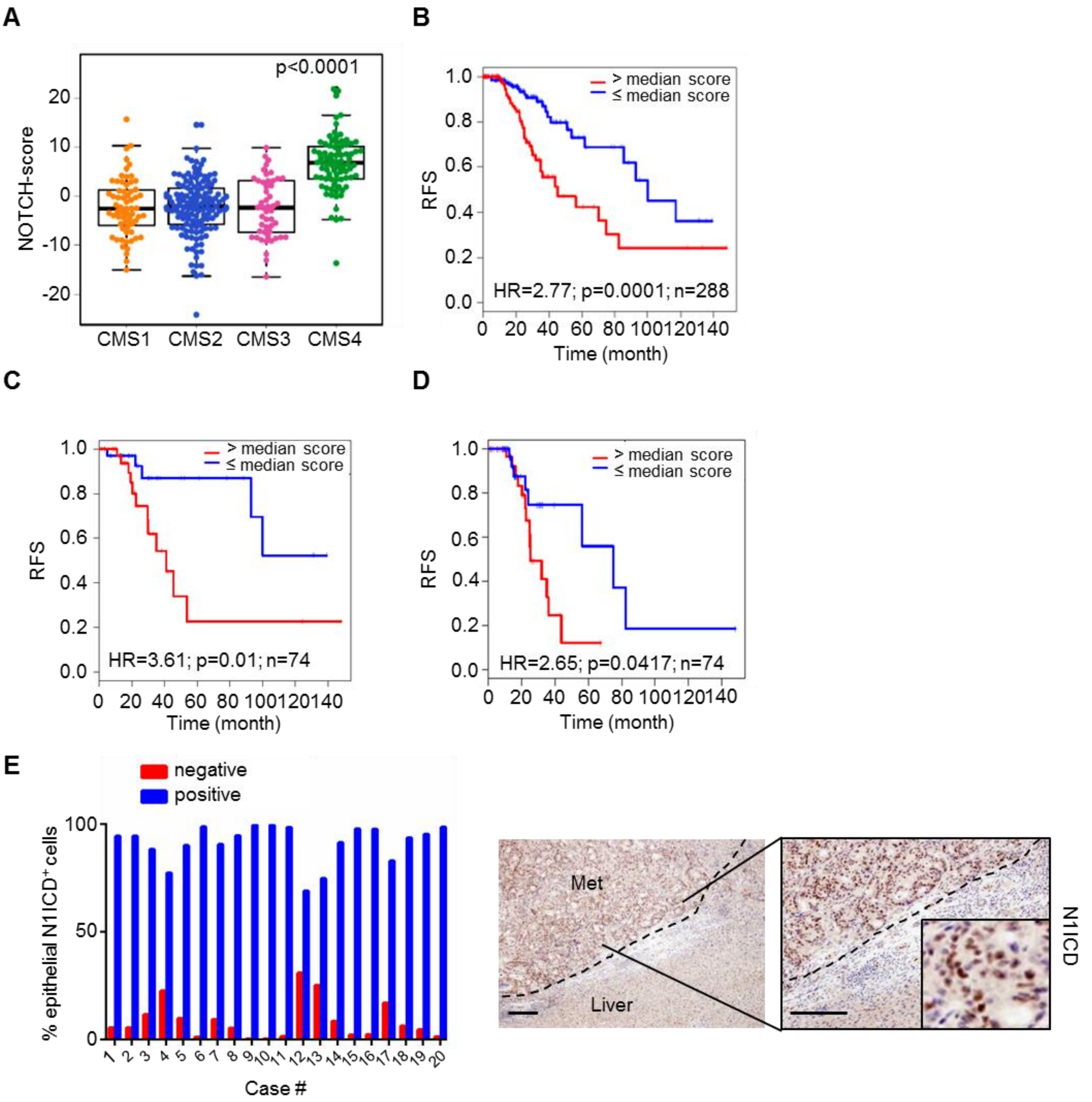

Figure S1 related to Figure 1. (A) NOTCH-score in TCGA CRC tumors separated by CMS. p value was calculated using a two sample two-tailed Welch t-test, comparing CMS4 to CMS1/2/3. The boxes indicate interquartile range (IQR), horizontal black lines median. The whiskers range to a maximum of 1.5 times the IQR. Data points outside 1.5 IQR are represented by individual dots. **(B)** Recurrence free survival (RFS) of CRC patients (TCGA), stratified using the NOTCH-score, all CMS stratified patients; blue line shows expression ≤ median score, red line shows expression > median score. **(C)** RFS of CRC patients (TCGA), stratified using the NOTCH-score, of all four CMSs in patients with *KRAS* mutations; blue line shows expression ≤ median score, red line shows expression > median score. **(D)** RFS of CRC patients (TCGA), stratified using the NOTCH-score, CMS4 stratified patients; blue line shows expression ≤ median score, red line shows expression > median score. **(E)** Left panel: Quantification of percentage positive or negative nuclear N1ICD expression (IHC) in human CRC liver metastases. Right panel: representative images of N1ICD expression (IHC) in human CRC liver metastases; scale bars, 200 µm.

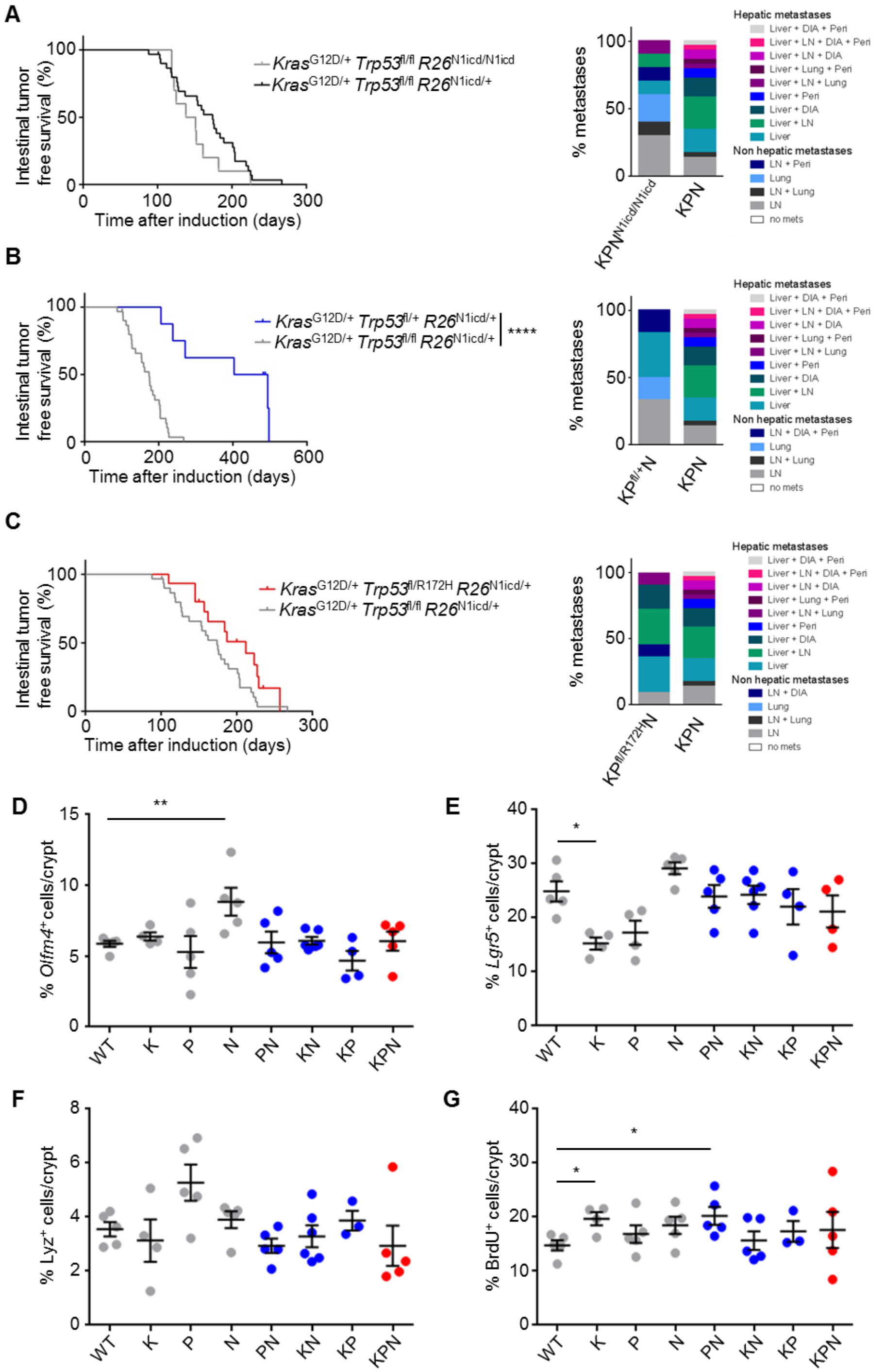

Figure S2 related to Figure 1. (A) Left panel: Kaplan-Meier survival curves of intestinal tumor free survival of KPN mice or KPN mice homozygote for *Rosa26*^{N1icd}. Right panel: Percent incidence of macroscopic metastases per genotype of *villin*Cre^{ER} *Kras*$^{G12D/+}$ *Trp53*$^{fl/fl}$ *R26*$^{N1icd/N1icd}$ (KPN$^{N1icd/N1icd}$) n=10, *villin*Cre^{ER} *Kras*$^{G12D/+}$ *Trp53*$^{fl/fl}$ *R26*$^{N1icd/+}$ (KPN) n=29 mice. **(B)** Left panel: Kaplan-Meier survival curves of intestinal tumor free survival of KPN mice or KPN mice heterozygote for floxed *Trp53*, analyzed by log-rank (Mantel–Cox) test. Right panel: Percent incidence of macroscopic metastases per genotype of *villin*Cre^{ER} *Kras*$^{G12D/+}$ *Trp53*$^{fl/+}$ *R26*$^{N1icd/+}$ (KP$^{fl/+}$N) n=6, *villin*Cre^{ER} *Kras*$^{G12D/+}$ *Trp53*$^{fl/fl}$ *R26*$^{N1icd/+}$ (KPN) n=29 mice. **(C)** Left panel: Kaplan-Meier survival curves of intestinal tumor free survival of KPN mice or KPN mice with *Trp53*$^{fl/R172H}$. Right panel: Percent incidence of macroscopic metastases per genotype of *villin*Cre^{ER} *Kras*$^{G12D/+}$ *Trp53*$^{fl/R172H}$ *R26*$^{N1icd/+}$ (KP$^{fl/R172H}$N) n=12, *villin*Cre^{ER} *Kras*$^{G12D/+}$ *Trp53*$^{fl/fl}$ *R26*$^{N1icd/+}$ (KPN) n=29 mice. In (A-C) DIA = diaphragm, LN = Lymph-node, Peri = peritoneal carcinomatosis. Quantification of percentage of positive cells per crypt stained for **(D)** *Olfm4*, **(E)** *Lgr5*, **(F)** Lysozyme (Lyz) and **(G)** BrdU. WT = *villin*Cre^{ER}; K = *villin*Cre^{ER} *Kras*$^{G12D/+}$; P = *villin*Cre^{ER} *Trp53*$^{fl/fl}$; N = *villin*Cre^{ER} *Rosa26*$^{N1icd/+}$; PN = *villin*Cre^{ER} *Trp53*$^{fl/fl}$ *Rosa26*$^{N1icd/+}$; KN = *villin*Cre^{ER} *Kras*$^{G12D/+}$ *Rosa26*$^{N1icd/+}$; KP = *villin*Cre^{ER} *Kras*$^{G12D/+}$ *Trp53*$^{fl/fl}$; KPN = *villin*Cre^{ER} *Kras*$^{G12D/+}$ *Trp53*$^{fl/fl}$ *Rosa26*$^{N1icd/+}$. Error bars represent mean ± SEM, analyzed by Mann–Whitney U test, two-tailed.

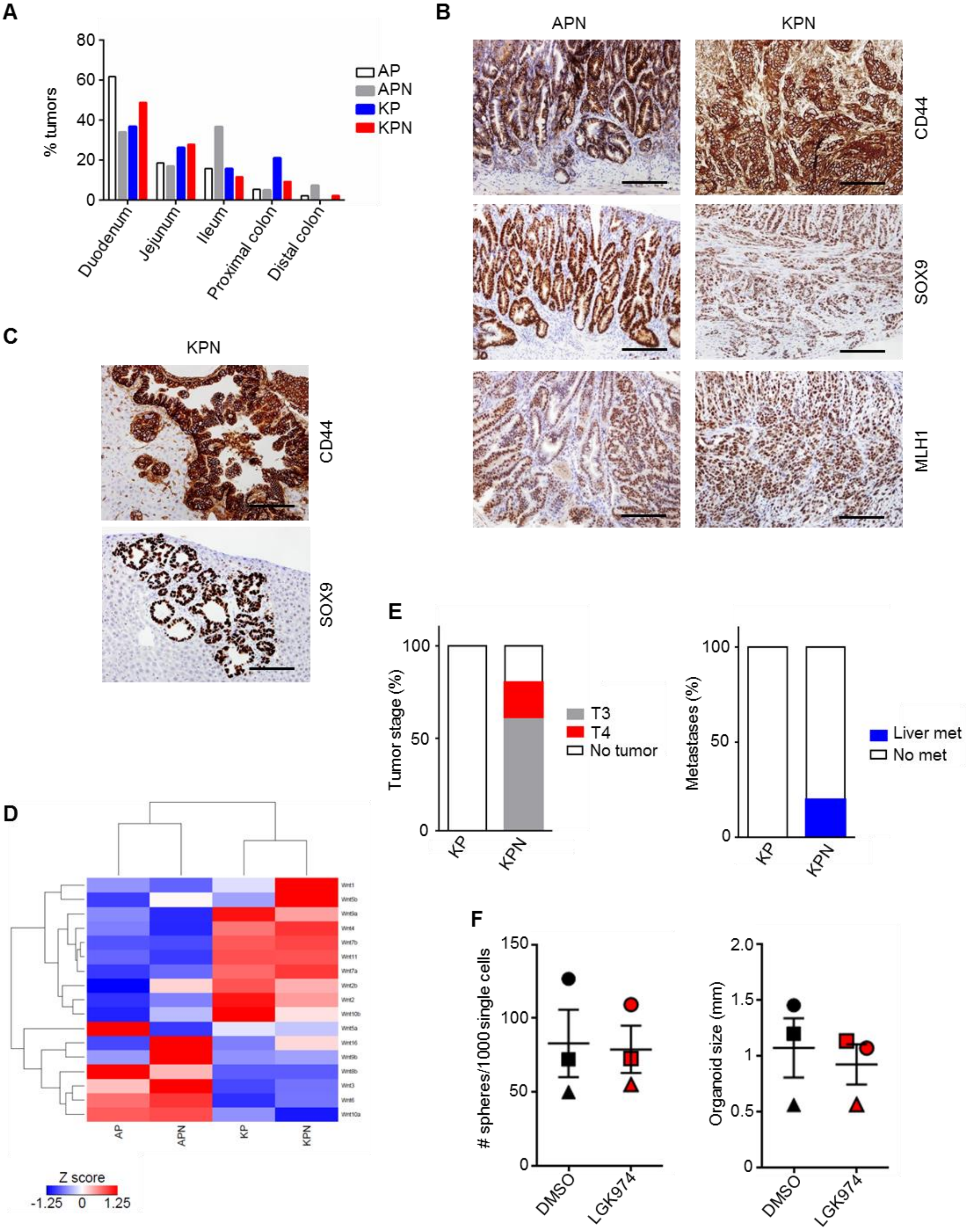

Figure S3 related to Figure 2, 3 and 4. (A) Distribution of primary tumors in the intestines of mice with indicated genotypes. AP n=10; APN n=12; KP n=14; KPN n=28. **(B)** Representative IHC for the indicated markers on primary tumors from APN and KPN mice at endpoint; scale bars, 100 µm. **(C)** Representative IHC for the indicated markers on liver metastases of KPN mice at endpoint; scale bars, 100 µm. **(D)** Heat-map showing WNT ligand expression in primary tumors of intestinal GEMMs. **(E)** Quantification of primary tumor stage and percentage of mice with metastasis at day 85 after tamoxifen administration; KP n=5; KPN n=5. **(F)** Average KP organoid size or number seven days post single cell seeding under indicated conditions; LGK974 10 µM. Each sample was generated from a separate tumor n=3; error bars represent mean ± SEM.

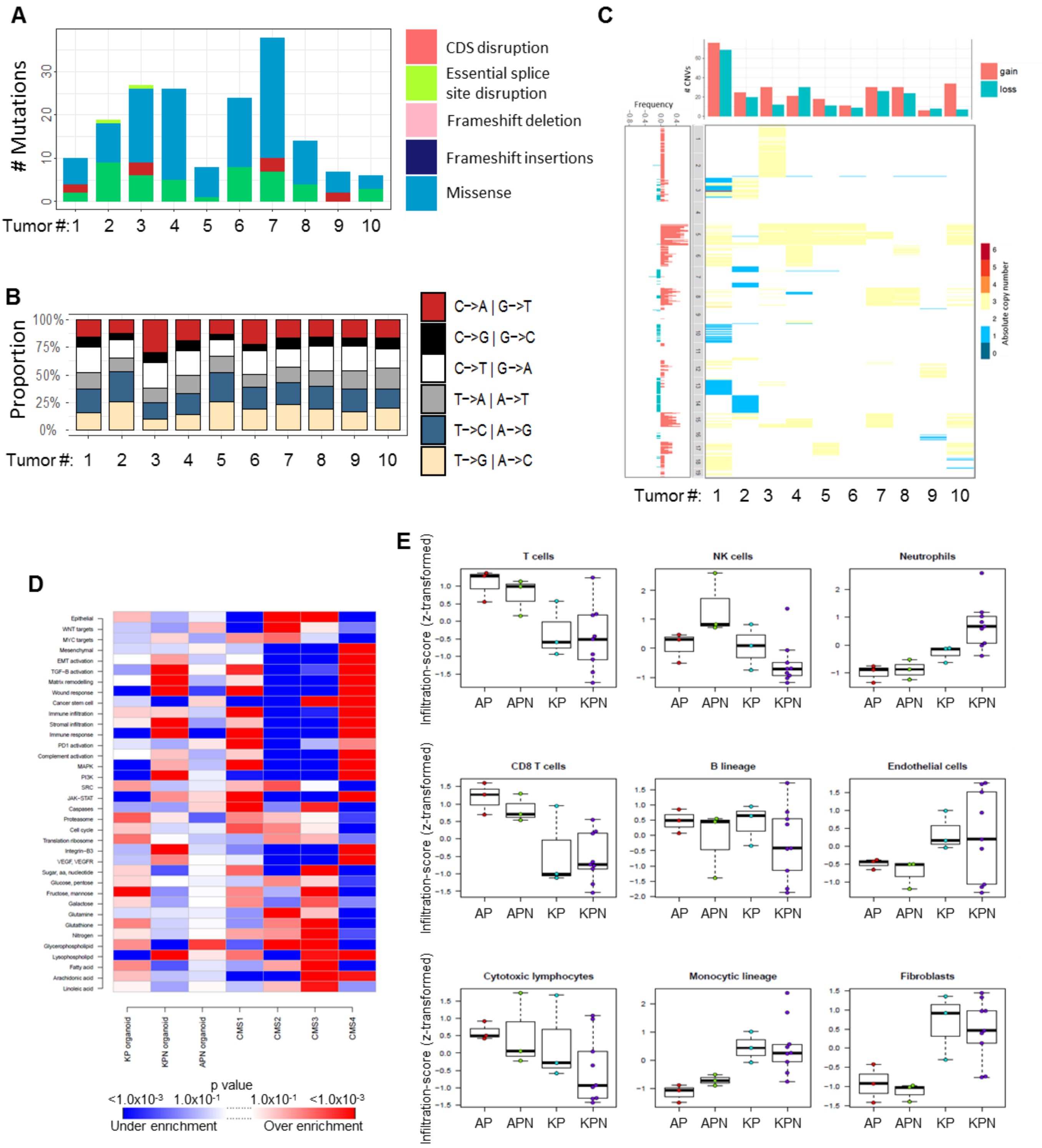

Figure S4 related to Figure 3 and 4. (A) Landscape of somatic mutations in KPN tumor derived organoids; number of somatic point mutations per sample. **(B)** Nucleotide changes per sample. **(C)** Landscape of somatic copy number changes (bottom right panel). Number of gains and losses per sample (top panel). Absolute copy number profile (bottom left panel). Gain and loss frequency across different chromosomes. **(D)** GSEA results for organoids with indicated genotypes and CMS1-4 CRC patient tumors. Replicates: APN (organoids) n=4; KP (organoids) n=3; KPN (organoids) n=3. **(E)** Box plots showing standardized infiltration scores (calculated with MCPcounter) in primary tumor derived expression profiles for indicated genotypes. The boxes indicate interquartile range (IQR), horizontal black lines median. The whiskers range to a maximum of 1.5 times the IQR. Data points outside 1.5 IQR are represented by individual dots.

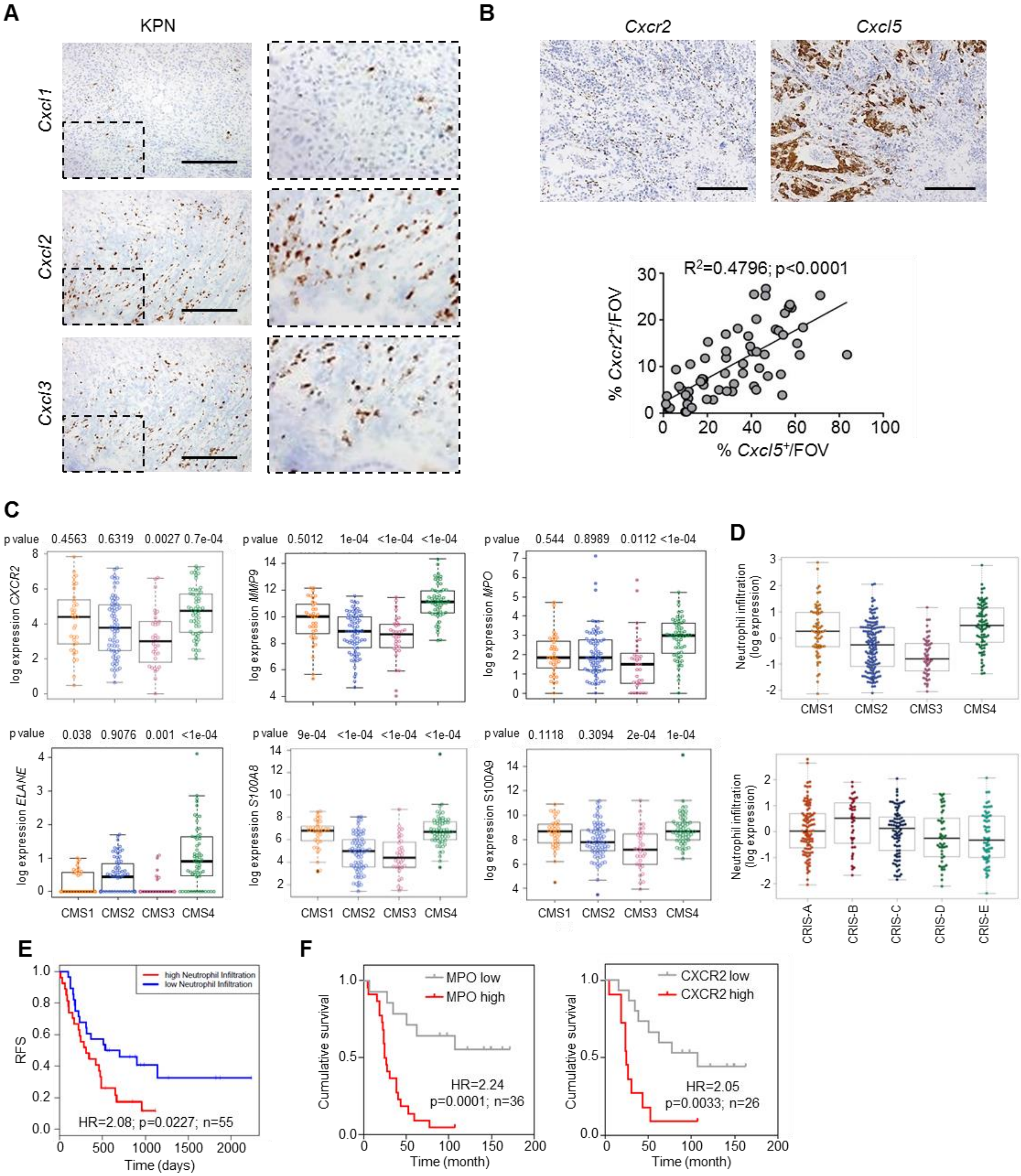

Figure S5 related to Figure 5. (A) Representative images from ISH of *Cxcl1, Cxcl2* and *Cxcl3* expression in KPN primary tumors; scale bars, 100 µm. **(B)** Representative image (top) and expression correlation analysis (bottom) of *Cxcl5* and *Cxcr2* expression (ISH) in KPN primary intestinal tumors per field of view (n=2 KPN primary tumors; 30 FOVs per tumor were analyzed); p value by Pearson correlation; scale bars, 100 µm. **(C)** Expression analysis of neutrophil markers encoding genes as indicated in CRC patient tumors (TCGA) per CMS. P values were calculated using the ANOVA-test. **(D)** Top: Neutrophil infiltration in CRC patient tumors (TCGA) per CMS; $p=3x10^{-8}$, CMS4 vs. others. Bottom: Expression analysis of Neutrophil infiltration in CRC patient tumors (TCGA) per CRIS; p=0.01, CRIS-B vs. others. P values were calculated using the ANOVA-test. **(E)** Recurrence free survival of patients with treatment naïve CRC metastases (E-TABM-1112), stratified using the neutrophil infiltration-score. Blue line shows expression ≤ median score (low), red line shows expression > median score (high). **(F)** Cumulative survival of patients (Glasgow-cohort), stratified using IHC for MPO or CXCR2 in metastases post synchronous resection of colorectal primary tumors and liver metastases. P values were calculated by log-rank (Mantel–Cox) test. The boxes in C and D indicate interquartile range (IQR), horizontal black lines median. The whiskers range to a maximum of 1.5 times the IQR. Data points outside 1.5 IQR are represented by individual dots.

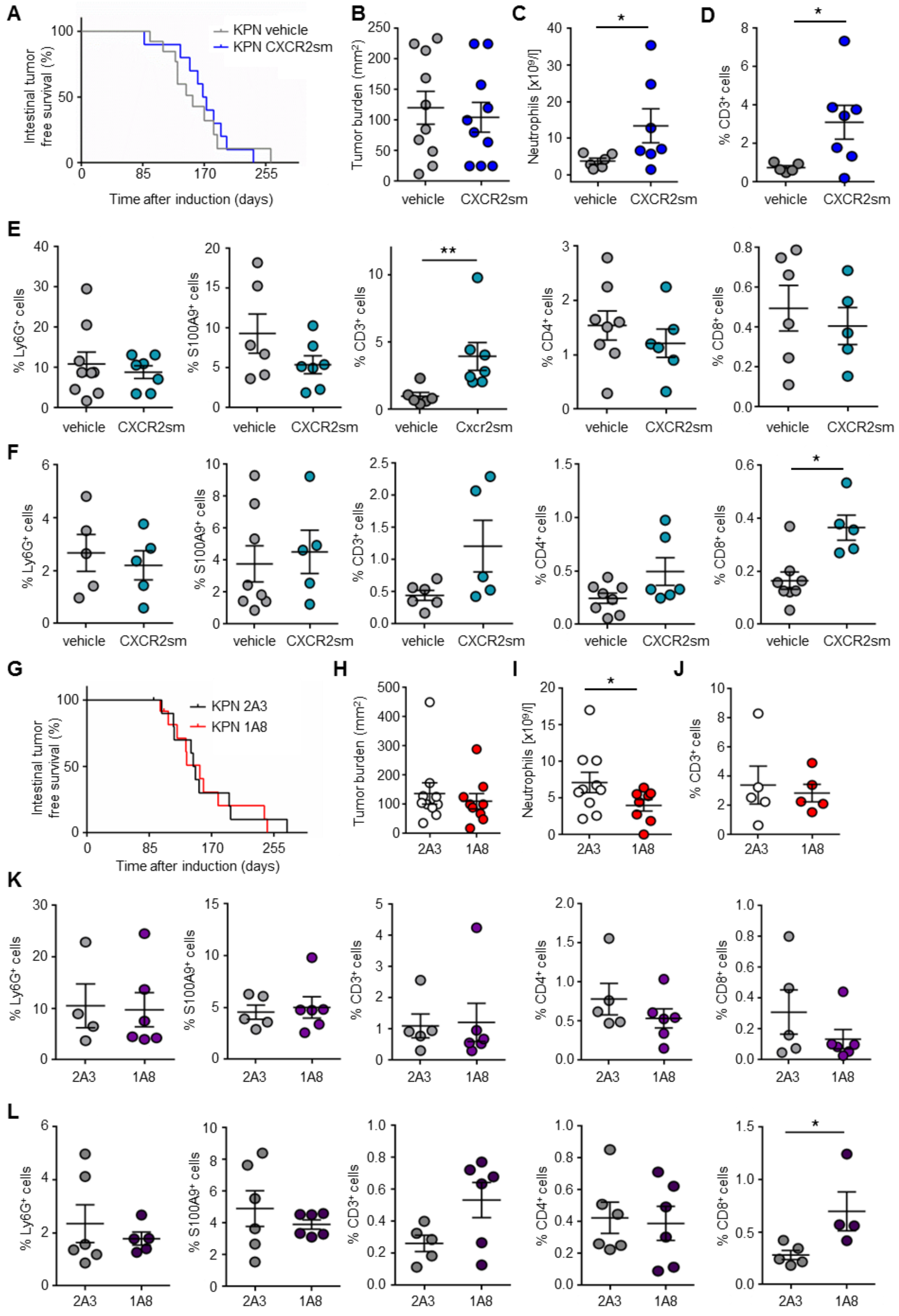

Figure S6 related to Figure 5. (A) Kaplan-Meier survival curves for intestinal tumor free survival of KPN mice treated with vehicle (n=13) or CXCR2 small molecule (CXCR2sm) (n=10), treatments started on day 85 after tamoxifen administration. **(B)** Tumor burden quantified in vehicle (n=10) and CXCR2sm (n=10) treated KPN mice at endpoint. **(C)** Blood neutrophil counts at endpoint for vehicle (n=6) and CXCR2sm (n=7) treated mice; error bars represent mean ± SEM, analyzed by Mann–Whitney U test, one-tailed. **(D)** Quantification of IHC for CD3 positive cells per KPN liver after one week treatment (n≥5). Error bars represent mean ± SEM, analyzed by Mann–Whitney U test, two-tailed. **(E)** Quantification of IHC analysis of indicated markers per primary tumor in KPN mice treated with vehicle or CXCR2sm from day 85 after tamoxifen administration (n≥5). Error bars represent mean ± SEM, analyzed by Mann–Whitney U test, two-tailed. **(F)** Quantification of IHC analysis of indicated markers per liver of KPN mice treated with vehicle or CXCR2sm from day 85 after tamoxifen administration (n≥5). Error bars represent mean ± SEM, analyzed by Mann–Whitney U test, two-tailed. **(G)** Kaplan-Meier survival curves for intestinal tumor free survival of KPN mice treated with isotype control (2A3) (n=11) or Ly6G blocking antibody (1A8) (n=12). Treatment started 85 days after tamoxifen administration. **(H)** Tumor burden of 2A3 (n=10) or 1A8 (n=9) treated KPN mice at endpoint. **(I)** Blood neutrophil count at endpoint for indicated treatments (2A3 n=10, 1A8 n=8). Error bars represent mean ± SEM., analyzed by Mann–Whitney U test, one-tailed. **(J)** Quantification of IHC for CD3 positive cells per KPN liver after one week treatment (n=5). Error bars represent mean ± SEM, analyzed by Mann–Whitney U test, two-tailed. **(K)** Quantification of IHC analysis of indicated markers per primary tumor of KPN mice treated with 2A3 or 1A8 from day 85 post tamoxifen administration (n≥4). Error bars represent mean ± SEM. **(L)** Quantification of IHC analysis of indicated markers per liver of KPN mice treated with 2A3 or 1A8 from day 85 after tamoxifen administration (n≥4). Error bars represent mean ± SEM, analyzed by Mann–Whitney U test, two-tailed.

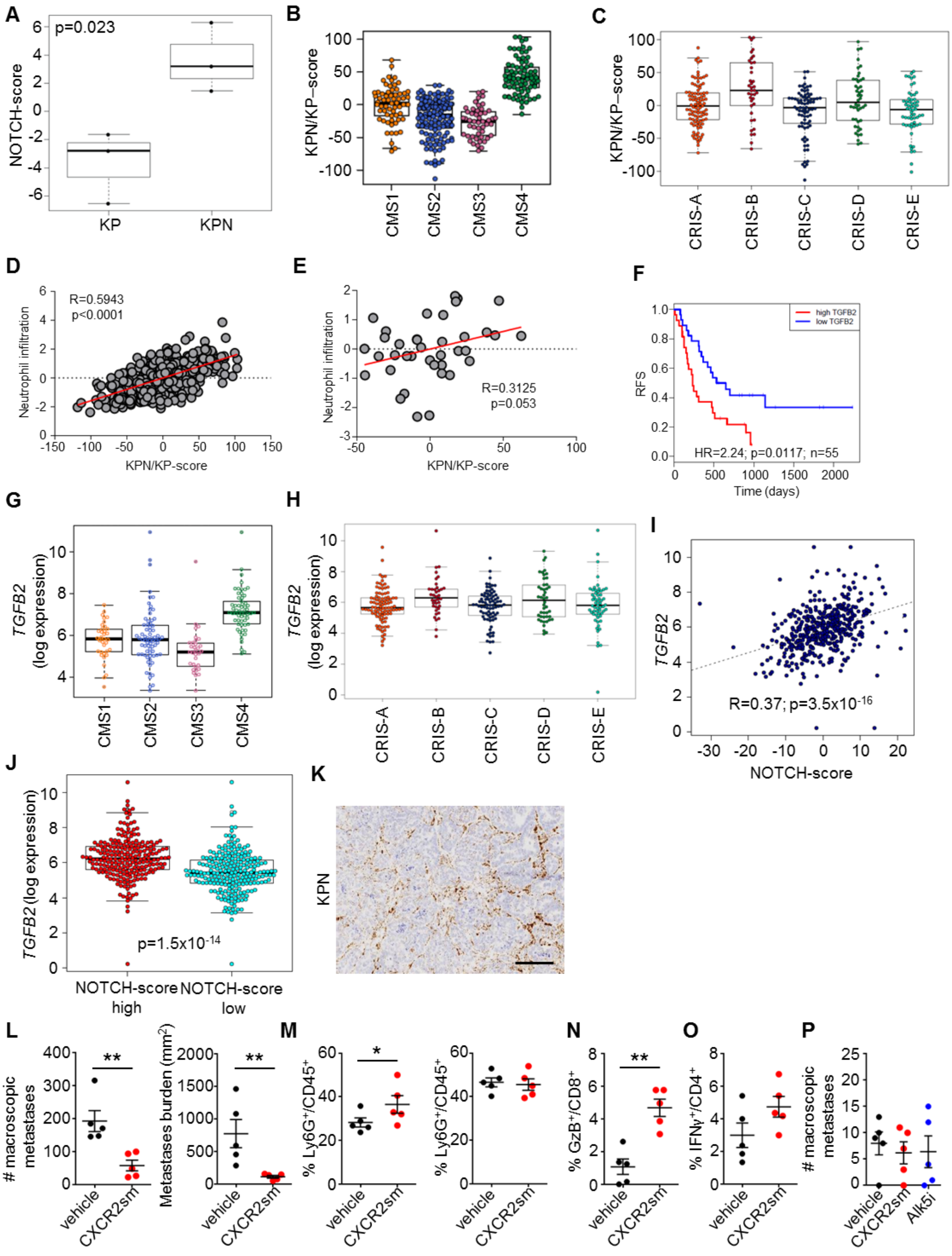

Figure S7 related to Figure 6 and 7. (A) NOTCH-score expression in KP (n=3) and KPN (n=3) organoids. P value was calculated with a t-test. **(B)** KPN/KP-score in CRC patient tumors (TCGA) per CMS. P value was calculated using the ANOVA-test; $p=5.31x10^{-33}$, CMS4 vs. others. **(C)** Expression analysis of KPN/KP-score in CRC patient tumors (TCGA) per CRIS. P value was calculated using the ANOVA-test; p=0.00027, CRIS-B vs. others. **(D)** Correlation of the KPN/KP-score and neutrophil infiltration in human CRC patient tumors (TCGA). P value was calculated by Pearson correlation. **(E)** Correlation of the KPN/KP-score and neutrophil infiltration in human serrated adenoma. P value was calculated by Pearson correlation. **(F)** Recurrence free survival of patients with treatment naïve CRC metastases (E-TABM-1112), stratified using *TGFB2* expression. Blue line shows expression ≤ median score (low), red line shows expression > median score (high). **(G)** Expression analysis of *TGFB2* in CRC patient tumors (TCGA) per CMS. P value was calculated using the ANOVA-test; $p=1x10^{-4}$, CMS4 vs. others. **(H)** Expression analysis of *TGFB2* in CRC patient tumors (TCGA) per CRIS. P value was calculated using the ANOVA-test; p=0.01, CRIS-B vs. others. **(I)** Correlation of *TGFB2* expression and the NOTCH-score in CRC patient tumors (TCGA). P value was calculated by Pearson correlation. **(J)** Expression analysis of *TGFB2* in CRC patient tumors (TCGA) with high or low NOTCH-score. P value was calculated with a t-test. **(K)** Representative ISH analysis of *Tgfb1* expression in KPN primary tumor; scale bar, 100 µm. **(L)** Number and burden of macroscopic liver metastases four weeks post intra splenic transplantation of KPN organoids (vehicle n=5, CXCR2sm n=5). **(M)** Quantification of flow cytometry analysis for neutrophils in blood (left) or liver metastases (right) four weeks post transplantation (vehicle n=5, CXCR2sm n=5), error bars represent mean ± SEM, analyzed by Mann–Whitney U test, one-tailed **(N)** Quantification of flow cytometry analysis for T cell subsets (% Granzyme B $(GzB)^{+}/CD8^{+}$) in liver metastases four weeks post transplantation (vehicle n=5, CXCR2sm n=5). **(O)** Quantification of flow cytometry analysis for T cell subsets (% Interferon-γ $(IFN\text{-}\gamma)^{+}/CD4^{+}$) in liver metastases four weeks post transplantation (vehicle n=5, CXCR2sm n=5). **(P)** Number of macroscopic liver metastases four weeks post intra splenic transplantation of KPN organoids in immune-deficient nude mice (vehicle n=5, CXCR2sm n=5, Alk5i n=5). In S7L and S7N error bars represent mean ± SEM, analyzed by Mann–Whitney U test, two-tailed. The boxes in A, B, C, G, H and J indicate interquartile range (IQR), horizontal black lines median. The whiskers range to a maximum of 1.5 times the IQR. Data points outside 1.5 IQR are represented by individual dots.

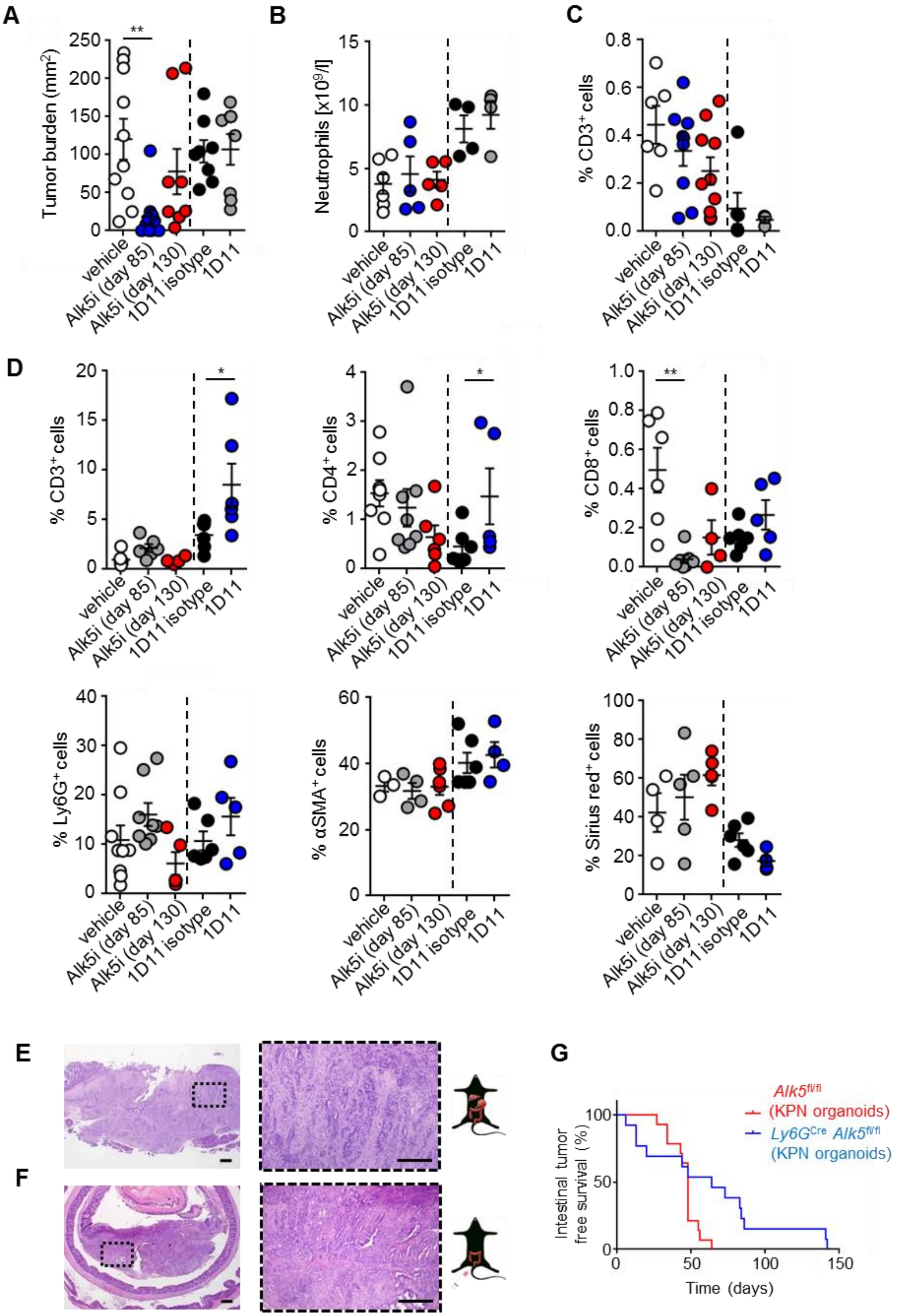

Figure S8 related to Figure 8. (A) Tumor burden of KPN mice at end point with indicated treatments (vehicle from day 85, Alk5i from day 85 or day 130, 1D11 or 1D11 isotype control from day 85 after tamoxifen administration), error bars represent mean ± SEM, analyzed by Mann–Whitney U test, two-tailed. **(B)** Blood neutrophil counts at endpoint of KPN mice treated as indicated in A; error bars represent mean ± SEM. **(C)** Quantification of IHC for CD3 positive cells per KPN liver at endpoint (n≥3), treated as indicated in A. Error bars represent mean ± SEM. **(D)** Quantification of IHC for Ly6G, CD3, CD4, CD8, αSMA and Sirius red positive cells per KPN primary tumor at endpoint after treatment as indicated in A; error bars represent mean ± SEM, analyzed by Mann–Whitney U test, two-tailed. **(E)** Representative H&E images of a KPN GEMM colonic primary tumor at end point; scale bars, 100 µm. **(F)** Representative H&E images of intra colonic transplanted KPN organoids at end point; scale bars, 100 µm. **(G)** Kaplan-Meier survival curves of intestinal tumor free survival from mice with indicated genotypes (*Alk5*$^{fl/fl}$ n=14; *Ly6G*^{Cre} *Alk5*$^{fl/fl}$ n=13) and KPN organoid transplantation.
