# Supplementary material for: Epithelial NOTCH Signaling Rewires the Tumor Microenvironment of Colorectal Cancer to Drive Poor-Prognosis Subtypes and Metastasis
Source: Cancer Cell. 2019 Sep 16;36(3):319–336.e7. doi: 10.1016/j.ccell.2019.08.003 (PMC6853173; doi:10.1016/j.ccell.2019.08.003)
Supplement: Document S2. Article plus Supplemental Information [file mmc10.pdf]

Cancer Cell

Article

Epithelial NOTCH Signaling Rewires the Tumor Microenvironment of Colorectal Cancer to Drive Poor-Prognosis Subtypes and Metastasis

Graphical Abstract

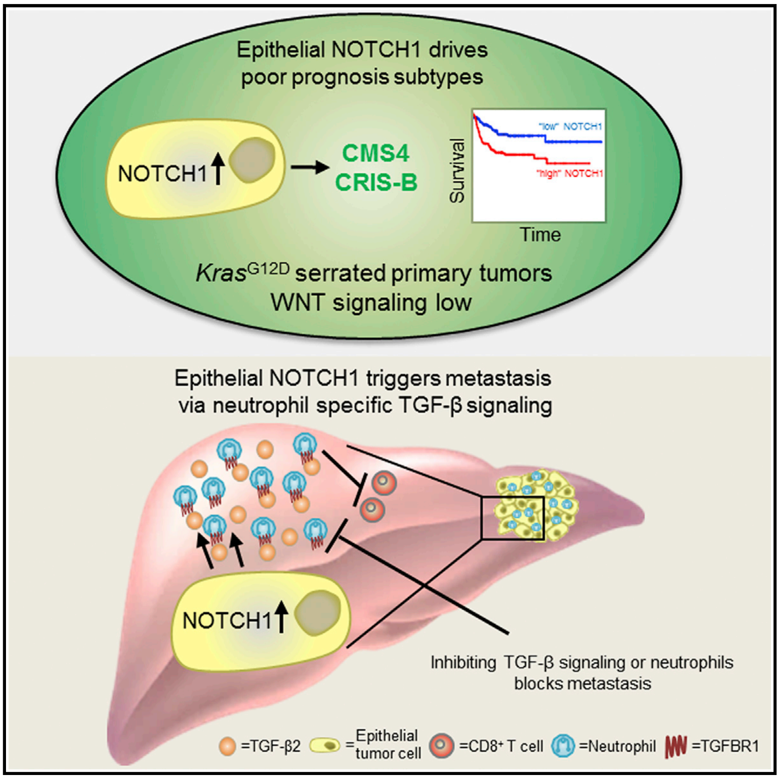

Highlights

- Epithelial NOTCH1 signaling drives metastasis in serrated CRC
- Poor-prognosis CRC subtypes CMS4/CRIS-B are controlled by NOTCH1
- TGF-β-mediated neutrophil infiltration is critical for NOTCH1-driven metastasis
- Neutrophil targeting provides therapeutic opportunity in metastatic CRC

Authors

Rene Jackstadt, Sander R. van Hooff, Joshua D. Leach, ..., Colin W. Steele, Jan Paul Medema, Owen J. Sansom

Correspondence

o.sansom@beatson.gla.ac.uk

In Brief

In a genetically engineered mouse model, Jackstadt et al. show that NOTCH1 activation drives metastasis in $KRAS^{G12D}$-driven serrated colorectal cancer (CRC) through TGFβ-dependent neutrophil recruitment. Thus, targeting neutrophil recruitment is a potential therapeutic approach in metastatic CRC.

Jackstadt et al., 2019, Cancer Cell *36*, 319–336
September 16, 2019 © 2019 The Authors. Published by Elsevier Inc.
https://doi.org/10.1016/j.ccell.2019.08.003

CellPress

CellPress

Epithelial NOTCH Signaling Rewires the Tumor Microenvironment of Colorectal Cancer to Drive Poor-Prognosis Subtypes and Metastasis

Rene Jackstadt,[1] Sander R. van Hooff,[2,3] Joshua D. Leach,[1,4] Xabier Cortes-Lavaud,[1] Jeroen O. Lohuis,[1] Rachel A. Ridgway,[1] Valérie M. Wouters,[2,3] Jatin Roper,[5] Timothy J. Kendall,[6] Campbell S. Roxburgh,[7] Paul G. Horgan,[7] Colin Nixon,[1] Craig Nourse,[1] Matthias Gunzer,[8] William Clark,[1] Ann Hedley,[1] Omer H. Yilmaz,[9,10] Mamunur Rashid,[11] Peter Bailey,[4] Andrew V. Biankin,[4] Andrew D. Campbell,[1] David J. Adams,[11] Simon T. Barry,[12] Colin W. Steele,[1,4] Jan Paul Medema,[2,3] and Owen J. Sansom[1,4,13,*]
[1]Cancer Research UK Beatson Institute, Glasgow, UK
[2]Laboratory for Experimental Oncology and Radiobiology (LEXOR), Center for Experimental Molecular Medicine (CEMM), Academic Medical Center (AMC), University of Amsterdam, Amsterdam, the Netherlands
[3]Oncode Institute, Amsterdam, the Netherlands
[4]Institute of Cancer Sciences, University of Glasgow, Garscube Estate, Glasgow, UK
[5]Department of Medicine, Division of Gastroenterology, Duke University, Durham, NC, USA
[6]Division of Pathology/Centre for Inflammation Research, University of Edinburgh, UK
[7]Academic Unit of Surgery, School of Medicine, University of Glasgow, Glasgow, UK
[8]Institute for Experimental Immunology and Imaging, University Hospital, University Duisburg-Essen, Essen, Germany
[9]Division of Gastroenterology, Tufts Medical Center, Boston, MA, USA
[10]Department of Pathology, Massachusetts General Hospital, Boston, MA, USA
[11]Wellcome Sanger Institute, Wellcome Genome Campus, Hinxton, Cambridge, UK
[12]Bioscience, Oncology R&D, AstraZeneca, Cambridge, UK
[13]Lead Contact
*Correspondence: o.sansom@beatson.gla.ac.uk
https://doi.org/10.1016/j.ccell.2019.08.003

SUMMARY

The metastatic process of colorectal cancer (CRC) is not fully understood and effective therapies are lacking. We show that activation of NOTCH1 signaling in the murine intestinal epithelium leads to highly penetrant metastasis (100% metastasis; with >80% liver metastases) in *Kras*^{G12D}-driven serrated cancer. Transcriptional profiling reveals that epithelial NOTCH1 signaling creates a tumor microenvironment (TME) reminiscent of poorly prognostic human CRC subtypes (CMS4 and CRIS-B), and drives metastasis through transforming growth factor (TGF) β-dependent neutrophil recruitment. Importantly, inhibition of this recruitment with clinically relevant therapeutic agents blocks metastasis. We propose that NOTCH1 signaling is key to CRC progression and should be exploited clinically.

INTRODUCTION

Patient mortality in CRC is closely associated with metastasis (Jemal et al., 2011), with an overall 5-year survival rate for late-stage patients of 5%–10%. Resection of both primary and metastatic lesions provides the best prognosis for these patients, but post-intervention recurrence is very common due to disseminated latent or therapy-resistant tumor cells (Tauriello et al., 2017). Consequently, preclinical models which faithfully recapitulate the processes of CRC metastasis are required.

Significance

CRC is the second most common cause of cancer death, with metastasis the key contributor to CRC-associated death. Thus, a pressing need exists for therapeutic strategies which target disease subtypes with the poorest prognosis. We have generated a genetically engineered mouse model (GEMM) of metastatic CRC, which represents a fundamental advance in preclinical modeling. In doing so, we identify epithelial NOTCH1 signaling as a critical feature of both the poorest prognosis subtypes and of metastatic seeding at the secondary site. Crucially, targeting NOTCH1-driven neutrophil recruitment and TGF-β signaling with clinically relevant small-molecule inhibitors or therapeutic antibodies has a profound impact on metastatic burden *in vivo*.

 © 2019 The Authors. Published by Elsevier Inc.
This is an open access article under the CC BY license (http://creativecommons.org/licenses/by/4.0/).

Stratification of CRC by transcriptional profiling (Guinney et al., 2015) has allowed classification of CRC into four consensus molecular subtypes (CMSs). CMS1 tumors are likely to be microsatellite unstable, hyper-mutated, and characterized by lymphocytic infiltration. CMS2 and CMS3 tumors exhibit high levels of WNT signaling, little immune infiltration and intermediate overall and relapse-free survival. Patients with ''mesenchymal'' CMS4 tumors have the worst overall and relapse-free survival rate and these tumors are characterized by significant fibroblast and innate immune cell infiltration, and elevated TGF-β signaling (Dienstmann et al., 2017; Becht et al., 2016a). As these transcriptional signatures are generated from whole tumors, the presence of stromal cells contributes significantly (Calon et al., 2015; Isella et al., 2015; McCorry et al., 2018), confounding analysis. While the contribution of epithelial cells to stromal infiltration/adaptation is not fully understood (Wellenstein and de Visser, 2018), cell-intrinsic transcriptional signatures (CRIS) have been shown to have prognostic implications (Dunne et al., 2017; Isella et al., 2017). In particular, the CRIS-B signature predicts poor prognosis and is enriched for signatures associated with epithelial-mesenchymal transition (EMT) and TGF-β signaling. Notably, while CMS4 and CRIS-B are characterized by the same activated programs, the composition of gene signatures is different and CRIS-B is a composition of mainly CMS1 and CMS4 genes. Crucially, mutational data does not stratify the different CRC subtypes and the mechanisms which drive subtypes are not known (Guinney et al., 2015).

There are two postulated routes by which metastatic CRC (mCRC) arises. The classical route is initiated by mutations in the *APC* tumor suppressor gene which is followed by alterations in mitogen-activated protein kinase (MAPK), *TP53*, and TGF-β signaling during progression (Fearon and Vogelstein, 1990). These tumors develop from adenomas with tubular morphology (Fearon, 2011). Recent efforts to model metastatic disease with compounding mutations in the intestine of APC-deficient mice yielded tumors that readily progressed to adenocarcinoma but showed limited metastasis (Sakai et al., 2018). Intriguingly, if APC-deficient tumors with compounding mutations are propagated *ex vivo* as organoids and re-implanted into mice, metastasis occurs (Tauriello et al., 2018; de Sousa e Melo et al., 2017; O'Rourke et al., 2017). Alternatively, CRC progression can be initiated by *KRAS* or *BRAF* mutations, with tumor development from adenomas with a serrated morphology (Jass et al., 2002). Importantly, patients with serrated adenoma-associated signatures have a poorer prognosis than those with ''classical'' tubular adenomas (De Sousa et al., 2013). These adenomas may progress to high-grade carcinoma through p16/*CDKN2A* promoter hyper-methylation and subsequent gene silencing, or via mutation of *TP53* (IJspeert et al., 2015). *Braf*-mutant genetically engineered mouse models (GEMMs) of CRC exhibit activated WNT signaling, indicated by nuclear accumulation of β-catenin, while *KRAS*-mutant tumors appear to develop independently of WNT pathway activation (Bennecke et al., 2010; Janssen et al., 2002; Trobridge et al., 2009). Nevertheless, these GEMMs develop few distant metastases and have a long latency.

Despite some caveats, GEMMs are powerful tools to study tumor biology in an autochthonous setting, and are the gold standard in preclinical CRC research. The major weakness of current CRC GEMMs is the lack of a complete adenoma-carcinoma-metastasis cascade and the absence of highly penetrant metastases, particularly to distant organs such as the liver (Jackstadt and Sansom, 2016; Romano et al., 2018). For this reason, current models can be seen as excellent tools to study early-stage disease rather than malignant progression, with transplantation of tumor-derived organoids currently being the best alternative for analysis of metastatic spread (Romano et al., 2018; Tauriello et al., 2018). Transplantation models have highlighted a key role for $LGR5^+$ stem cells in metastasis (de Sousa e Melo et al., 2017) and have suggested that TGF-β inhibitors may have efficacy in *Apc*-mutation-driven mCRC (Tauriello et al., 2018).

Activated NOTCH1 signaling has been observed in CRC and other cancer types (Sancho et al., 2015; Noah and Shroyer, 2013; Irshad et al., 2017). This activation can occur via NOTCH1 ligands on the surface of tumor cells or by components of the TME such as endothelial or innate immune cells (Meurette and Mehlen, 2018). Tumor cell-autonomous signaling can also occur by *NOTCH1* receptor copy-number gain, reported in 22% of CRCs, with negative prognostic value (Arcaroli et al., 2016). In addition NOTCH1 signaling can be activated via mutation of *FBXW7*, found in 11% of human CRCs (Cancer Genome Atlas Network, 2012; Babaei-Jadidi et al., 2011). Activation of NOTCH1 signaling can contribute to cancer cell stemness, invasion, and metastasis (Lu et al., 2013; Sonoshita et al., 2011, 2015; Rodilla et al., 2009; Wieland et al., 2017). Moreover, recent combination of activated NOTCH1 signaling and *Trp53* deletion in the intestine resulted in metastatic disease, albeit with long latency and relatively low penetrance (10% liver metastases) (Chanrion et al., 2014), limiting preclinical relevance. Importantly, the molecular mechanism driving NOTCH1-dependent metastasis and the requirement for additional oncogenic driver mutations remains unclear.

There is an urgent need for improved therapeutic options for patients with advanced mCRC. Currently, molecular subtyping is the most effective strategy to identify patients with the poorest prognosis. For this reason, subtype-specific preclinical models are vital for development of new therapeutic approaches.

RESULTS

Mutation Context-Dependent Ability of NOTCH1 to Drive Intestinal Cancer Metastasis

Given associations between NOTCH signaling and CRC we generated a NOTCH-score (Kwon et al., 2016), based on expression of pathway components, and applied this to The Cancer Genome Atlas (TCGA) human CRC dataset (Cancer Genome Atlas Network, 2012). We found that a high NOTCH-score is significantly associated with CMS4 and poor prognosis (Figures S1A and S1B). Interestingly, when further stratified, the NOTCH-score remained prognostic when *KRAS* was mutated (Figure S1C), and segregated the poorest prognosis patients in CMS4 (Figure S1D). In addition, we found a high percentage of human CRC metastasis strongly positive for NOTCH1 intracellular domain (N1ICD), indicative of activated NOTCH1 signaling in human CRC metastasis (Figure S1E).

In light of these observations, we sought to test the functional role of NOTCH1 signaling in CRC. This was achieved using the

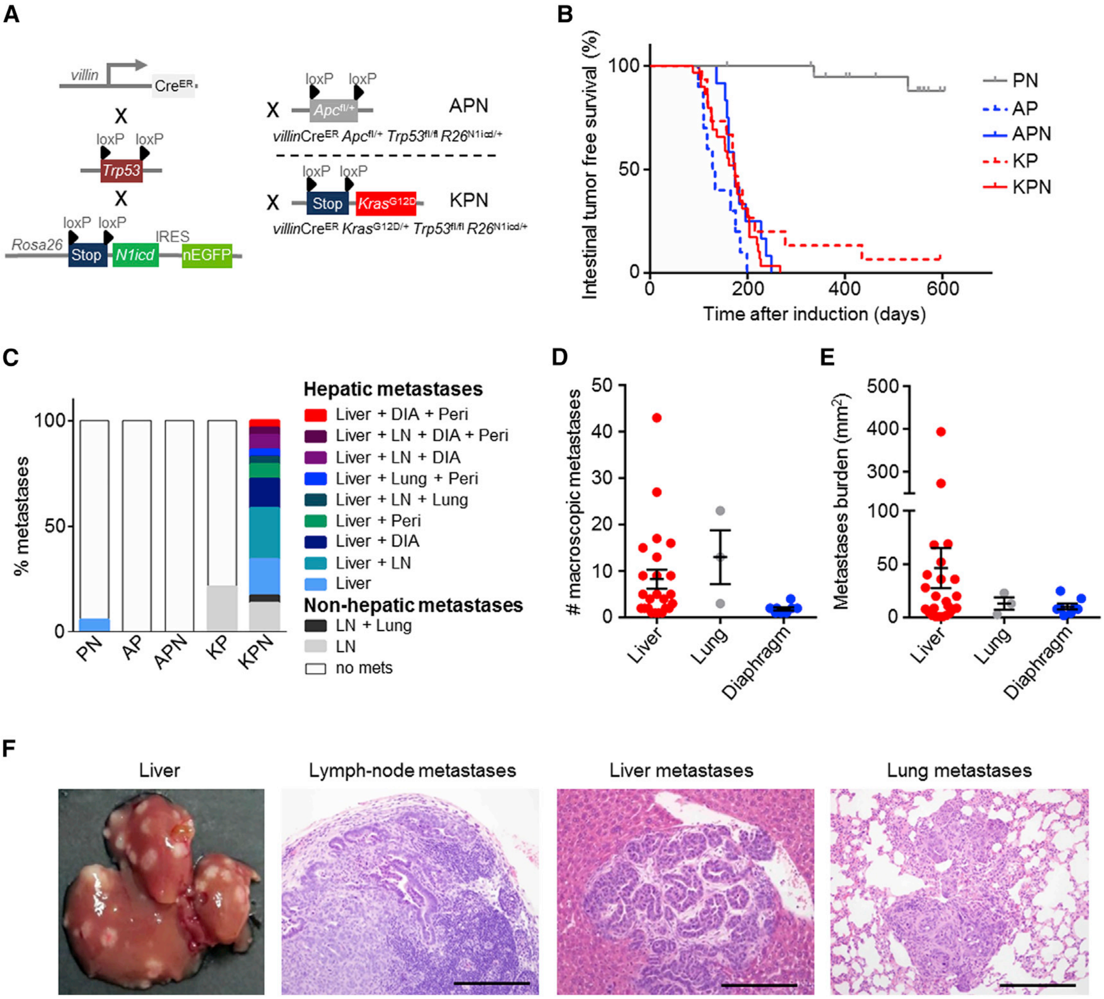

Figure 1. NOTCH1 Drives Intestinal Metastasis in an Autochthonous Model

(A) Schematic description of genetic crossing strategies. Cre, cre-recombinase; ER, estrogen receptor; *loxP*, Cre-Lox recombination site; IRES, internal ribosome entry site.

(B) Kaplan-Meier survival curves of intestinal tumor free survival; PN, n = 21; AP, n = 10; APN, n = 12; KP, n = 15; KPN, n = 31.

(C) Incidence of metastases (%) per genotype; PN, n = 21; AP, n = 10; APN, n = 12; KP, n = 14; KPN, n = 29. DIA, diaphragm; LN, lymph-node; Peri, peritoneal carcinomatosis.

(D and E) Number (D) and burden (E) of macroscopic metastases of KPN mice. Error bars represent mean ± SEM.

(F) Left image: representative image of macroscopic liver metastatic burden of KPN mice. Right images: representative H&Es of KPN metastases. Scale bars, 100 μm.

See also Figures S1 and S2.

inducible enterocyte-specific *villin*Cre^{ER} to recombine either one copy of *Apc*$^{fl/+}$ or activate *Kras*$^{G12D/+}$ in combination with deletion of *Trp53*$^{fl/fl}$, and overexpression of the transcriptionally active N1ICD (Figure 1A). Consistent with previous studies, *villin*Cre^{ER} *Trp53*$^{fl/fl}$ *Rosa26*$^{N1icd/+}$ (PN) mice (Chanrion et al., 2014) developed tumors at long latency (Figure 1B). Importantly, all induced *villin*Cre^{ER} *Kras*$^{G12D/+}$ *Trp53*$^{fl/fl}$ *Rosa26*$^{N1icd/+}$ (KPN) mice that developed intestinal adenocarcinoma exhibited metastases to lymph nodes, lungs, liver, and/or diaphragm at clinical endpoint (Figure 1C). A total of 83% (24/29) of KPN mice had liver metastases, recapitulating human disease (Figures 1D-1F). In contrast, APC-deficient models such as *villin*Cre^{ER} *Apc*$^{fl/+}$ *Trp53*$^{fl/fl}$ *Rosa26*$^{N1icd/+}$ (APN) or *villin*Cre^{ER} *Apc*$^{fl/+}$ *Trp53*$^{fl/fl}$ (AP) did not develop metastases. PN or *villin*Cre^{ER} *Kras*$^{G12D/+}$ *Trp53*$^{fl/fl}$ (KP) mice developed few metastases and very rarely to distant sites: 5% liver and ~20% lymph nodes, respectively (Figure 1C). Expression of two copies of the *N1icd* allele (Figure S2A) or one copy of mutant *Trp53*$^{fl/R172H}$ in KPN mice did not change survival and/or metastatic burden (Figures S2B and S2C). Furthermore, mutations had only a mild impact on intestinal homeostasis (Figures S2D–S2G). Given that KP and KPN mice exhibited similar latency (Figure 1B), but only KPN mice displayed significantly increased metastatic burden (Figure 1C), we concluded that epithelial NOTCH1 drives metastasis in a setting where *Trp53* is mutated and RAS/MAPK signaling is activated.

KPN Tumors Are of Serrated Origin

Human serrated CRCs have been associated with *KRAS* mutations (IJspeert et al., 2015), and these morphological features are reported to be recapitulated in the tumors of *Kras*^{G12D}-driven intestinal GEMMs (Bennecke et al., 2010). Histological analysis of KPN tumors confirmed a serrated morphology of primary tumors, while tumors driven by *Apc* deletion exhibited a tubular morphology (Figure 2A). Consistent with the metastatic spread of KPN tumors, primary tumors were highly invasive and poorly differentiated, exhibited a high collagen content, significant infiltration of cancer associated fibroblasts (CAFs) and hypoxia, all features typical of advanced disease (Figures 2B–2D). On average, KPN mice developed two tumors per intestine (Figures 2E, 2F, and S3A). We analyzed the expression of the DNA mismatch repair protein MLH1 in primary tumors of APN and KPN mice. Retained expression of MLH1 indicates that these tumors are microsatellite stable (MSS) (Figure S3B). Therefore, KPN tumors represent models of MSS serrated intestinal cancer in which NOTCH1 signaling drives metastasis without impacting tumor initiation.

Alteration of WNT Signaling in the Metastatic KPN Model

Human serrated polyps show reduced WNT pathway activity compared with tubular adenomas which harbor *APC* mutations (Figure 3A) (Borowsky et al., 2018; Bennecke et al., 2010). Comparison of WNT target gene expression between GEMM primary tumors and human serrated adenoma (Fessler et al., 2016) revealed that KPs and KPNs are closely related to serrated tumors (Figure 3B). APN tumors exhibited significant activation of canonical WNT signaling, indicated by nuclear accumulation of β-catenin, with lower activation observed in KPN tumors (Figure 3C). This was reflected by distinct patterns of WNT target gene expression in each primary tumor type (Figures 3B, 3D, and S3B). Moreover, liver metastases from KPN tumors did not have a marked accumulation of nuclear β-catenin, although elevated expression of some WNT targets, including CD44 and SOX9, was observed (Figures 3C and S3C). This recapitulates activation of WNT seen in human serrated tumors and indicates that hyper-activation of epithelial canonical WNT signaling is not essential for metastasis.

Given these moderate levels of WNT signaling and reported upregulation of WNT ligands or R-spondins in CRC (Seshagiri et al., 2012), we examined ligand deregulation or ligand dependence in our models. RNA-sequencing data exhibited profound expression of WNT ligands (Figure S3D). To test ligand functionality we treated KPN mice with LGK974, a clinically relevant PORCUPINE inhibitor, from 85 days after induction, blocking WNT ligand secretion (Liu et al., 2013) (Figures 3E and S3E). This treatment had no impact on survival or metastatic rate (Figures 3F and 3G; Table S1). To understand the mechanism of WNT ligand independence, we derived organoid cultures from KPN primary tumors. When these organoids were passaged or seeded as single cells, they grew independently of WNT agonist R-spondin1 and were refractory to LGK974 treatment (Figures 3H–3J). Similarly, KP organoids were refractory to LGK974 treatment indicating a NOTCH1-independent mechanism (Figure S3F). This suggests an epithelial cell-intrinsic mechanism of WNT ligand-independent growth, or independence from WNT signaling altogether. To identify drivers of WNT activation, we applied whole-genome sequencing to ten KPN primary tumor-derived organoid lines. This approach confirmed loss of *Trp53* and MSS status (overall 1.59 mutations/Mb; coding mutation rate 1.31 mutations/Mb) (Figures S4A–S4C and Table S2). Strikingly, four out of ten (40%) organoid lines had homozygous mutations in *Csnk1a1* (encoding casein kinase 1α), a component of the β-catenin destruction complex (Figure 3K). Intriguingly, *Csnk1a1* deletion in intestinal epithelial cells has been shown to trigger tumorigenesis only in combination with loss of *Trp53* (Elyada et al., 2011). Furthermore, two lines showed *Ephb2* missense mutations (Figure 3K), which has been associated with CRC progression (Batlle et al., 2005; Clevers and Batlle, 2006). Importantly, while these mutations drive increased expression of selected WNT targets, they appear to be weaker activators of the pathway than *Apc* loss and critically mimic levels found in human serrated tumors.

Epithelial NOTCH1 Drives Subtypes of Human CRC with Poorest Prognosis

To better understand how our model, and more broadly, NOTCH1 signaling relates to human CRC, we generated transcriptome-wide expression profiles from tumor tissue (consisting of epithelium and stroma). Comparison of signatures generated from both the serrated (KPN) and tubular (APN) tumors (Table S3) with human data revealed a poorer prognosis for patients resembling the KPN signature (Figure 4A), in line with the poor prognosis associated with human serrated CRC (De Sousa et al., 2013). Interestingly, when we analyzed organoid expression profiles derived from APN or KPN tumors (Table S4) this survival segregation still holds (Figure 4B), demonstrating the predictive value of epithelial KPN signatures. Comparison of mouse intestinal tumors with CMSs revealed a NOTCH1-dependent positive correlation between the KPN transcriptome and CMS4, and a negative correlation with CMS2/3 (Figure 4C). Strikingly, tumor models driven by APC loss correlate with CMS2/3 (Figure 4C), which confers better disease prognosis (Guinney et al., 2015). Moreover, cross-comparison with CRIS signatures revealed that KPN tumors strongly correlate with CRIS-B (Figure 4D), associated with poor prognosis (Isella et al., 2017). Gene set enrichment analysis (GSEA) indicates that KPN tumors are enriched for CMS4/CRIS-B-associated signatures such as vascular endothelial growth factor/vascular endothelial growth factor receptor, EMT, and TGF-β activation (Figures 4E and S4D; Table S5). These data demonstrate that the GEMMs described here exhibit transcriptional overlap with the subset of human CRCs with poorest prognosis and that epithelial NOTCH1 is a key driver of those subtypes.

Epithelial NOTCH1 Controls Neutrophil Recruitment to Drive Metastasis

The current paradigm suggests that stromal signatures are associated with poor prognosis, which is particularly pertinent to the "mesenchymal" CMS4 tumor signature. In light of this, we have compared our transcriptional profiles with data used to characterize human CMS4 CRC as highly enriched for myeloid, angiogenic, inflammatory, fibroblast, and immunosuppressive cell signatures (Becht et al., 2016a, 2016b). While many of these features were recapitulated in metastatic KPN tumors (Figure 5A), most were also present in the non-metastatic KP model (Figures

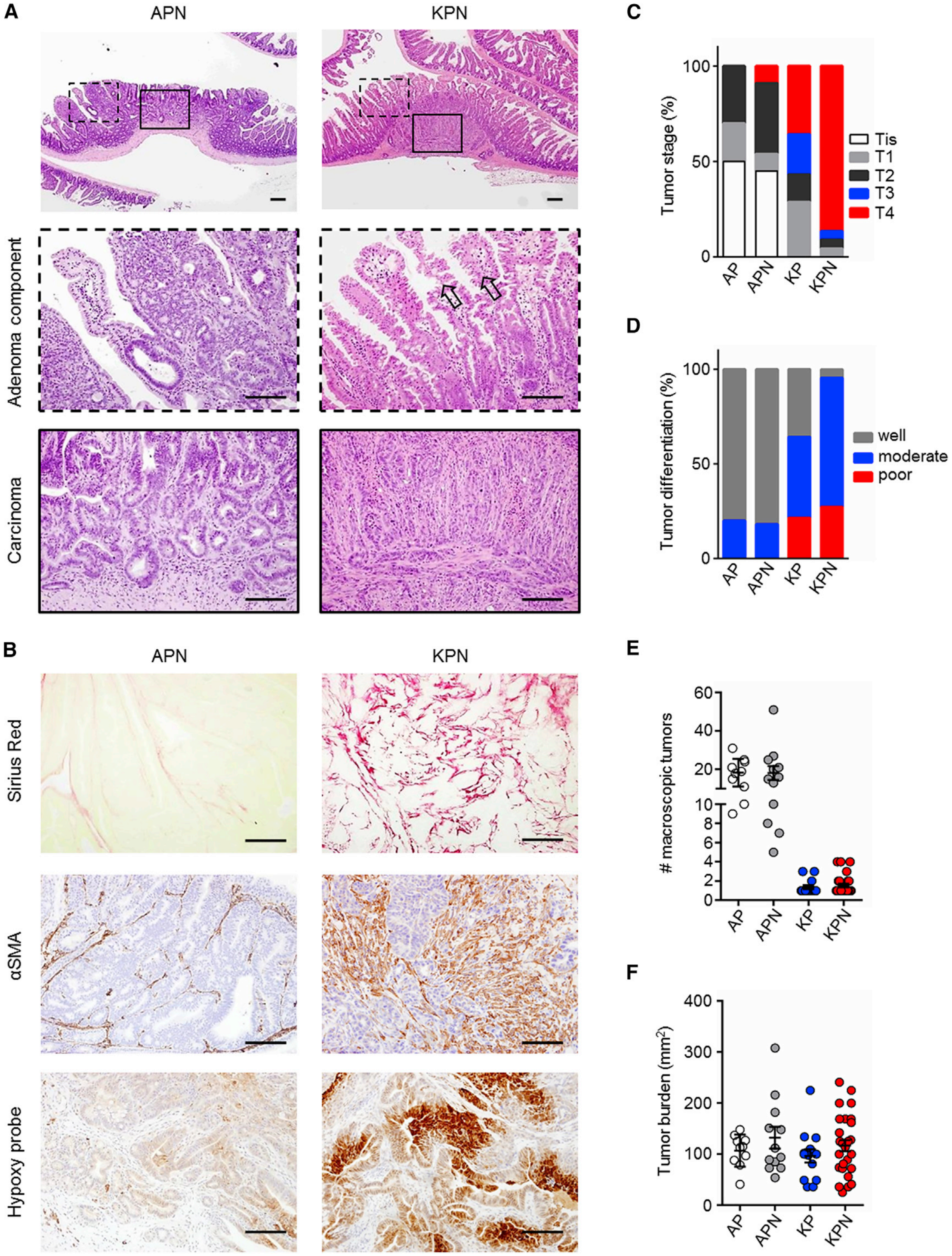

Figure 2. Morphological Analysis of Primary Tumors

(A) Representative H&E images of primary tumors. Scale bars, 100 μm. Arrows indicate serrated morphology.

(B) Representative images of indicated markers on primary tumors. Scale bars, 100 μm.

(C and D) Tumor stage (C) and differentiation at endpoint (D).

(E) Macroscopic primary tumors per mouse.

(F) Macroscopic primary tumor burden per mouse.

In (C–F): AP, n = 10; APN, n ≥ 11; KP, n = 14; KPN, n ≥ 22. Error bars in (E and F) represent mean ± SEM. See also Figure S3.

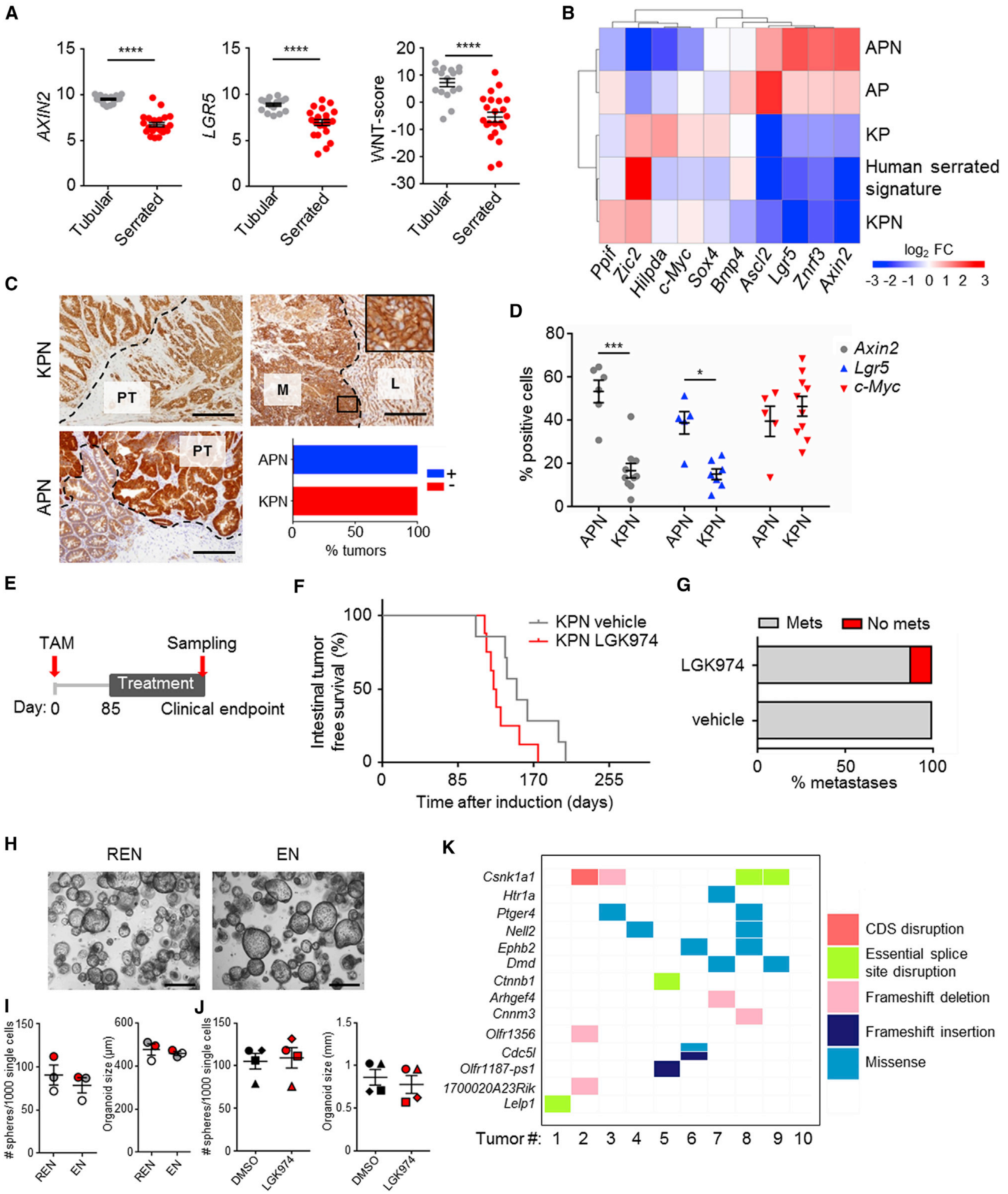

Figure 3. Role of WNT Signaling in Metastatic KPN Tumors

(A) Expression of WNT targets in human tubular or serrated adenoma.

(B) Heatmap of a human serrated signature versus mouse primary tumor signatures.

(C) β-Catenin IHC of primary tumors. M, metastases; L, liver; PT, primary tumor. Scale bars, 100 μm. Right bottom: quantification of nuclear β-catenin in primary tumors ($n \geq 10$).

(D) Quantification of *in situ* hybridization (ISH) for positive cells on primary tumors (APN, $n \geq 5$; KPN, $n \geq 7$).

(legend continued on next page)

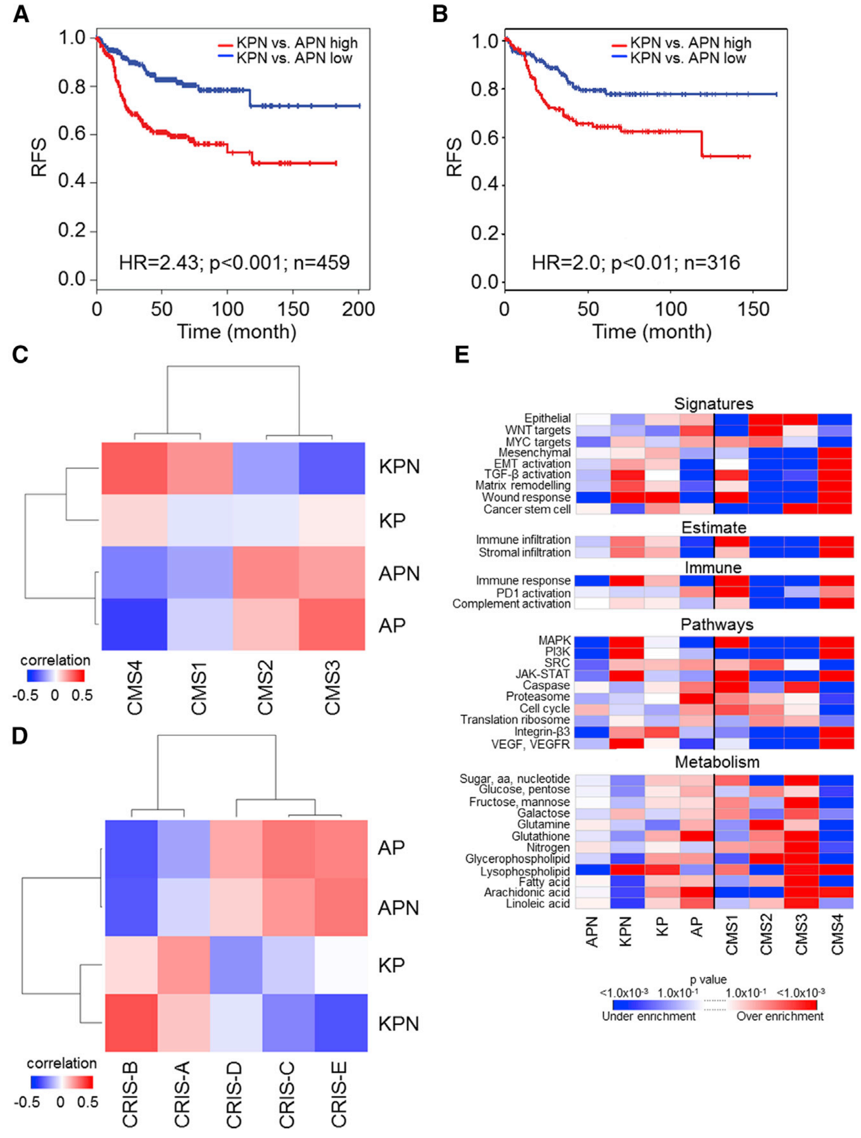

Figure 4. Cross-Comparison and Subtyping of GEMMs to Human CRC

(A) Recurrence-free survival (RFS) of CRC patients stratified using a KPN versus APN tumor signature, for patients of all four CMSs.

(B) RFS of CRC patients stratified using a KPN versus APN organoid signature, for patients of all four CMSs. In (A) and (B), the blue line shows correlation ≤ 0.1 (low), the red line shows correlation >0.1 (high).

(C) Heatmap showing expression correlation of intestinal cancer GEMMs with patient-derived CMSs. p value for CMS4-KPN versus CMS4-KP correlation ($p = 0.003$) was obtained using a Fisher r-z transformation.

(D) Heatmap showing expression correlation of intestinal cancer GEMMs with patient-derived CRISs.

(E) GSEA results for GEMMs and CMS1-4 CRC patient tumors. Replicates in (A–E), AP (tumors), n = 3; APN (tumors), n = 3; APN (organoids), n = 4; KP (tumors), n = 3; KPN (tumors), n = 9; KPN (organoids), n = 3.

See also Figures S3 and S4 and Tables S3, S4, and S5.

5A and S4E), implying that they may not be determinants of metastatic spread. Importantly, enrichment of a neutrophil signature was associated with metastatic KPN tumors, but not with non-metastatic KP tumors (Figures 5B and S4E). Similar to human serrated adenoma, we have detected neutrophil accumulation in primary tumors, metastases, and systemically in KPN mice (Figures 5C–5F). Given that metastasis in the KPN model was associated with neutrophil infiltration, we assessed expression of chemokines implicated in neutrophil attraction (Figures 5G and S5A) finding increased expression of *Cxcl5* in the epithelium of KPN tumors but not of KP (Figures 5G and 5H and Table S6). *Cxcl5* expression was correlated with that of its receptor *Cxcr2*, which is predominantly expressed on neutrophils (Figure S5B). When we examined the expression of neutrophil-associated genes such as *ELANE*, *MPO*, and *CXCR2* in human CRC, we found significantly increased expression in human CMS4 (Figure S5C). In addition, the neutrophil infiltration-score was able to predict survival in treatment-naive metastases and is significantly associated with CMS4 and CRIS-B (Figures S5D and S5E). Furthermore, neutrophil infiltration of metastases, analyzed by MPO or CXCR2 expression predicts poor survival in an additional cohort (Figure S5F; Table S6). For these reasons, we hypothesized that neutrophils may be a critical driver of NOTCH1-dependent metastasis in CMS4/CRIS-B CRC.

To test this, we treated KPN mice with AZD5069, a clinically relevant CXCR2 small-molecule (CXCR2sm) inhibitor (Nicholls et al., 2015), which has been shown to block neutrophil recruitment. Importantly, while CXCR2sm treatment, from day 85

(E) Schematic representation of LGK974 treatments started 85 days after induction.

(F) Kaplan-Meier survival curves of KPN mice after treatment as indicated in (E).

(G) Incidence of metastases per treatment; in (F) and (G): vehicle, n = 7; LGK974, n = 8.

(H) Representative pictures of organoid cultures. R, R-spondin; E, EGF; N, Noggin. Scale bars, 100 μm.

(I) Organoid size or number 7 days after single-cell seeding under the indicated conditions. Samples were generated from individual tumors, n = 3.

(J) Organoid size or number 7 days after single-cell seeding under the indicated conditions. Samples were generated from individual tumors, n = 4.

(K) Analysis of the consequences of somatic mutations identified by whole-genome sequencing of KPN primary tumor-derived organoids.

Error bars in (A), (D), (I), and (J) represent mean ±SEM. Data in (A) and (D) analyzed by Mann-Whitney U test, two-tailed. See also Figures S3 and S4 and Tables S1 and S2.

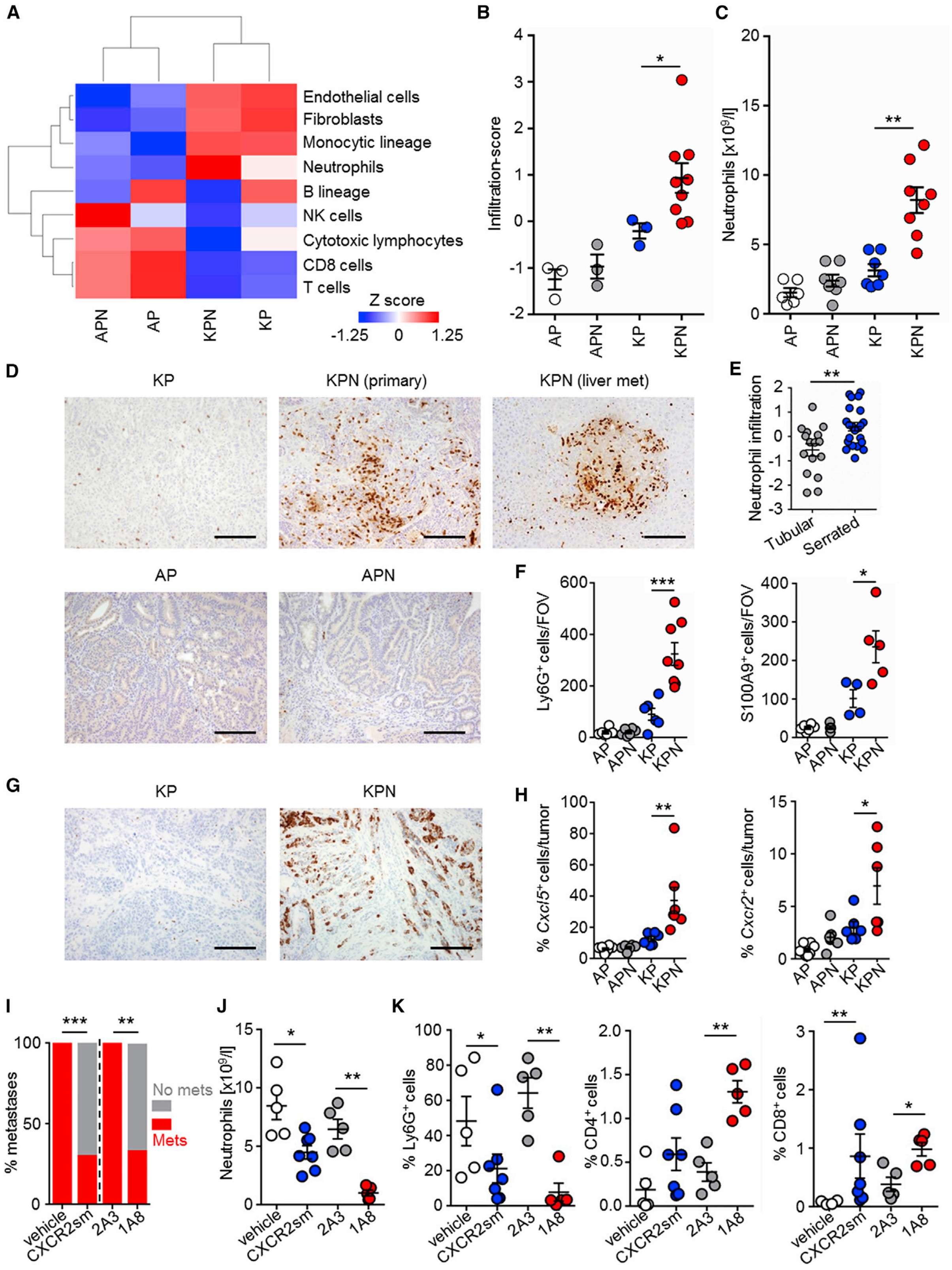

Figure 5. Epithelial NOTCH1 Controls Neutrophil Recruitment to Drive Metastasis

(A) Heatmap showing standardized infiltration-scores (calculated with MCPcounter) in GEMM tumors; AP, n = 3; APN, n = 3; KP, n = 3; KPN, n = 9.

(B) Dot-plots showing standardized infiltration scores of neutrophils (calculated with MCPcounter); replicates as in (A).

(legend continued on next page)

post induction, did not impact survival or primary tumor burden in KPN mice, it profoundly reduced metastasis (Figures 5I, S6A, and S6B; Table S1). Short term treatment of KPN tumor-bearing mice with CXCR2sm resulted in reduced neutrophil counts in both the peripheral blood and primary tumors (Figures 5J and 5K), and an increase in $CD8^+$ T cell numbers, compared with vehicle-treated counterparts (Figures 5K and S6C–S6E). The enhanced $CD8^+$ T cell number, thought to create an anti-metastatic microenvironment at a secondary site, was retained in livers at endpoint (Figure S6F).

Given that CXCR2 expression may not be restricted to neutrophils, we evaluated the impact of neutrophil depletion with a Ly6G-targeting antibody (1A8). Again, metastasis was suppressed when compared with isotype control (2A3), but survival was unaffected (Figures 5I, S6G, and S6H; Table S1). Circulating neutrophils were reduced at endpoint, indicating a sustained effect of the neutralizing antibody (Figure S6I). We detected an increase in $CD4^+$ and $CD8^+$ T cells in the primary tumors of KPN mice treated with 1A8 in the short term (Figures 5K, S6J, and S6K), and increased $CD8^+$ T cells in livers at endpoint (Figure S6L). Together, this indicates that epithelial NOTCH1 triggers CXCR2-dependent $Ly6G^+$ neutrophil accumulation within the pre-metastatic niche and generates an immunosuppressive environment. Therapeutic targeting of neutrophils results in increased infiltrating $CD8^+$ T cells within the pre-metastatic niche and a reduction in metastasis.

Epithelial NOTCH1 Signature Predicts Poor Survival and Drives Epithelial TGF-β2 Expression

To determine how epithelial NOTCH1 controls metastasis, we examined the transcriptome of tumor-derived KPN versus KP organoids (Figure 6A; Table S7). Increased expression of canonical NOTCH1 target genes, such as *Fjx1* and *Dtx1*, along with an enriched NOTCH-score was observed in KPN organoids, compared with KP counterparts (Figures 6A and S7A). Strikingly in human CRC, the KPN/KP-score predicts poor prognosis and is associated with CMS4, CRIS-B, and neutrophil infiltration (Figures 6B and S7B–S7E). Interestingly, we found significantly increased expression of the gene encoding the *Tgfb2* ligand in KPN organoids (Figure 6A). *TGFB2* expression predicts poor survival and is significantly correlated with CRIS-B, CMS4, NOTCH-score, and KPN/KP-score in human CRC datasets (Figures 6B, 6C, and S7F–S7J). Furthermore, *TGFB2* expression and the KPN/KP-score are also associated with human serrated adenoma (Figure 6D). Together, these data demonstrate a strong association between epithelial NOTCH1-dependent transcriptional signatures and high *TGFB2* expression in serrated tumors which exhibit poor outcome and underscores the human relevance of the KPN model.

To understand how NOTCH1 controls *Tgfb2* expression we confirmed NOTCH1-dependent expression of *Tgfb2* in KPN organoids (Figure 6E). Moreover, GSEA showed that KPN primary tumors and organoids are associated with TGF-β activation (Figures 4E and 6F). Promoter analysis of the genomic area around the *Tgfb2* transcriptional start site for putative RBPJ binding sites revealed a number of canonical RBPJ-DNA binding motifs (Figure 6G; Table S8). Chromatin immunoprecipitation of RBPJ, the key mediator of NOTCH1-mediated transcription, showed binding to the promoter of *Tgfb2* in KPN organoids; however, no binding was detected to an upstream region of the *Tgfb2* promoter which lacks RBPJ binding sites or a control region (*Chrm1*) (Figure 6H).

Alongside the upregulated neutrophil markers in the primary tumors from our GEMMs (Figures 5A–5D and 6I), *Tgfb2* was expressed by epithelial cells in KPN tumors (Figure 6J). *Tgfb1* expression was comparable in both primary tumors and organoids derived from KPN or KP mice (Figures 6A, 6E and 6I), with predominantly stromal expression in KPN tumors (Figure S7K).

Neutrophil Inhibition Attenuates Metastasis by T Cell Activation

We next examined the contribution that the epithelial compartment of KPN tumors makes to the TME in metastatic colonization. Previous studies have reported that intra-splenic transplantation of organoids from mouse intestinal tumors, with combined *Apc*, $Kras^{G12D}$, *Trp53*, and TGF-β signaling mutations, is the most efficient means of generating metastases (Tauriello et al., 2018; Sakai et al., 2018) (Figure 7A). We found no difference in the capacity to colonize the liver between organoids derived from KPN or *villin*Cre^{ER} $Apc^{fl/fl}$ $Kras^{G12D/+}$ $Trp53^{fl/fl}$ $Trgfbrl^{fl/fl}$ (AKPT) tumors (Figure 7B), but found a significant increase in $Ly6G^+$ neutrophils in liver metastases formed by KPN organoids (Figures 7C and 7D), and strong epithelial expression of *Tgfb2* only in KPN liver metastases (Figure 7E).

To investigate the impact of neutrophils on metastatic seeding of KPN organoids in this transplantation model we treated recipient mice with either CXCR2sm or a TGFBR1/ALK5 kinase inhibitor (Alk5i) (Figure 7F). Strikingly, both inhibitors reduced the metastatic number and burden significantly (Figures 7G and S7L), and, as observed in the autochthonous GEMM (Figure S6C), long-term treatment with CXCR2sm led to a

(C) Blood neutrophil count at endpoint of indicated genotype (n ≥ 6).
(D) Representative Ly6G IHC. Scale bars, 100 μm.
(E) Neutrophil infiltration-score in human adenoma.
(F) Quantification of $Ly6G^+$ and $S100A9^+$ cells per field of view (FOV); AP, n = 6; APN, n ≥ 5; KP, n ≥ 4; KPN, n ≥ 5.
(G) Representative ISH of *Cxcl5* expression. Scale bars, 100 μm.
(H) Quantification of *Cxcl5*$^+$ and *Cxcr2*$^+$ cells; AP, n = 8; APN, n = 6; KP, n = 6; KPN, n ≥ 6.
(I) Incidence of metastases at endpoint for KPN mice treated with: vehicle, n = 11; CXCR2sm, n = 10; 2A3, n = 10; 1A8, n = 9; analyzed by chi-square test, two-tailed.
(J) Blood neutrophil count after 1 week of indicated treatments: vehicle, n = 5; CXCR2sm, n = 7; 2A3, n = 5; 1A8, n = 5; analyzed by Mann-Whitney U test, one-tailed.
(K) Quantification of IHC on primary tumors of KPN mice after 1 week of indicated treatments: vehicle, n ≥ 4; CXCR2sm, n = 7; 2A3, n = 5; 1A8, n = 5.
Error bars in (B), (C), (E), (F), (H), (J), and (K) represent mean ± SEM. Data in (B), (C), (E), (F), (H), and (K) analyzed by Mann-Whitney U test, two-tailed. See also Figures S5 and S6 and Tables S1 and S6.

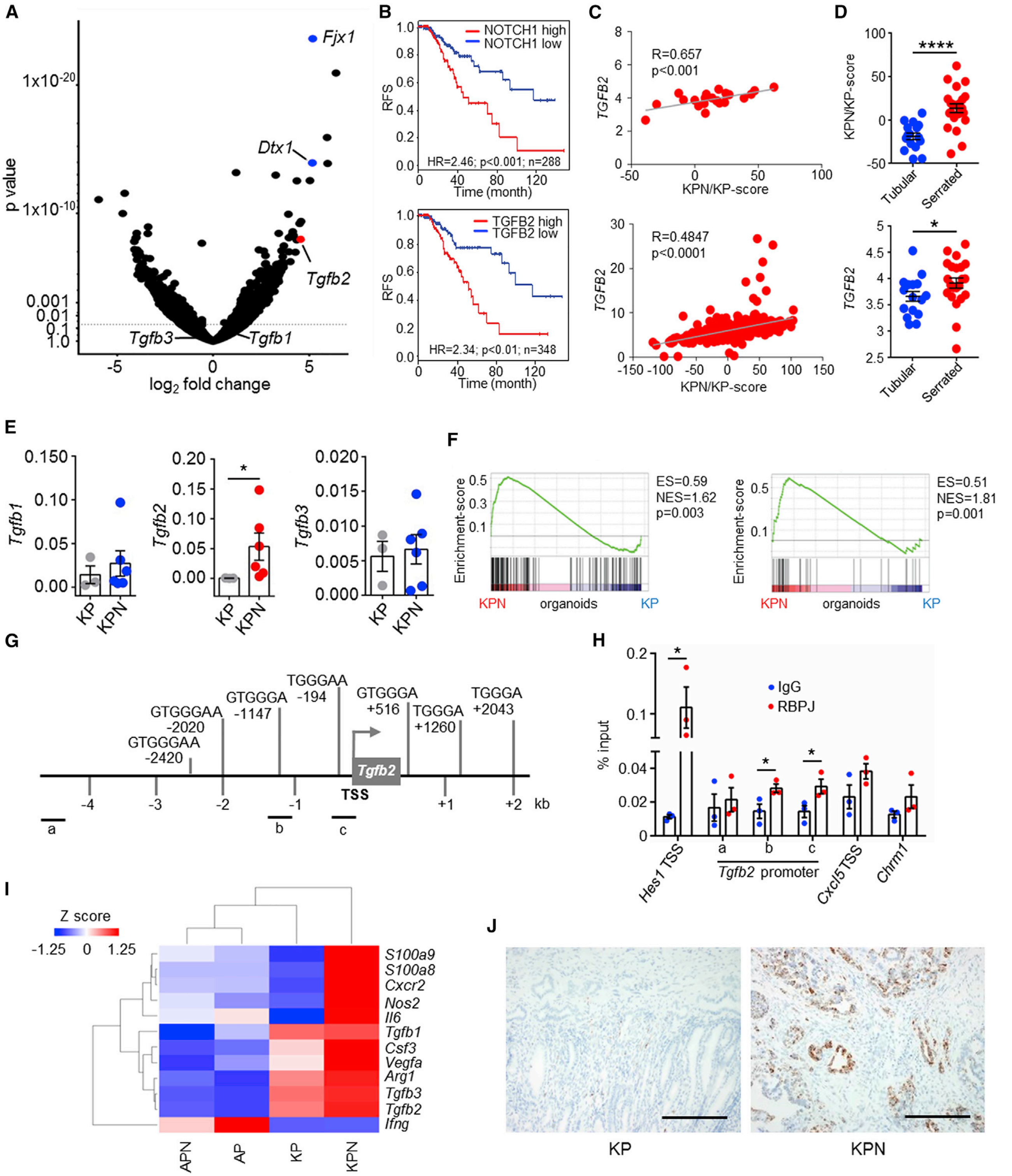

Figure 6. Epithelial NOTCH1 Drives Poor Prognosis Signatures and TGF-β2 Expression

(A) Volcano-plot of organoid KPN (n = 3) versus KP (n = 3) mRNA expression.

(B) RFS of CRC patients (TCGA), stratified using the KPN/KP-score as in (A) or *TGFB2* expression. The blue line shows expression ≤ median score (low), the red line shows expression > median score (high).

(C) Correlation of the KPN/KP-score and *TGFB2* expression in human serrated adenoma (top) or in TCGA data (bottom), p values were calculated by Pearson correlation.

(D) KPN/KP-score or expression of *TGFB2* in human adenoma.

(legend continued on next page)

significant increase of circulating neutrophils (Figure S7M). Intriguingly, Alk5i treatment had no effect on circulating neutrophils but significantly reduced neutrophils infiltrating metastases (Figures 7H and 7I). In addition, we detected an increase in activated CD69$^+$/CD4$^+$, CD69$^+$/CD8$^+$, and IFNγ^+ CD4$^+$ type 1 T helper (Th1) cells upon Alk5i treatment (Figure 7J). Importantly, we detected increased CD8$^+$ GzB$^+$ cytotoxic T lymphocytes in metastases treated with Alk5i or CXCR2sm (Figures 7J, S7N, and S7O). This suggests that the effect of CXCR2sm and Alk5i on metastasis is mediated by alleviation of a neutrophil-dependent immunosuppressive microenvironment. This is supported by the finding that CXCR2sm or Alk5i have no effects on metastasis when KPN organoids were transplanted into immune-deficient nude mice which lack T cells (Figure S7P). Together, these data show that the epithelial programs driven by NOTCH1 in KPN tumor cells rewire the TME and generate an immunosuppressive, pro-metastatic environment.

Inhibition of Neutrophil TGF-β Signaling Attenuates Metastasis

Given the NOTCH1-dependent expression of *Tgfb2* in the KPN model and the profound effect on metastasis in the transplantation model, we examined the importance of TGF-β signaling to NOTCH1-dependent metastasis. We confirmed TGF-β activity in KPN tumors via nuclear localization of phosphorylated SMAD3 (pSMAD3) and TGF-β signaling targets *Smad7*, CALD1, and IGFBP7 (Figure 8A). We then applied two independent, clinically relevant therapeutic approaches, either targeting of TGFBR1/ALK5 with Alk5i, or with a ligand-trapping antibody targeting TGF-β1/2/3 (1D11) (Figure 8B). In an early-intervention setting, in which mice are treated from 85 days after induction, inhibition of ALK5 resulted in rapid development of highly invasive (T3) colonic tumors, although with markedly reduced metastasis (Figures 8B and 8C; Table S1). Interestingly, targeting the ligands with 1D11 did not result in accelerated tumorigenesis, but significantly reduced metastasis (Figures 8B and 8C; Table S1). In a late intervention approach, when mice were treated with Alk5i from 130 days after induction, developing tumors with a similar latency and comparable tumor burden as vehicle-treated mice (Figures 8B and S8A), but with significantly reduced metastatic penetrance at endpoint (Figure 8C; Table S1). This was associated with a significant reduction in the number of neutrophils in the liver (Figure 8D), with peripheral blood neutrophils being unaffected (Figure S8B). The reduction in liver neutrophils was accompanied by accumulation of CD3$^+$, CD4$^+$, and CD8$^+$ T cells (Figures 8D, S8C, and S8D). Interestingly, no change in primary tumor fibrosis was detected when KPN mice were treated with Alk5i or 1D11 (Figure S8D). Taken together, these data support a strong role for TGF-β signaling in generating an immunosuppressive pro-metastatic microenvironment in the liver by recruiting neutrophils.

To address the specific function of TGF-β signaling in the neutrophil population of tumor-bearing mice, we transplanted KPN organoids into the colonic submucosa of syngeneic C57BL/6 mice lacking *Alk5* expression specifically in Ly6G$^+$ neutrophils (*Ly6G*^{Cre} *Alk5*$^{fl/fl}$) (Figure 8E). Engrafted KPN organoids formed primary tumors that were morphologically indistinguishable from those of the autochthonous GEMM (Figures 8F, S8E, and S8F), with epithelial cells from primary tumors and liver metastases also found to express high levels of *Tgfb2* (Figure 8G). As predicted by inhibitor experiments, deletion of *Alk5* in the neutrophil population had no beneficial impact on survival (Figure S8G), but led to a striking reduction in metastasis (Figure 8H). This was concomitant with reduced tumor-infiltrating neutrophils, although peripheral blood neutrophil counts were maintained (Figure 8I). Reduced neutrophil infiltration was not associated with an alteration in neutrophil maturity, as expression of CD101 (a marker of mature murine neutrophils) was unaffected by ALK5 deficiency (Figure 8J). Importantly, TGF-β signaling inhibition blocks metastasis by reducing neutrophil attraction, rather than by polarization to an anti-tumor phenotype.

DISCUSSION

The genetic progression of CRC has been investigated for many years, yet no robust drivers of metastasis have been identified in GEMMs. Importantly, we demonstrate that alteration of epithelial tumor cell-intrinsic signaling can rewire the TME and, in turn, promote metastasis. Notably, this occurs spontaneously only in concert with specific mutations that drive serrated tumors, and not during the progression of WNT-driven tubular adenoma. These observations are consistent with reported differential responses of tubular and serrated adenomas to TGF-β signaling (Fessler et al., 2016).

Whole-genome sequencing of tumors from KPN mice revealed relatively low levels of mutation, although recurrent biallelic mutations in *Csnk1a1* were observed. This would suggest a further single sporadic mutation, for example in *Csnk1a1*, could drive rapid progression to adenocarcinoma and metastasis in the KPN model, possibly by generating an inflammatory TME (Pribluda et al., 2013). One could hypothesize that this recapitulates the "Big Bang" model of human CRC in which the key driver mutations occur early, while later, large tumors exhibit neutral evolution (Sottoriva et al., 2015).

The literature regarding NOTCH and WNT interaction in WNT-driven models reveals different roles of NOTCH1 signaling. Epithelial N1icd expression, in combination with *Apc*^{1263} allelic mutation, can drive tumor initiation (Fre et al., 2009), or inhibit

(E) qPCR from KP or KPN organoids (n $\geq$ 3); normalized to *Actb*.

(F) GSEA plots of TGF-β activation (left, GSE15871; right, GSE39397) in KP versus KPN organoids. ES, enrichment score; NES, normalized enrichment score.

(G) Schematic representation of RBPJ binding sites at the mouse *Tgfb2* promoter. TSS, transcription start site.

(H) Chromatin immunoprecipitation of RBPJ and IgG control in KPN organoids; n = 3 biological replicates of technical duplicates, analyzed by Student's t test, two-tailed.

(I) Heatmap of marker expression across primary tumors.

(J) Representative images of *Tgfb2* ISH in primary tumors. Scale bars, 100 μm.

Error bars in (D), (E), and (H) represent mean ± SEM. Data in (D) and (E) analyzed by Mann-Whitney U test, two-tailed. See also Figure S7 and Tables S7 and S8.

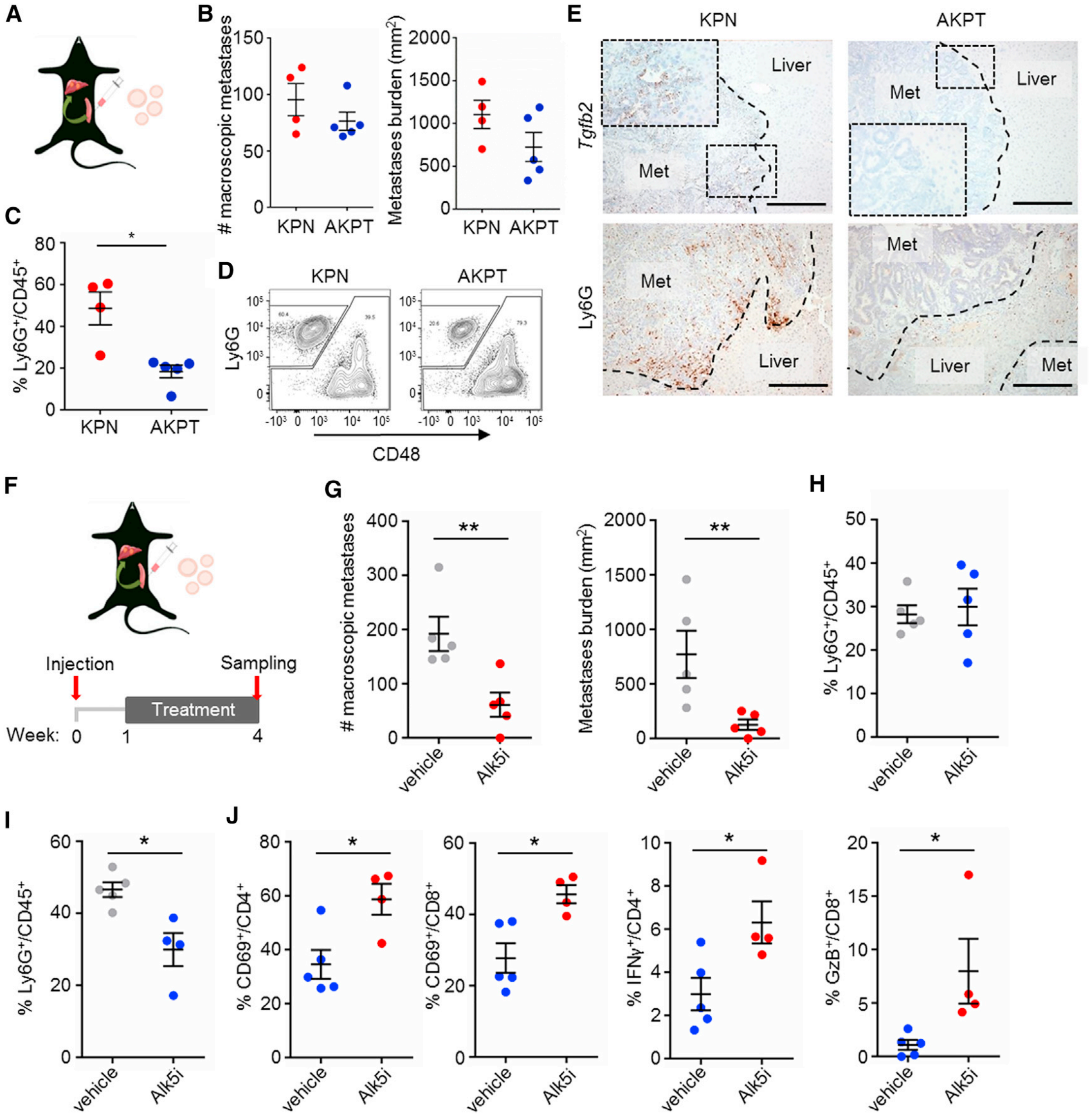

Figure 7. TGF-β or CXCR2 Inhibition Attenuates KPN Metastasis via T Cell Activation

(A) Cartoon illustrating organoid isograft transplantation in the spleen.

(B) Quantification of macroscopic liver metastases 4 weeks post-transplantation; KPN, n = 4; AKPT, n = 5.

(C) Quantification of neutrophils in liver metastases by flow cytometry as in (B).

(D) Representative contour plots of the analysis performed in (C).

(E) Representative images for ISH analysis of *Tgfb2* expression or IHC for Ly6G on liver metastases (n ≥ 3). Scale bars, 100 μm.

(F) Schematic representation of the treatment regimen after organoid transplantation.

(G) Number and burden of macroscopic liver metastases 4 weeks post-KPN organoid transplantation; vehicle, n = 5; Alk5i, n = 5.

(H and I) Quantification of flow cytometry analysis for neutrophils in blood (H) or liver metastases 4 weeks post-KPN organoid transplantation (I); vehicle, n = 5; Alk5i, n ≥ 4.

(J) Quantification of flow cytometry analysis for T cell subsets in liver metastases 4 weeks post-KPN organoid transplantation; vehicle, n = 5; Alk5i, n = 4.

Error bars in (B), (C), (G), (H), (I), and (J) represent mean ± SEM. Data in (C), (G), (I), and (J) analyzed by Mann-Whitney U test, two-tailed. See also Figure S7.

WNT signaling in the *Apc*$^{Min/+}$ model at the transcriptional level (Kim et al., 2012). Our results demonstrate that, in the context of *Apc* and *Trp53* loss, N1icd has no significant impact on survival or tumor stage. It is interesting to note, however, that we observed reduced levels of selected WNT target genes in KPN tumors and that a specific WNT pathway mutation was selected in emerging tumors. Importantly, human serrated *KRAS*-mutant tumors predominantly exhibit low levels of nuclear β-catenin (Bennecke et al., 2010), mirrored in KPN tumors. This demonstrates that the model described here shares key cellular and molecular features with human serrated disease. We speculate that WNT ligand inhibition might be a therapeutic option for this type of CRC as reported for CRC with RNF43 mutations or R-spondin fusions (Storm et al., 2016; Yan et al., 2017; Han

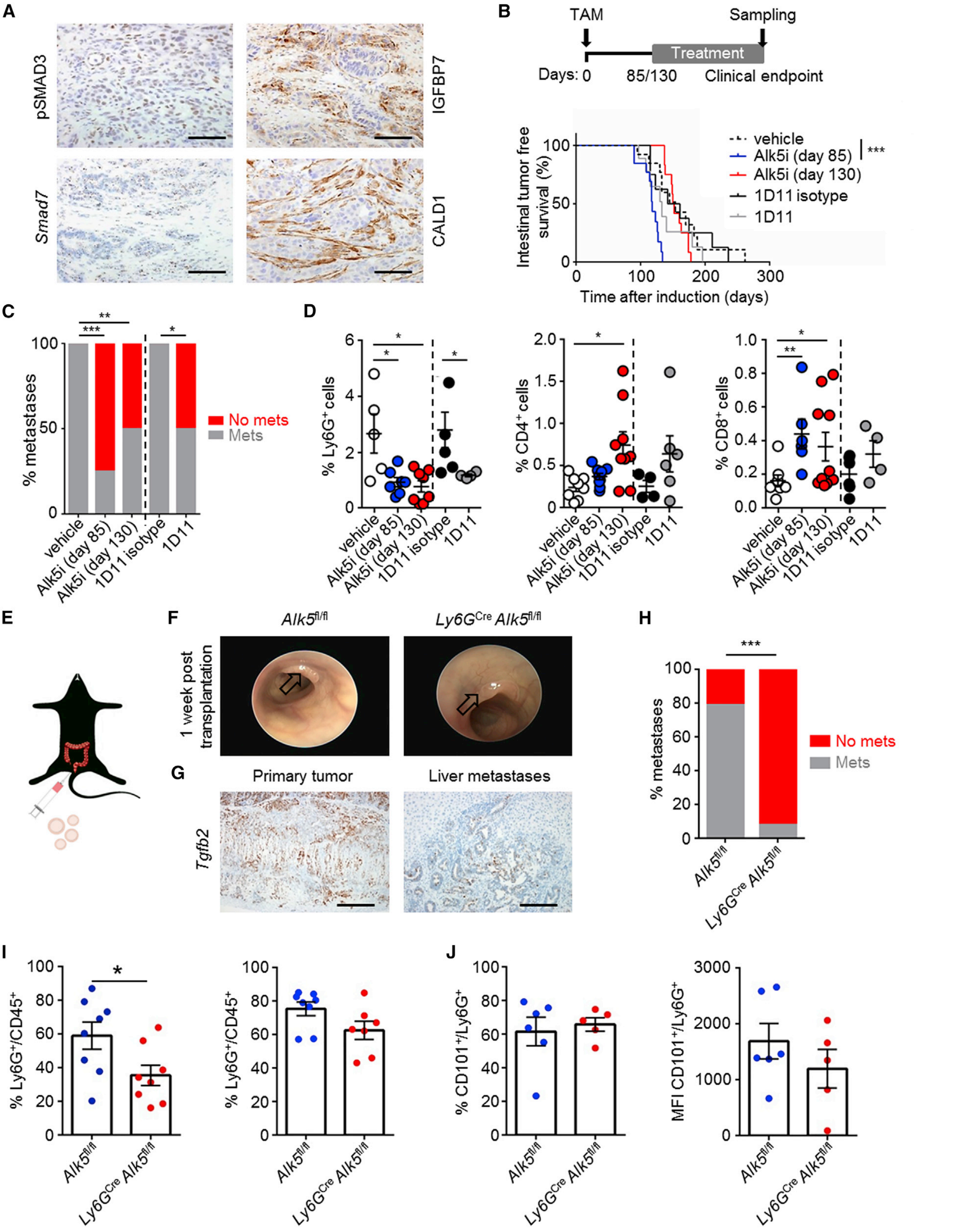

(legend on next page)

et al., 2017). This said, we found no response of these tumors to PORCUPINE inhibition. From our work, it is interesting to propose that the timing of WNT pathway activation is key. When *Apc* is lost early, adenoma/polyps are formed that require multiple further mutations to progress to adenocarcinoma (Sakai et al., 2018). In a serrated model, WNT pathway mutations occur much later (which happens in the KPN model) and drive rapid progression to carcinoma and metastasis.

Critically, our model suggests therapeutic targets in mCRC. NOTCH inhibitors are currently in non-stratified clinical trials for CRC and have shown some minor benefits (Andersson and Lendahl, 2014; Meurette and Mehlen, 2018); our data suggest benefits for NOTCH signaling inhibition in CMS4/CRIS-B patients. Combinatorial inhibition of MEK/ERK and γ-secretase increases efficacy in melanoma, papillary thyroid cancer, and CRC (Krepler et al., 2016; Yamashita et al., 2013; Schmidt et al., 2018). Identification of tumors with activated NOTCH1 signaling may be aided with the KPN/KP-score described here, or through use of TGF-β2 as surrogate. To generate the NOTCH1 signature we activated N1icd in intestinal enterocytes, mimicking the activation of NOTCH1 signaling in CRC by NOTCH1 receptor copy gain (Arcaroli et al., 2016). Activation of NOTCH1 in the KPN model had no impact upon normal homeostasis, possibly because of targeted activation of NOTCH1 in adult tissue rather than during embryonic development (Fre et al., 2005). Interestingly, lateral inhibition, which occurs in the normal small intestinal crypt to maintain the stem cell niche, could influence the dynamics of non-cell-autonomous NOTCH1 activation and may occur in malignant progression. This kind of non-cell-autonomous activation should be considered in the future.

Our work also elucidates a novel non-cell-autonomous role for NOTCH1 in CRC through control of chemokine expression (TGF-β2 and CXCL5). Interestingly, TGF-β2 shows a 100- to 500-fold higher affinity to betaglycan/TGFBRIII than TGF-β1/3 (Cheifetz et al., 1990). Previously, NOTCH1 signaling has been shown to impact the cellular secretome of multiple cancers (Hoare et al., 2016; Wieland et al., 2017; Shen et al., 2017). Interestingly, this cancer-associated secretome includes many inflammatory chemokines, similar to the KPN tumors, such as granulocyte-colony stimulating factor and interleukin-6. The potential release of numerous chemokines may help to explain the dramatic effects on neutrophils both in the blood and at metastatic sites in our model. Non-metastatic KP tumors develop a similarly fibroblast-rich stroma as KPN tumors, but lack TGF-β2 expression and neutrophil infiltration. Therefore, the TME generated by CAFs appears insufficient on its own to prime for metastasis but relies on the infiltration of neutrophils. This suggests that our GEMMs may differ from some of the recently described models in which CAFs were important in defining TGF-β sensitivity (Calon et al., 2012; Tauriello et al., 2018). Interestingly, these models were initiated by *Apc* loss (alongside *Kras*, *Tp53*, and *Smad4*/*TgfbrII*) and predominantly driven by transplantation or induced colitis. It would be interesting to examine the chemokine profiles of these tumors as they may well be differentially dependent on contributions from components of the TME, as indicated by the difference in TGF-β2 expression.

Though previously thought to be terminally differentiated cells, neutrophils exhibit phenotypic plasticity and adopt distinct mature phenotypes (Leach et al., 2019). Accordingly, neutrophils have recently been shown to express various markers of maturity (Evrard et al., 2018). In particular, TGF-β signaling is thought to be a key regulator of the pro-tumorigenic state of neutrophils, and TGF-β inhibition has been shown to drive increased tumor infiltration of neutrophils (Fridlender et al., 2009). This is in contrast to our findings, where pharmacological inhibition of TGF-β signaling and genetic deletion of TGF-β signaling activity in neutrophils reduces neutrophil numbers in tumors but has no effect on neutrophil maturation status.

Future studies should characterize the phenotypic traits of neutrophils in the metastatic niche, when compared with the primary tumor and peripheral blood. Our findings suggest that CXCR2/ALK5-expressing neutrophils are important in CMS4/CRIS-B CRC disease progression and in the genesis of CRC metastases. The unresponsiveness of primary tumors to CXCR2sm treatment pre-clinically in this study and other studies (Steele et al., 2016) might be explained by active immune checkpoints in the primary site, which are lacking in the metastatic site. It should be noted that our mouse model shows high systemic levels of neutrophils and that clinically high neutrophil-to-lymphocyte ratios (NLR) are often associated with the poorest prognosis (Roxburgh et al., 2010). It would be of interest to determine whether these NLRs could be used to stratify patients for CXCR2/TGF-β/NOTCH-targeted therapies. In particular, the sensitivity of metastases to neutrophil inhibition holds promise as a potential treatment option for stage II/III CRC patients with undergoing primary tumor resection before treatment with CXCR2 or ALK5 inhibitors. Critically, we highlight a novel targeted therapeutic approach which may compromise the seeding of metastases in a setting in which major primary

Figure 8. Inhibition of Neutrophilic TGF-β Signaling Attenuates Metastasis

(A) Representative images of indicated markers on KPN primary tumors. Scale bars, 100 μm.

(B) Schematic representation of treatment regime and Kaplan-Meier survival curves of KPN mice treated with: vehicle day 85, n = 13; Alk5i day 85, n = 13; Alk5i day 130 , n = 12; 1D11 isotype day 85, n = 8; 1D11 day 85, n = 9; analyzed by log rank (Mantel-Cox) test.

(C) Incidence of metastasis per indicated treatments: vehicle day 85, n = 11; Alk5i day 85, n = 12; Alk5i day 130, n = 12; 1D11 isotype day 85, n = 8; 1D11 day 85, n = 8. Analyzed by chi-square test, two-tailed.

(D) Quantification of IHC for Ly6G$^+$, CD4$^+$, and CD8$^+$ cells per KPN liver at endpoint (n $\geq$ 4).

(E) Cartoon illustrating intra-colonic transplantation of KPN organoids.

(F) Representative colonoscopy images 1 week post-transplantation. Arrows indicate tumors.

(G) Representative ISH on transplanted KPN organoids. Scale bars, 100 μm.

(H) Incidence of metastases at endpoint; *Alk5*$^{fl/fl}$, n = 14; *Ly6G*^{Cre} *Alk5*$^{fl/fl}$, n = 13. Analyzed by chi-square test, two-tailed.

(I) Flow cytometry analysis of neutrophils in primary tumors (left) and peripheral blood (right); *Alk5*$^{fl/fl}$, n = 8; *Ly6G*^{Cre} *Alk5*$^{fl/fl}$, n $\geq$ 7.

(J) Flow cytometry analysis of CD101$^+$ neutrophils in primary tumors; *Alk5*$^{fl/fl}$, n = 6; *Ly6G*^{Cre} *Alk5*$^{fl/fl}$, n = 5; MFI, mean fluorescence intensity.

Error bars in (D), (I), and (J) represent mean ± SEM, analyzed by Mann-Whitney U test, two-tailed. See also Figure S8 and Table S1.

and metastatic tumor burden is reduced through surgical resection.

STAR★METHODS

Detailed methods are provided in the online version of this paper and include the following:

- KEY RESOURCES TABLE
- CONTACT FOR REAGENT AND RESOURCE SHARING
- EXPERIMENTAL MODEL AND SUBJECT DETAILS
  - Animals
  - Tumor Models and Treatments
  - Patient Material
- METHOD DETAILS
  - Scoring of Tumor Stage and Differentiation
  - Blood Count Analysis
  - Organoid Culture
  - Single Cell Seeding
  - Intra-Splenic Injection of Organoids
  - Needle-Guided Intracolonic Organoid Transplantation
  - Sample Processing and Staining for Flow Cytometry
  - RNA Isolation
  - qRT-PCR
  - Chromatin Immunoprecipitation
  - Immunohistochemistry
  - *In Situ* Hybridisation
  - RNA-Sequencing
  - Tumor and Metastasis Scoring
  - Image Analysis
  - CRC Patient Data for *In Silico* Analysis
  - Mouse Model and Patient Gene Expression Signatures
  - Infiltration Scores
  - KPN VS. APN Signature Survival Analysis
  - GSEA
  - NOTCH-score
  - WNT-score
  - KPN/KP-score
  - Human Serrated Signature
  - Whole Genome Sequencing
  - Somatic Mutation Detection
  - Copy Number Detection
- DATA AND CODE AVAILABILITY
- QUANTIFICATION AND STATISTICAL ANALYSIS

SUPPLEMENTAL INFORMATION

Supplemental Information can be found online at https://doi.org/10.1016/j.ccell.2019.08.003.

ACKNOWLEDGMENTS

We thank the Core Services and Advanced Technologies at the Cancer Research UK Beatson Institute (C596/A17169), and particularly the Biological Services Unit, Histology and Molecular Technologies. We are thankful to members of the Sansom lab for discussion and Catherine Winchester for editing the manuscript. O.J.S. and his lab members were supported by CRUK grants A26825, A28233, A23390, A21139, A12481, and A17196 and an ERC Starting Grant 311301. R.J. was supported by the Marie Skłodowska-Curie Actions postdoctoral fellowship (ERC 659666). J.D.L. was supported by an MRC Clinical Research Training Fellowship (MR/N021800/1). J.P.M. and S.v.H. were supported by grants from the Dutch Cancer Society (Oncode, 10150, 10562).

AUTHOR CONTRIBUTIONS

R.J., S.R.v.H., J.P.M., and O.J.S. designed the research. R.J., J.O.L., S.R.v.H., X.C.-L., C.Nixon, J.O.L., R.A.R., V.M.W., W.C., C.Nourse, M.R., and A.H. performed the experiments and analyzed the data. C.W.S., T.J.K., C.S.R., P.G.H., A.D.C., D.J.A., P.B., A.V.B., M.G., J.R., O.H.Y., and S.T.B. contributed new reagents and/or analytical tools. R.J. and O.J.S. wrote the paper, all authors read the manuscript and provided critical comments.

DECLARATION OF INTERESTS

Simon T. Barry is an employee and shareholder of AstraZeneca.

Received: December 17, 2018
Revised: May 31, 2019
Accepted: August 6, 2019
Published: September 16, 2019

REFERENCES

Anders, S., and Huber, W. (2010). Differential expression analysis for sequence count data. Genome Biol. *11*, R106.

Andersson, E.R., and Lendahl, U. (2014). Therapeutic modulation of Notch signalling–are we there yet? Nat. Rev. Drug Discov. *13*, 357–378.

Anderton, M.J., Mellor, H.R., Bell, A., Sadler, C., Pass, M., Powell, S., Steele, S.J., Roberts, R.R., and Heier, A. (2011). Induction of heart valve lesions by small-molecule ALK5 inhibitors. Toxicol. Pathol. *39*, 916–924.

Arcaroli, J.J., Tai, W.M., McWilliams, R., Bagby, S., Blatchford, P.J., Varella-Garcia, M., Purkey, A., Quackenbush, K.S., Song, E.K., Pitts, T.M., et al. (2016). A NOTCH1 gene copy number gain is a prognostic indicator of worse survival and a predictive biomarker to a Notch1 targeting antibody in colorectal cancer. Int. J. Cancer *138*, 195–205.

Babaei-Jadidi, R., Li, N., Saadeddin, A., Spencer-Dene, B., Jandke, A., Muhammad, B., Ibrahim, E.E., Muraleedharan, R., Abuzinadah, M., Davis, H., et al. (2011). FBXW7 influences murine intestinal homeostasis and cancer, targeting Notch, Jun, and DEK for degradation. J. Exp. Med. *208*, 295–312.

Batlle, E., Bacani, J., Begthel, H., Jonkheer, S., Gregorieff, A., van de Born, M., Malats, N., Sancho, E., Boon, E., Pawson, T., et al. (2005). EphB receptor activity suppresses colorectal cancer progression. Nature *435*, 1126–1130.

Becht, E., De Reynies, A., Giraldo, N.A., Pilati, C., Buttard, B., Lacroix, L., Selves, J., Sautes-Fridman, C., Laurent-Puig, P., and Fridman, W.H. (2016a). Immune and stromal classification of colorectal cancer is associated with molecular subtypes and relevant for precision immunotherapy. Clin. Cancer Res. *22*, 4057–4066.

Becht, E., Giraldo, N.A., Lacroix, L., Buttard, B., Elarouci, N., Petitprez, F., Selves, J., laurent-Puig, P., Sautes-Fridman, C., Fridman, W.H., and de Reynies, A. (2016b). Estimating the population abundance of tissue-infiltrating immune and stromal cell populations using gene expression. Genome Biol. *17*, 218.

Bennecke, M., Kriegl, L., Bajbouj, M., Retzlaff, K., Robine, S., Jung, A., Arkan, M.C., Kirchner, T., and Greten, F.R. (2010). Ink4a/Arf and oncogene-induced senescence prevent tumor progression during alternative colorectal tumorigenesis. Cancer Cell *18*, 135–146.

Boeva, V., Popova, T., Bleakley, K., Chiche, P., Cappo, J., Schleiermacher, G., Janoueix-Lerosey, I., Delattre, O., and Barillot, E. (2012). Control-FREEC: a tool for assessing copy number and allelic content using next-generation sequencing data. Bioinformatics *28*, 423–425.

Borowsky, J., Dumenil, T., Bettington, M., Pearson, S.A., Bond, C., Fennell, L., Liu, C., Mckeone, D., Rosty, C., Brown, I., et al. (2018). The role of APC in WNT pathway activation in serrated neoplasia. Mod. Pathol. *31*, 495–504.

Calon, A., Espinet, E., Palomo-Ponce, S., Tauriello, D.V., Iglesias, M., Cespedes, M.V., Sevillano, M., Nadal, C., Jung, P., Zhang, X.H., et al.

(2012). Dependency of colorectal cancer on a TGF-beta-driven program in stromal cells for metastasis initiation. Cancer Cell *22*, 571–584.

Calon, A., Lonardo, E., Berenguer-Llergo, A., Espinet, E., Hernando-Momblona, X., Iglesias, M., Sevillano, M., Palomo-Ponce, S., Tauriello, D.V., Byrom, D., et al. (2015). Stromal gene expression defines poor-prognosis subtypes in colorectal cancer. Nat. Genet. *47*, 320–329.

Cancer Genome Atlas Network. (2012). Comprehensive molecular characterization of human colon and rectal cancer. Nature *487*, 330–337.

Carlson, M. 2017. org.Hs.eg.db: Genome wide annotation for Human. R package version 3.4.0. 10.18129/B9.bioc.org.Hs.eg.db

Chanrion, M., Kuperstein, I., Barriere, C., el Marjou, F., Cohen, D., Vignjevic, D., Stimmer, L., Paul-Gilloteaux, P., Bieche, I., Tavares Sdos, R., et al. (2014). Concomitant Notch activation and p53 deletion trigger epithelial-to-mesenchymal transition and metastasis in mouse gut. Nat. Commun. *5*, 5005.

Cheifetz, S., Hernandez, H., Laiho, M., Ten Dijke, P., Iwata, K.K., and Massague, J. (1990). Distinct transforming growth factor-beta (TGF-beta) receptor subsets as determinants of cellular responsiveness to three TGF-beta isoforms. J. Biol. Chem. *265*, 20533–20538.

Church, D.M., Schneider, V.A., Graves, T., Auger, K., Cunningham, F., Bouk, N., Chen, H.C., Agarwala, R., McLaren, W.M., Ritchie, G.R., et al. (2011). Modernizing reference genome assemblies. PLoS Biol. *9*, e1001091.

Clevers, H., and Batlle, E. (2006). EphB/EphrinB receptors and Wnt signaling in colorectal cancer. Cancer Res. *66*, 2–5.

Dienstmann, R., Vermeulen, L., Guinney, J., Kopetz, S., Tejpar, S., and Tabernero, J. (2017). Consensus molecular subtypes and the evolution of precision medicine in colorectal cancer. Nat. Rev. Cancer *17*, 79–92.

Dunne, P.D., Alderdice, M., O'Reilly, P.G., Roddy, A.C., McCorry, A.M.B., Richman, S., Maughan, T., McDade, S.S., Johnston, P.G., Longley, D.B., et al. (2017). Cancer-cell intrinsic gene expression signatures overcome intratumoural heterogeneity bias in colorectal cancer patient classification. Nat. Commun. *8*, 15657.

Durinck, S., Spellman, P.T., Birney, E., and Huber, W. (2009). Mapping identifiers for the integration of genomic datasets with the R/Bioconductor package biomaRt. Nat. Protoc. *4*, 1184–1191.

Elyada, E., Pribluda, A., Goldstein, R.E., Morgenstern, Y., Brachya, G., Cojocaru, G., Snir-Alkalay, I., Burstain, I., Haffner-Krausz, R., Jung, S., et al. (2011). CKIalpha ablation highlights a critical role for p53 in invasiveness control. Nature *470*, 409–413.

Evrard, M., Kwok, I.W.H., Chong, S.Z., Teng, K.W.W., Becht, E., Chen, J., Sieow, J.L., Penny, H.L., Ching, G.C., Devi, S., et al. (2018). Developmental analysis of bone marrow neutrophils reveals populations specialized in expansion, trafficking, and effector functions. Immunity *48*, 364–379.e8.

Faller, W.J., Jackson, T.J., Knight, J.R., Ridgway, R.A., Jamieson, T., Karim, S.A., Jones, C., Radulescu, S., Huels, D.J., Myant, K.B., et al. (2015). mTORC1-mediated translational elongation limits intestinal tumour initiation and growth. Nature *517*, 497–500.

Fearon, E.R. (2011). Molecular genetics of colorectal cancer. Annu. Rev. Pathol. *6*, 479–507.

Fearon, E.R., and Vogelstein, B. (1990). A genetic model for colorectal tumorigenesis. Cell *61*, 759–767.

Fessler, E., Drost, J., van Hooff, S.R., Linnekamp, J.F., Wang, X., Jansen, M., de Sousa, E.M.F., Prasetyanti, P.R., Je, I.J., et al. (2016). TGFbeta signaling directs serrated adenomas to the mesenchymal colorectal cancer subtype. EMBO Mol. Med. *8*, 745–760.

Van der Flier, L.G., Sabates-Bellver, J., Oving, I., Haegebarth, A., de Palo, M., Anti, M., van Gijn, M.E., Suijkerbuijk, S., van de Wetering, M., et al. (2007). The intestinal Wnt/TCF signature. Gastroenterology *132*, 628–632.

Fre, S., Huyghe, M., Mourikis, P., Robine, S., Louvard, D., and Artavanis-Tsakonas, S. (2005). Notch signals control the fate of immature progenitor cells in the intestine. Nature *435*, 964–968.

Fre, S., Pallavi, S.K., Huyghe, M., Lae, M., Janssen, K.P., Robine, S., Artavanis-Tsakonas, S., and Louvard, D. (2009). Notch and Wnt signals cooperatively control cell proliferation and tumorigenesis in the intestine. Proc. Natl. Acad. Sci. U S A *106*, 6309–6314.

Fridlender, Z.G., Sun, J., Kim, S., Kapoor, V., Cheng, G., Ling, L., Worthen, G.S., and Albelda, S.M. (2009). Polarization of tumor-associated neutrophil phenotype by TGF-beta: "N1" versus "N2" TAN. Cancer Cell *16*, 183–194.

Guinney, J., Dienstmann, R., Wang, X., de Reynies, A., Schlicker, A., Soneson, C., Marisa, L., Roepman, P., Nyamundanda, G., Angelino, P., et al. (2015). The consensus molecular subtypes of colorectal cancer. Nat. Med. *21*, 1350–1356.

Han, T., Schatoff, E.M., Murphy, C., Zafra, M.P., Wilkinson, J.E., Elemento, O., and Dow, L.E. (2017). R-Spondin chromosome rearrangements drive Wnt-dependent tumour initiation and maintenance in the intestine. Nat. Commun. *8*, 15945.

Hasenberg, A., Hasenberg, M., Mann, L., Neumann, F., Borkenstein, L., Stecher, M., Kraus, A., Engel, D.R., Klingberg, A., Seddigh, P., et al. (2015). Catchup: a mouse model for imaging-based tracking and modulation of neutrophil granulocytes. Nat. Methods *12*, 445–452.

Hoare, M., Ito, Y., Kang, T.W., Weekes, M.P., Matheson, N.J., Patten, D.A., Shetty, S., Parry, A.J., Menon, S., Salama, R., et al. (2016). NOTCH1 mediates a switch between two distinct secretomes during senescence. Nat. Cell Biol. *18*, 979–992.

IJspeert, J.E., Vermeulen, L., Meijer, G.A., and Dekker, E. (2015). Serrated neoplasia-role in colorectal carcinogenesis and clinical implications. Nat. Rev. Gastroenterol. Hepatol. *12*, 401–409.

Irshad, S., Bansal, M., Guarnieri, P., Davis, H., Al Haj Zen, A., Baran, B., Pinna, C.M.A., Rahman, H., Biswas, S., Bardella, C., et al. (2017). Bone morphogenetic protein and Notch signalling crosstalk in poor-prognosis, mesenchymal-subtype colorectal cancer. J. Pathol. *242*, 178–192.

Isella, C., Terrasi, A., Bellomo, S.E., Petti, C., Galatola, G., Muratore, A., Mellano, A., Senetta, R., Cassenti, A., Sonetto, C., et al. (2015). Stromal contribution to the colorectal cancer transcriptome. Nat. Genet. *47*, 312–319.

Isella, C., Brundu, F., Bellomo, S.E., Galimi, F., Zanella, E., Porporato, R., Petti, C., Fiori, A., Orzan, F., Senetta, R., et al. (2017). Selective analysis of cancer-cell intrinsic transcriptional traits defines novel clinically relevant subtypes of colorectal cancer. Nat. Commun. *8*, 15107.

Jackson, E.L., Willis, N., Mercer, K., Bronson, R.T., Crowley, D., Montoya, R., Jacks, T., and Tuveson, D.A. (2001). Analysis of lung tumor initiation and progression using conditional expression of oncogenic K-ras. Genes Dev. *15*, 3243–3248.

Jackstadt, R., and Sansom, O.J. (2016). Mouse models of intestinal cancer. J. Pathol. *238*, 141–151.

Janssen, K.P., el-Marjou, F., Pinto, D., Sastre, X., Rouillard, D., Fouquet, C., Soussi, T., Louvard, D., and Robine, S. (2002). Targeted expression of oncogenic K-ras in intestinal epithelium causes spontaneous tumorigenesis in mice. Gastroenterology *123*, 492–504.

Jass, J.R., Whitehall, V.L., Young, J., and Leggett, B.A. (2002). Emerging concepts in colorectal neoplasia. Gastroenterology *123*, 862–876.

Jemal, A., Bray, F., Center, M.M., Ferlay, J., Ward, E., and Forman, D. (2011). Global cancer statistics. CA Cancer J. Clin. *61*, 69–90.

Johnson, W.E., Li, C., and Rabinovic, A. (2007). Adjusting batch effects in microarray expression data using empirical Bayes methods. Biostatistics *8*, 118–127.

Jones, D., Raine, K.M., Davies, H., Tarpey, P.S., Butler, A.P., Teague, J.W., Nik-Zainal, S., and Campbell, P.J. (2016). cgpCaVEManWrapper: simple execution of CaVEMan in order to detect somatic single nucleotide variants in NGS data. Curr. Protoc. Bioinformatics *56*, 15.10.1–15.10.18.

Jonkers, J., Meuwissen, R., van der Gulden, H., Peterse, H., van der Valk, M., and Berns, A. (2001). Synergistic tumor suppressor activity of BRCA2 and p53 in a conditional mouse model for breast cancer. Nat. Genet. *29*, 418–425.

Keane, T.M., Goodstadt, L., Danecek, P., White, M.A., Wong, K., Yalcin, B., Heger, A., Agam, A., Slater, G., Goodson, M., et al. (2011). Mouse genomic variation and its effect on phenotypes and gene regulation. Nature *477*, 289–294.

Kim, H.A., Koo, B.K., Cho, J.H., Kim, Y.Y., Seong, J., Chang, H.J., Oh, Y.M., Stange, D.E., Park, J.G., Hwang, D., and Kong, Y.Y. (2012). Notch1 counteracts WNT/beta-catenin signaling through chromatin modification in colorectal cancer. J. Clin. Invest *122*, 3248–3259.

Kim, D., Pertea, G., Trapnell, C., Pimentel, H., Kelley, R., and Salzberg, S.L. (2013). TopHat2: accurate alignment of transcriptomes in the presence of insertions, deletions and gene fusions. Genome Biol. *14*, R36.

Krepler, C., Xiao, M., Samanta, M., Vultur, A., Chen, H.Y., Brafford, P., Reyes-Uribe, P.I., Halloran, M., Chen, T., He, X., et al. (2016). Targeting Notch enhances the efficacy of ERK inhibitors in BRAF-V600E melanoma. Oncotarget *7*, 71211–71222.

Kwon, O.J., Zhang, L., Wang, J., Su, Q., Feng, Q., Zhang, X.H., Mani, S.A., Paulter, R., Creighton, C.J., Ittmann, M.M., and Xin, L. (2016). Notch promotes tumor metastasis in a prostate-specific Pten-null mouse model. J. Clin. Invest *126*, 2626–2641.

Langmead, B., and Salzberg, S.L. (2012). Fast gapped-read alignment with Bowtie 2. Nat. Methods *9*, 357–359.

Larsson, J., Goumans, M.J., Sjostrand, L.J., Van Rooijen, M.A., Ward, D., Leveen, P., Xu, X., Ten Dijke, P., Mummery, C.L., and Karlsson, S. (2001). Abnormal angiogenesis but intact hematopoietic potential in TGF-beta type I receptor-deficient mice. EMBO J. *20*, 1663–1673.

Leach, J., Morton, J.P., and Sansom, O.J. (2019). Neutrophils: homing in on the myeloid mechanisms of metastasis. Mol. Immunol. *110*, 69–76.

Leek, J.T., Johnson, W.E., Parker, H.S., Jaffe, A.E., and Storey, J.D. (2012). The sva package for removing batch effects and other unwanted variation in high-throughput experiments. Bioinformatics *28*, 882–883.

Li, H., and Durbin, R. (2009). Fast and accurate short read alignment with Burrows-Wheeler transform. Bioinformatics *25*, 1754–1760.

Liu, J., Pan, S., Hsieh, M.H., Ng, N., Sun, F., Wang, T., Kasibhatla, S., Schuller, A.G., Li, A.G., Cheng, D., et al. (2013). Targeting Wnt-driven cancer through the inhibition of Porcupine by LGK974. Proc. Natl. Acad. Sci. U S A *110*, 20224–20229.

Love, M.I., Huber, W., and Anders, S. (2014). Moderated estimation of fold change and dispersion for RNA-seq data with DESeq2. Genome Biol. *15*, 550.

Lu, J., Ye, X., Fan, F., Xia, L., Bhattacharya, R., Bellister, S., Tozzi, F., Sceusi, E., Zhou, Y., Tachibana, I., et al. (2013). Endothelial cells promote the colorectal cancer stem cell phenotype through a soluble form of Jagged-1. Cancer Cell *23*, 171–185.

el Marjou, F., Janssen, K.P., Chang, B.H., Li, M., Hindie, V., Chan, L., Louvard, D., Chambon, P., Metzger, D., and Robine, S. (2004). Tissue-specific and inducible Cre-mediated recombination in the gut epithelium. Genesis *39*, 186–193.

McCorry, A.M., Loughrey, M.B., Longley, D.B., Lawler, M., and Dunne, P.D. (2018). Epithelial-to-mesenchymal transition signature assessment in colorectal cancer quantifies tumour stromal content rather than true transition. J. Pathol. *246*, 422–426.

Meurette, O., and Mehlen, P. (2018). Notch signaling in the tumor microenvironment. Cancer Cell *34*, 536–548.

Murtaugh, L.C., Stanger, B.Z., Kwan, K.M., and Melton, D.A. (2003). Notch signaling controls multiple steps of pancreatic differentiation. Proc. Natl. Acad. Sci. U S A *100*, 14920–14925.

Nicholls, D.J., Wiley, K., Dainty, I., Macintosh, F., Phillips, C., Gaw, A., and Mardh, C.K. (2015). Pharmacological characterization of AZD5069, a slowly reversible CXC chemokine receptor 2 antagonist. J. Pharmacol. Exp. Ther. *353*, 340–350.

Noah, T.K., and Shroyer, N.F. (2013). Notch in the intestine: regulation of homeostasis and pathogenesis. Annu. Rev. Physiol. *75*, 263–288.

O'Rourke, K.P., Loizou, E., Livshits, G., Schatoff, E.M., Baslan, T., Manchado, E., Simon, J., Romesser, P.B., Leach, B., Han, T., et al. (2017). Transplantation of engineered organoids enables rapid generation of metastatic mouse models of colorectal cancer. Nat. Biotechnol. *35*, 577–582.

Olive, K.P., Tuveson, D.A., Ruhe, Z.C., Yin, B., Willis, N.A., Bronson, R.T., Crowley, D., and Jacks, T. (2004). Mutant p53 gain of function in two mouse models of Li-Fraumeni syndrome. Cell *119*, 847–860.

Plasari, G., Calabrese, A., Dusserre, Y., Gronostajski, R.M., McNair, A., Michalik, L., and Mermod, N. (2009). Nuclear factor I-C links platelet-derived growth factor and transforming growth factor beta1 signaling to skin wound healing progression. Mol. Cell. Biol. *29*, 6006–6017.

Pribluda, A., Elyada, E., Wiener, Z., Hamza, H., Goldstein, R.E., Biton, M., Burstain, I., Morgenstern, Y., Brachya, G., Billauer, H., et al. (2013). A senescence-inflammatory switch from cancer-inhibitory to cancer-promoting mechanism. Cancer Cell *24*, 242–256.

Ritchie, M.E., Phipson, B., Wu, D., Hu, Y., Law, C.W., Shi, W., and Smyth, G.K. (2015). limma powers differential expression analyses for RNA-sequencing and microarray studies. Nucleic Acids Res. *43*, e47.

Rodilla, V., Villanueva, A., Obrador-Hevia, A., Robert-Moreno, A., Fernandez-Majada, V., Grilli, A., Lopez-Bigas, N., Bellora, N., Alba, M.M., Torres, F., et al. (2009). Jagged1 is the pathological link between Wnt and Notch pathways in colorectal cancer. Proc. Natl. Acad. Sci. U S A *106*, 6315–6320.

Romano, G., Chagani, S., and Kwong, L.N. (2018). The path to metastatic mouse models of colorectal cancer. Oncogene *37*, 2481–2489.

Roper, J., Tammela, T., Cetinbas, N.M., Akkad, A., Roghanian, A., Rickelt, S., Almeqdadi, M., Wu, K., Oberli, M.A., Sanchez-Rivera, F., et al. (2017). In vivo genome editing and organoid transplantation models of colorectal cancer and metastasis. Nat. Biotechnol. *35*, 569–576.

Roxburgh, C.S., Wallace, A.M., Guthrie, G.K., Horgan, P.G., and McMillan, D.C. (2010). Comparison of the prognostic value of tumour- and patient-related factors in patients undergoing potentially curative surgery for colon cancer. Colorectal Dis. *12*, 987–994.

Sakai, E., Nakayama, M., Oshima, H., Kouyama, Y., Niida, A., Fujii, S., Ochiai, A., Nakayama, K.I., Mimori, K., Suzuki, Y., et al. (2018). Combined mutation of Apc, Kras, and Tgfbr2 effectively drives metastasis of intestinal cancer. Cancer Res. *78*, 1334–1346.

Sancho, R., Cremona, C.A., and Behrens, A. (2015). Stem cell and progenitor fate in the mammalian intestine: notch and lateral inhibition in homeostasis and disease. EMBO Rep. *16*, 571–581.

Sato, T., Vries, R.G., Snippert, H.J., van de Wetering, M., Barker, N., Stange, D.E., Van Es, J.H., Abo, A., Kujala, P., Peters, P.J., and Clevers, H. (2009). Single Lgr5 stem cells build crypt-villus structures in vitro without a mesenchymal niche. Nature *459*, 262–265.

Schmidt, E.M., Lamprecht, S., Blaj, C., Schaaf, C., Krebs, S., Blum, H., Hermeking, H., Jung, A., Kirchner, T., and Horst, D. (2018). Targeting tumor cell plasticity by combined inhibition of NOTCH and MAPK signaling in colon cancer. J. Exp. Med. *215*, 1693–1708.

Seshagiri, S., Stawiski, E.W., Durinck, S., Modrusan, Z., Storm, E.E., Conboy, C.B., Chaudhuri, S., Guan, Y., Janakiraman, V., Jaiswal, B.S., et al. (2012). Recurrent R-spondin fusions in colon cancer. Nature *488*, 660–664.

Shen, Q., Cohen, B., Zheng, W., Rahbar, R., Martin, B., Murakami, K., Lamorte, S., Thompson, P., Berman, H., Zuniga-Pflucker, J.C., et al. (2017). Notch shapes the innate immunophenotype in breast cancer. Cancer Discov. *7*, 1320–1335.

Shibata, H., Toyama, K., Shioya, H., Ito, M., Hirota, M., Hasegawa, S., Matsumoto, H., Takano, H., Akiyama, T., Toyoshima, K., et al. (1997). Rapid colorectal adenoma formation initiated by conditional targeting of the Apc gene. Science *278*, 120–123.

Smedley, D., Haider, S., Durinck, S., Pandini, L., Provero, P., Allen, J., Arnaiz, O., Awedh, M.H., Baldock, R., Barbiera, G., et al. (2015). The BioMart community portal: an innovative alternative to large, centralized data repositories. Nucleic Acids Res. *43*, W589–W598.

Sonoshita, M., Aoki, M., Fuwa, H., Aoki, K., Hosogi, H., Sakai, Y., Hashida, H., Takabayashi, A., Sasaki, M., Robine, S., et al. (2011). Suppression of colon cancer metastasis by Aes through inhibition of Notch signaling. Cancer Cell *19*, 125–137.

Sonoshita, M., Itatani, Y., Kakizaki, F., Sakimura, K., Terashima, T., Katsuyama, Y., Sakai, Y., and Taketo, M.M. (2015). Promotion of colorectal cancer invasion and metastasis through activation of NOTCH-DAB1-ABL-RHOGEF protein TRIO. Cancer Discov. *5*, 198–211.

Sottoriva, A., Kang, H., Ma, Z., Graham, T.A., Salomon, M.P., Zhao, J., Marjoram, P., Siegmund, K., Press, M.F., Shibata, D., and Curtis, C. (2015). A Big Bang model of human colorectal tumor growth. Nat. Genet. *47*, 209–216.

de Sousa e Melo, F., Kurtova, A.V., Harnoss, J.M., Kljavin, N., Hoeck, J.D., Hung, J., Anderson, J.E., Storm, E.E., Modrusan, Z., Koeppen, H., et al.

(2017). A distinct role for Lgr5(+) stem cells in primary and metastatic colon cancer. Nature *543*, 676–680.

De Sousa, E.M.F., Wang, X., Jansen, M., Fessler, E., Trinh, A., de Rooij, L.P., de Jong, J.H., de Boer, O.J., van Leersum, R., Bijlsma, M.F., et al. (2013). Poor-prognosis colon cancer is defined by a molecularly distinct subtype and develops from serrated precursor lesions. Nat. Med. *19*, 614–618.

Steele, C.W., Karim, S.A., Leach, J.D., Bailey, P., Upstill-Goddard, R., Rishi, L., Foth, M., Bryson, S., McDaid, K., Wilson, Z., et al. (2016). CXCR2 inhibition profoundly suppresses metastases and augments immunotherapy in pancreatic ductal adenocarcinoma. Cancer Cell *29*, 832–845.

Storm, E.E., Durinck, S., de Sousa e Melo, F., Tremayne, J., Kljavin, N., Tan, C., Ye, X., Chiu, C., Pham, T., Hongo, J.A., et al. (2016). Targeting PTPRK-RSPO3 colon tumours promotes differentiation and loss of stem-cell function. Nature *529*, 97–100.

Tauriello, D.V., Calon, A., Lonardo, E., and Batlle, E. (2017). Determinants of metastatic competency in colorectal cancer. Mol. Oncol. *11*, 97–119.

Tauriello, D.V.F., Palomo-Ponce, S., Stork, D., Berenguer-Llergo, A., Badia-Ramentol, J., Iglesias, M., Sevillano, M., Ibiza, S., Canellas, A., Hernando-Momblona, X., et al. (2018). TGFbeta drives immune evasion in genetically reconstituted colon cancer metastasis. Nature *554*, 538–543.

Trobridge, P., Knoblaugh, S., Washington, M.K., Munoz, N.M., Tsuchiya, K.D., Rojas, A., Song, X., Ulrich, C.M., Sasazuki, T., Shirasawa, S., and Grady, W.M. (2009). TGF-beta receptor inactivation and mutant Kras induce intestinal neoplasms in mice via a beta-catenin-independent pathway. Gastroenterology *136*, 1680–1688.e7.

Wellenstein, M.D., and de Visser, K.E. (2018). Cancer-cell-intrinsic mechanisms shaping the tumor immune landscape. Immunity *48*, 399–416.

Wieland, E., Rodriguez-Vita, J., Liebler, S.S., Mogler, C., Moll, I., Herberich, S.E., Espinet, E., Herpel, E., Menuchin, A., Chang-Claude, J., et al. (2017). Endothelial Notch1 activity facilitates metastasis. Cancer Cell *31* (3), 355–367.

Yamashita, A.S., Geraldo, M.V., Fuziwara, C.S., Kulcsar, M.A., Friguglietti, C.U., da Costa, R.B., Baia, G.S., and Kimura, E.T. (2013). Notch pathway is activated by MAPK signaling and influences papillary thyroid cancer proliferation. Transl Oncol. *6*, 197–205.

Yan, H.H.N., Lai, J.C.W., Ho, S.L., Leung, W.K., Law, W.L., Lee, J.F.Y., Chan, A.K.W., Tsui, W.Y., Chan, A.S.Y., Lee, B.C.H., et al. (2017). RNF43 germline and somatic mutation in serrated neoplasia pathway and its association with BRAF mutation. Gut *66*, 1645–1656.

Ye, K., Schulz, M.H., Long, Q., Apweiler, R., and Ning, Z. (2009). Pindel: a pattern growth approach to detect break points of large deletions and medium sized insertions from paired-end short reads. Bioinformatics *25*, 2865–2871.

STAR★METHODS

KEY RESOURCES TABLE

REAGENT or RESOURCE	SOURCE	IDENTIFIER
Antibodies		
TruStain FcX™ (anti-mouse CD16/32) Antibody	Biolegend	101320; RRID: AB_1574975
CD45 Monoclonal Antibody (30-F11), Super Bright 600	ThermoFisher	63-0451-82; RRID: AB_2637149
PE/Cy7 anti-mouse CD48 Antibody	Biolegend	103424; RRID: AB_2075049
Brilliant Violet 785™ anti-mouse/human CD11b Antibody	Biolegend	101243; RRID: AB_2561373
BUV395 Rat Anti-Mouse Ly-6G	BD Biosciences	563978; RRID: AB_2716852
CD101 Monoclonal Antibody (Moushi101), PE	ThermoFisher	12-1011-82; RRID: AB_1210728
FITC anti-mouse CD4 Antibody	Biolegend	100510; RRID: AB_312713
Brilliant Violet 421™ anti-mouse CD3 Antibody	Biolegend	100228; RRID: AB_2562553
Alexa Fluor® 700 anti-mouse CD8a Antibody	Biolegend	100730; RRID: AB_493703
Alexa Fluor® 647 anti-human/mouse Granzyme B Antibody	Biolegend	515406; RRID: AB_2566333
IFN gamma Monoclonal Antibody (XMG1.2), PE-Cyanine7	ThermoFisher	25-7311-41; RRID: AB_1257211
PE anti-mouse CD69 Antibody	Biolegend	104508; RRID: AB_313111
Anti-CD3 antibody [SP7]	Abcam	Ab16669; RRID: AB_443425
CD4 Monoclonal Antibody (4SM95)	eBioscience	14-9766-82; RRID: AB_2573008
CD8a Monoclonal Antibody (4SM15)	eBioscience	14-0808-82; RRID: AB_2572861
Calgranulin B Antibody (M-19)	Santa Cruz	sc-8115; RRID: AB_2269986
Purified Rat Anti-Mouse CD44 Clone IM7	BD Biosciences	550538; RRID: AB_393732
Anti-Sox9 Antibody	Millipore	AB5535; RRID: AB_2239761
Purified Mouse Anti-β-Catenin Clone 14	BD Biosciences	610154; RRID: AB_397555
Anti-MLH1 antibody [EPR3894]	Abcam	ab92312; RRID: AB_2049968
Anti-CALD1 antibody produced in rabbit	Sigma-Aldrich	HPA008066; RRID: AB_1078378
Anti-IGFBP7 antibody produced in rabbit	Sigma-Aldrich	HPA002196; RRID: AB_1079107
Anti-Smad3 (phospho S423 + S425) antibody	Abcam	Ab52903; RRID: AB_882596
Monoclonal Anti-Actin, α-Smooth Muscle	Sigma-Aldrich	A2547; RRID: AB_476701
4.3.11.3 mouse MAb	Hypoxyprobe	HP1-100; RRID: AB_2801307
InVivoMAb anti-mouse Ly6G	Bioxcell	BE0075-1; RRID: AB_1107721
InVivoMAb Rat IgG2a isotype control; clone 2A3	Bioxcell	BE0089; RRID: AB_1107769
InVivoMAb TGF-β ligand-antibody; clone 1D11	Bioxcell	BE0057; RRID: AB_1107757
InVivoMAb TGF-β ligand-antibody isotype control; clone MOPC-21	Bioxcell	BE0083; RRID: AB_1107784
CXCR2 Polyclonal Antibody	ThermoFisher	PA1-20673; RRID: AB_2126489
Myeloperoxidase (MPO)	Dako	A0398; RRID: AB_2335676
Cleaved Notch1 (Val1744) (D3B8) Rabbit mAb	Cell Signaling Technology	4147; RRID: AB_2153348
RBPSUH (D10A4) XP® Rabbit mAb	Cell Signaling Technology	5313; RRID: AB_2665555
Normal Rabbit IgG	Cell Signaling Technology	2729; RRID: AB_1031062
Chemicals, Peptides, and Recombinant Proteins		
CXCR2 inhibitor; AZD5069	AstraZeneca	N/A
Alk5 inhibitor; AZ12601011	AstraZeneca	N/A
LGK974	Active Biochem	A-1400
Permeabilization Buffer (10X)	Invitrogen	00-8333-56
ArC™ Amine Reactive Compensation Bead Kit	ThermoFisher	A10346
UltraComp eBeads™ Compensation Beads	ThermoFisher	01-2222-41
GentleMACS C Tubes	Miltenyi Biotec	130-093-237
Mouse Tumor Dissociation Kit	Miltenyi Biotec	130-096-730
LIVE/DEAD™ Fixable Near-IR Dead Cell Stain Kit	ThermoFisher	L10119

(*Continued on next page*)

Continued

REAGENT or RESOURCE	SOURCE	IDENTIFIER
Critical Commercial Assays		
SimpleChIP® Plus Enzymatic Chromatin IP Kit	Cell Signaling Technology	9005
Deposited Data		
Whole genome sequencing data	This paper	European Nucleotide Archive ID: ERP040713
Raw RNA-sequencing data	This paper	ArrayExpress ID: E-MTAB-6363
Experimental Models: Cell Lines		
*villin*Cre^{ER}; *Kras*$^{G12D/+}$; *Trp53*$^{fl/fl}$ *Rosa26*$^{N1icd/+}$ organoids	This paper	N/A
*villin*Cre^{ER}; *Apc*$^{fl/fl}$; *Kras*$^{G12D/}$+; *Trp53*$^{fl/fl}$ *Tgfbrl*$^{fl/fl}$ organoids	This paper	N/A
Experimental Models: Organisms/Strains		
villinCreER, Tg(Vil-cre/ERT2)23Syr	(el Marjou et al., 2004)	N/A
Ly6GCre, Ly6gtm2621(Cre-tdTomato)Arte	(Hasenberg et al., 2015)	N/A
Apc (floxed), Apctm1Tno	(Shibata et al., 1997)	N/A
Rosa26-Notch1 ICD, Gt(ROSA)26Sortm1(Notch1)Dam	(Murtaugh et al., 2003)	N/A
p53 (floxed), Trp53tm1Brn	(Jonkers et al., 2001)	N/A
p53 (R172H), Trp53tm2Tyj	(Olive et al., 2004)	N/A
Kras (G12D); Krastm4Tyj	(Jackson et al., 2001)	N/A
Alk5 (floxed), tm1Karl	(Larsson et al., 2001)	N/A
CD-1 Nude; CD1-*Foxn1*^{nu}	Charles River UK	Strain code 086
C57BL/6	Charles River UK	Strain code 632
Oligonucleotides		
ChIP and qPCR	This paper	Table S8
Software and Algorithms		
GraphPad Prism software v7.03	GraphPad Software	N/A
FlowJo v10.4.2.	FlowJo	N/A
HALO Image analysis software	Indica Labs	V2.0.1145
Other		
BD LSRFortessa	BD Biosciences	N/A
IDEXX ProCyte Dx	IDEXX	N/A
SCN400F slide scanner	Leica Microsystems	N/A
Bond Rx autostainer	Leica	N/A
GentleMACS Octo Dissociator with Heaters	Miltenyi Biotec	130-096-427
7500 Fast Real-Time PCR System	Applied Biosystems	N/A
AutostainerLink 48	Dako	N/A

CONTACT FOR REAGENT AND RESOURCE SHARING

Requests for further information, reagents, and resources should be directed to and will be fulfilled by the Lead Contact, Owen J. Sansom: (o.sansom@beatson.gla.ac.uk).

EXPERIMENTAL MODEL AND SUBJECT DETAILS

Animals

Species used: Mus musculus

Tumor Models and Treatments

All animal experiments were performed in accordance with a UK Home Office licence (Project License 70/8646), adhered to ARRIVE guidelines and were subject to review by the animal welfare and ethical review board of the University of Glasgow. Both genders were induced with a single injection of 2 mg tamoxifen (Sigma-Aldrich, T5648) by intraperitoneal injection at an age of 6 to 12 weeks, all experiments were performed on a C57BL/6 background (N = 5 or more). Mice were sampled at clinical endpoint, which was defined

as weight loss and/or hunching and/or cachexia. Mice were censored $\geq$ 550 days after tamoxifen administration or if sampled not due to intestinal tumor burden or associated metastasis. The alleles used can be found in the Key Resources Table.

Alk5 inhibitor (Alk5i) (Anderton et al., 2011) (AstraZeneca, AZ12601011) was administered at 50 mg/kg and CXCR2 small molecule (AstraZeneca, AZD5069) at 100 mg/kg, both in 0.5% Hydroxypropyl Methylcellulose (HPMC) and 0.1% Tween-80 twice daily by oral gavage. As vehicle control for Alk5i and CXCR2sm 0.5% HPMC and 0.1% Tween-80 was given with the same regime. Ly6G-antibody (clone 1A8, BioXcell, BE0075-1) or isotype control (clone 2A3, BioXcell, BE0089) were administered three times a week by intraperitoneal injection at 10 mg/kg. TGF-β ligand-antibody (clone 1D11, BioXcell, BE0057) or isotype control (clone MOPC-21, BioXcell, BE0083) were administered three times a week by intraperitoneal injection at 5 mg/kg. LGK974 (Active Biochem, A-1400) was administered at 5 mg/kg, in 0.5% Methylcellulose (MC) and 0.5% Tween-80 twice daily by oral gavage. As vehicle control 0.5% MC and 0.5% Tween-80 was given with the same regime. Treatments were started 85 or 130 days after initial tamoxifen injection; short term treatments of tumor bearing mice were started when tumors were palpable.

Patient Material

46 patients who underwent synchronous resection of colorectal primary tumor and liver metastases between April 2002 and June 2010 at Glasgow Royal Infirmary were included in the study, details can be found in Table S6. Patients were identified from a prospectively maintained database and represent a consecutive cohort of resected patients. Application to access patient tissue was approved by the NHS Greater Glasgow and Clyde biorepository and ethical approval granted in biorepository application #357 and informed consent was obtained from all subjects. Patients were followed up at one month, six monthly until two years, and thereafter annually until five years at which point they were discharged. Recurrence data, morbidity, and mortality was prospectively collected. Information on date and cause of death was determined via access to the NHS Greater Glasgow and Clyde clinical portal. Death records were complete until 1st November 2017, which served as the censor date.

Human liver metastases were anonymised, five micron-thick, formalin fixed and paraffin embedded sections of liver containing metastatic colorectal carcinoma from partial hepatectomy specimens were stained for N1ICD. The use of the human material was approved by the Lothian NRS Human Annotated Bioresource and informed consent was obtained from all subjects (ethical review number 15/ES/0094).

METHOD DETAILS

Scoring of Tumor Stage and Differentiation

T staging of tumors was performed by a boarded pathologist according to the following parameters included in the classical TNM classification; T0, no evidence of primary tumor. Tis, Carcinoma *in situ*: intraepithelial or invasion of the lamina propria (i.e. no extension through the muscularis mucosae and therefore the submucosa). T1, Tumor invades submucosa. T2, Tumor invades muscularis propria. T3, Tumor invades into the subserosa. T4, Tumor invades/perforates the visceral peritoneum and into other adjacent organs/structures.

Tumor differentiation scoring was performed by a boarded pathologist according to the following parameters; well differentiated tumors exhibit clear glandular differentiation in >95% of the tumor. Moderately differentiated tumors exhibit glandular differentiation in 50-95% of the tumor. Poorly differentiated carcinomas exhibit glandular differentiation in 5-50% of the tumor.

Blood Count Analysis

Blood was collected in EDTA columns after cardiac puncture. Blood samples were analyzed with IDEXX ProCyte Dx.

Organoid Culture

Advanced DMEM/F12 was supplemented with penicillin/streptomycin (100 U/ml / 100 μg/ml) (15140122), 2 mM L-Glutamine (25030081), 10 mM HEPES (15630080), N2-supplement (17502001) and B27-supplement (17504044) (all ordered from Gibco, Life Technologies or ThermoFisher-Scientific) and from here on is referred to as ADF. Complete ADF was prepared by supplementing ADF with 50 ng/ml Recombinant Human EGF (Peprotech, AF-100-15), 100 ng/ml Recombinant Murine Noggin (Peprotech, 250-38) and 500 ng/ml Recombinant Mouse R-spondin-1 (R&D systems, 3474-RS). Intestinal epithelium extraction (Faller et al., 2015) and culture conditions were previously described (Sato et al., 2009). These culture conditions were used unless stated differently in figure legends.

Tumors were cut into small fragments and washed five times in PBS. Tumor fragments were incubated in 5 ml 10x Trypsin (5mg/ml, Gibco), 1x DNase buffer and 200U recombinant DNase I (Roche, 04716728001) at 37°C for 30 minutes. To further dissociate tumor fragments, 5 ml ADF was added and tumor fragments were shaken vigorously. This step was repeated five times. After aspirating the supernatant and re-suspending the pellet in 10 ml ADF, the suspension was passed through a 70 μm cell strainer. The cell pellet was re-suspended in Matrigel (BD Bioscience, 356231) according to pellet volume and seeded. Organoids/spheroids were cultured in complete ADF at 37°C, 5% CO_2, 21% O_2.

Single Cell Seeding

Organoids were harvested and dissociated by fiercely pipetting. Organoids were washed twice with PBS before being dissociated into single cells by incubating in 2 ml 10x Trypsin (5mg/ml), 1x DNase buffer and 200U recombinant DNase I (Roche, 04716728001) at

37°C for 7 minutes. Cells were passed through a 40 μm cell strainer before 1000 single cells were seeded in 20 μl Matrigel in a 24-wells culture plate. LGK974 10 μM (Active Biochem, A-1400) and appropriate volumes of vehicle (DMSO) were added at the moment of single cell seeding. Organoid forming capacity was assessed after one week by measurement of diameter and counting the number of organoids formed in each culture condition.

Intra-Splenic Injection of Organoids

To prepare the cell suspension, KPN (liver metastases derived; C57BL/6, N=10) or *villin*Cre^{ER} *Apc*$^{fl/fl}$ *Kras*$^{G12D/+}$ *Trp53*$^{fl/fl}$ *Trgfbrl*$^{fl/fl}$ (AKPT; small intestinal derived; C57BL/6, N=7) organoids were cultured in conditions as described above without R-spondin. Tumor cells were harvested and washed with PBS and trypsinized with 0.25% Trypsin in PBS-EDTA for 7 minutes at 37°C. After trypsinization, cells were washed and passed through a 40 μm cell strainer and counted using a haemocytometer.

C57BL/6 or CD-1/Nude mice (6-12 weeks old males; Charles River, UK) were anesthetized with isoflurane, and a laparotomy was performed to gain access to the spleen. 5x10^5 single cells in 50 μL PBS were injected into the spleen after which the incision was sutured. The mice were sampled four weeks post transplantation. Organoid donor and recipient mice were sex matched.

Needle-Guided Intracolonic Organoid Transplantation

Colonic sub-mucosal injections of organoids were performed as previously described (Roper et al., 2017), using a Karl Storz TELE PACK VET X LED endoscopic video unit. KPN liver metastases derived (C57BL/6, N=10) organoids, cultured in conditions as described above without R-spondin, were harvested and dissociated by fiercely pipetting. Organoids were washed twice with PBS before being injected. Approximately 500 organoids in 70 μl PBS were injected in a single injection. At clinical end point tumors and metastasis were quantified.

Sample Processing and Staining for Flow Cytometry

Tumor samples were dissected and digested using the Mouse Tumor Dissociation Kit (Miltenyi Biotec, 130-096-730) and the GentleMACS Octo Dissociator with Heaters (Miltenyi Biotec, 130-096-427), using the 37C_m_TDK_1 programme. The cells were passed through a 70 μm cell strainer and then counted. Two million cells were stained with LIVE/DEAD fixable near-IR stain kit (ThermoFisher, L10119) at 1:1000 dilution in 100 μl PBS in the dark for 20 minutes at 4°C, then washed with PBS 1% BSA. TruStain FcX anti-mouse CD16/32 (Biolegend, 101320) was used at 1:200 in 50 μl PBS 1% BSA to block CD16/32 activity, in the dark for 15 minutes at 4°C. After that incubation time, 50 μl of the antibody staining mixes were added: for the neutrophil panel, CD45 (ThermoFisher, 63-0451-82), CD48 (Biolegend, 103424), CD11b (Biolegend, 101243), Ly6G (BD Biosciences, 563978), and CD101 (ThermoFisher, 12-1011-82). For the T cell panel, CD45 (ThermoFisher, 63-0451-82), CD3 (Biolegend, 100228), CD4 (Biolegend, 100510), CD8a (Biolegend, 100730), and CD69 (Biolegend, 104508). The cells were incubated in the dark for 30 minutes at 4°C. The cells were then washed with PBS and re-suspended in 50 μl PBS. To fix the cells, 50 μl of PBS 4% paraformaldehyde were added to the re-suspended cells, followed by incubation at room temperature in the dark for 15 minutes. The cells were washed and re-suspended in PBS. Only for the T cell panel, the cells were re-suspended in Permeabilization Buffer (ThermoFisher, 00-8333-56) instead. The cells were stained intracellularly in 1x Permeabilization Buffer with IFNγ (ThermoFisher, 25-7311-41), and Granzyme B (Biolegend, 515406) in the dark for 30 minutes at 4°C. The cells were washed in Permeabilization Buffer. Finally, the cells were washed in PBS and re-suspended in PBS for flow cytometry acquisition. Both neutrophil and T cell populations were identified with the following initial gating strategy: doublet discrimination by discrepancy between FSC-A and FSC-H signals; live cells: CD45$^+$. Subsequently, for neutrophils: CD48$^-$/^{lo}Ly6G$^+$, CD11b$^+$Ly6G$^+$ to confirm the neutrophil identity, and CD101 to characterize the neutrophils. Alternatively, for T cells: CD3$^+$, and CD4$^+$ and CD8$^+$ subsets. For both subsets, CD69 was used to analyse for activation. For the CD4 subset, IFNγ was used as an additional activation marker, while Granzyme B was used for the CD8$^+$ subset. The data were acquired with the BD LSR Fortessa flow cytometer (BD Biosciences) and analyzed with FlowJov10.4.2.

RNA Isolation

RNA was isolated using the Qiagen RNeasy Mini kit (Qiagen, 74104) according to the manufacturer's protocol including the optional DNA degradation step using the Qiagen RNase-Free DNase kit (Qiagen, 79254). Cell pellets or tissue were lysed using the Precellys lysing kit (Bertin Instruments, KT03961-1-003-2) in a Precellys Evolution machine (Bertin Instruments).

Organoids of the respective genotype, at comparable passage ~ 5 were sampled 72 hours post seeding. Organoid pellets were snap-frozen and RNA was isolated as described above.

RNA of whole tumor samples was isolated at endpoint from genotypes as indicated in figure legends and conserved in RNAlater (Sigma, R0901) at -80°C until further use for RNA isolation as described above. For sequencing tumor fragments were excised from the tumor centre to minimize effects of intra-tumor heterogeneity. Primary tumors from KPN (*villin*Cre^{ER} *Kras*$^{G12D/+}$ *Trp53*$^{fl/fl}$ *R26*$^{N1icd/+}$; small intestine), KP (*villin*Cre^{ER} *Kras*$^{G12D/+}$ *Trp53*$^{fl/fl}$; small intestine), APN (*villinCre*^{ER} *Apc*$^{fl/+}$ *Trp53*$^{fl/fl}$ *Rosa26*$^{N1icd/+}$; small intestine) and AP (*villinCre*^{ER} *Apc*$^{fl/+}$ *Trp53*$^{fl/fl}$; small intestine) tumors were sampled without exclusion of submucosa or muscularis propria.

qRT-PCR

cDNA was generated by reverse transcription of the isolated RNA using the M-MuLV-Reverse Transcriptase kit (ThermoFisher-Scientific, 28025013) according to the manufacturer's protocol. qPCR was performed using the DyNAmo HS SYBR Green qPCR kit

(ThermoFisher-Scientific, F410) according to the manufacturer's protocol. CT-values were normalized to β-Actin (*Actb*) CT-values. mRNA expression levels were calculated according to the ΔCT method and expressed as 2^(-ΔCT). Primers sequences can be found in Table S8.

Chromatin Immunoprecipitation

For chromatin immunoprecipitation (ChIP) the SimpleChIP® Plus Enzymatic Chromatin IP Kit (Cell Signaling Technology, 9005) protocol was used according to the manufacturer's instructions. In brief, KPN organoids were grown in medium conditions as described above. Cells were cross-linked for 10 minutes at room temperature and chromatin was fragmented by micrococcus nucleases followed by three sonication cycles to generate DNA fragments. Incubation with RBPSUH (D10A4) XP® Rabbit mAb (Cell Signaling Technology, 5313) or recommended concentration of rabbit normal IgG control (Cell Signaling Technology, 2729) for 16 hours at 4°C was performed. The sequences of oligonucleotides used as qChIP primers are listed in Table S8.

Immunohistochemistry

Tissues were fixed in 10% neutral buffered formalin and processed by standard histology processing techniques. The following antibodies were used: CD3 (AbCam Ab16669, pH6 1:50), CD4 (eBioscience 14-9766-82, ER2 Leica, 1:500), CD8 (eBioscience 14-0808-82, ER2 Leica,1:500), S100A9 (Santa Cruz sc-8115, pH6 1:1000), Ly6G (clone 1A8, 2B Scientific BE0075-1, ER2 Leica, 1:60000), CD44 (BD Biosciences 550538, pH6, 1:250), SOX9 (Millipore AB5535, pH6, 1:500), β-catenin (BD Biosciences 610154, pH8, 1:50), MLH11 (Abcam ab92312, pH6, 1:200), CALD1 (Sigma HPA008066, ER2 Leica, 1:400), IGFBP7 (Sigma HPA002196, ER2 Leica, 1:100), pSMAD3 (Abcam Ab52903, pH6, 1:40), αSMA (Sigma-Aldrich A2547, pH6, 1:25000), N1ICD (D3B8 Cell Signaling Technology 4147, Protaqs IX, BioCyc, 401603692, 1:50).

To stain collagen or fibrin presence within tissue sections Picro Sirius Red staining technique was used. Briefly, de-waxed slides were immersed in Picro Sirius Red solution for 2 hours. Picro Sirius Red Solution: 0.1% Direct red 80 (Sigma, 41496LH) in distilled water and 0.1% Fast green FCF (Raymond Lamb, S142-2) in distilled water were mixed in equal volumes and then diluted 1:9 with Aqueous Picric acid solution. Post staining slides were dehydrated according to standard protocols and mounted for analysis.

Hypoxia was detected by administration of Hypoxyprobe (Hypoxyprobe HP1-100; 100 μl intraperitoneal) 1 hour before sampling and detected using Hypoxyprobe recognizing antibody (Hypoxyprobe HP1-100, pH6, 1:150).

In Situ Hybridisation

In situ hybridisation (*ISH*) analysis was performed using the RNAscope 2.5 LS (Brown, 322100) detection kit (Advanced Cell Diagnostics, Hayward, CA) on a Leica Bond Rx autostainer strictly according to the manufacturer's instructions. Staining was performed on 4 μm formalin fixed paraffin sections which were cut and then placed in a 60°C oven for 2 hours prior to staining. To ensure the quality and integrity of the available RNA the tissue being investigated was tested with the positive control probe (mm-Ppib, 313918). Only after probe quality control were the results evaluated. To further ensure accuracy and integrity of the staining a negative control probe (mm-DapB, 312038) was used to confirm that the tissue staining seen was accurate due to binding with the target probe and not non-specific. Probes: *Cxcl1* (407728), *Cxcl2* (437588), *Cxcl3* (492758), *Cxcl5* (467448), *Cxcr2* (487678), *Axin2* (400338), *Lgr5* (312178), *c-Myc* (413458), *Smad7* (429418), *Tgfb1* (407758), *Tgfb2* (406188), positive control probe *Ppib* (313918) and negative control probe *DapB* (312038).

RNA-Sequencing

The quality of the purified RNA was tested on an Agilent 2200 Tapestation using RNA screen tape. Libraries for cluster generation and DNA sequencing were prepared following an adapted method from the Illumina TruSeq RNA LT Kit. Quality and quantity of the DNA libraries was assessed on a Agilent 2200 Tapestation (D1000 screentape) and Qubit (Thermo Fisher Scientific) respectively. The libraries were run on the Illumina Next Seq 500 using the High Output 75 cycles kit (2x36cycles, paired end reads, single index). Quality checks on the raw RNA-Seq data files were done using fastqc version 0.11.2 and fastq_screen version 0.11.3. RNA-seq paired-end reads were aligned to the GRCh38 (Church et al., 2011) version of the mouse genome using tophat2 version 2.0.13 (Kim et al., 2013) with Bowtie version 2.2.4.0 (Langmead and Salzberg, 2012). Expression levels were determined and statistically analyzed by a combination of HTSeq version 0.6.1, the R environment, version 3.2.2, utilizing packages from the Bioconductor data analysis suite and differential gene expression analysis based on the negative binomial distribution using the DESeq2 (Anders and Huber, 2010). All RNA-sequencing data have been deposited in the ArrayExpress database under accession number E-MTAB-6363.

Tumor and Metastasis Scoring

Macroscopic intestinal tumor or metastases were analyzed for size and number and tumor burden or metastases burden was calculated as number of tumors times tumor size. All metastases were confirmed histologically.

Image Analysis

IHC and *ISH* (RNA-scope) images were digitalized using a SCN400F slide scanner (Leica Microsystems, Milton Keynes, UK) at 20x (IHC) or 40x (*ISH*) resolution. Scanned images were analyzed using HALO Image analysis software (V2.0.1145, Indica Labs). Tumors

were analyzed for the percentage of positive cells for N1ICD, CD3, CD4, CD8a, Ly6G and S100A9. Tumor areas were manually defined using the HALO software and scoring was performed in a blinded manner for all samples. β-catenin staining was analyzed manually and considered as positive when > 10% of the tumor area was strongly positive for nuclear β-catenin.

For *Cxcl5/Cxcr2* co-analysis serial sections (3.5 μm sections) were stained for *Cxcl5* and *Cxcr2* and scanned at 40x magnification. Slides were then automatically aligned utilising the image registration module within the HALO package. Sequential, non-overlapping, paired fields of view were then individually scored.

CRC Patient Data for *In Silico* Analysis

CRC patient data were obtained from different public sources. Expression data and clinical/genetic annotation from the TCGA project (Cancer Genome Atlas Network, 2012) were downloaded from the FIREHOSE repository (https://gdac.broadinstitute.org/). This included RNA-seq expression data generated by the Illumina HiSeq (n=326) and Genome Analyzer (n=172) platforms (RSEM normalized data). After log transformation, data from both platforms were combined into a single dataset (n=498), by correcting platform-specific effects with the ComBat algorithm (Johnson et al., 2007) as implemented in the sva R package (Leek et al., 2012). From the NCBI GEO repository microarray expression data and clinical/genetic annotations for the following 11 datasets (total n=1981): GSE39582 (n=585), GSE13294 (n=294), GSE14333 (n=157), GSE17536 (n=177), GSE17537 (N=55), GSE20916 (n=81), GSE2109 (n=315), GSE23878 (n=35), GSE33113 (n=90), GSE35896 (n=62) and GSE37892 (n=130). The microarray data were normalized, summarized and log2 transformed using robust multiarray analysis (rma) and batch effects (both between and, where present, within dataset) were removed using Combat. After normalization the probe sets were annotated using the hgu133plus2.db annotation R package (Carlson, 2017). In the case of multiple probe sets interrogating a specific gene, the probe set with the highest mean intensity was selected as representative for that gene. CMS labels for all datasets were obtained from Guinney *et al.* (Guinney et al., 2015) and CRIS labels were obtained from Isella *et al.* (Isella et al., 2017). A processed gene expression dataset of CRC liver metastases (E-TABM-1112, n=120) was obtained from ArrayExpress.

Mouse Model and Patient Gene Expression Signatures

Mouse model gene expression signatures were generated by quantifying differential gene expression data using the DESeq2 R package (Love et al., 2014). Patient-based CMS signatures were derived from the TCGA dataset where differential expression was determined using the limma R package (Ritchie et al., 2015). Human-to-mouse orthologue mappings were obtained from Biomart (http://biomart.org) (Smedley et al., 2015) using the interface provided by the biomaRt R package (Durinck et al., 2009). In case of one-to-many human-to-mouse mappings the mapping with the highest homology percentage was selected. For the mouse model-patient correlation analysis 75 genes were selected that were the up-regulated most significantly (> 0.75 log fold change) in each of the four CMSs (300 genes total) and five CRISs (375 genes total) and calculated the Pearson correlation coefficients of the mouse model signatures with the patient CMS and CRIS signatures.

Infiltration Scores

Infiltration scores were calculated with the MCPcounter R package (Becht et al., 2016a, 2016b) and then standardised per cell type.

KPN VS. APN Signature Survival Analysis

KPN (n=9) vs. APN (n=3) tumor and organoid KPN (n=3) and APN (n=4) gene expression signatures were determined using the same procedure described in the previous paragraph. The 500 most significantly regulated genes were selected to construct the KPN vs. APN signature. No formal p value cut-off was used but all Benjamini-Hochberg adjusted p values were below $1x10^{-6}$. TCGA and NCBI GEO expression datasets (excluding samples of tumor stage IV, without recurrence data or those without a CMS label n=1018) were combined into a single dataset correcting batch effect using ComBat. Expression values were mean-centred gene-wise and individual samples were scored by calculating the Pearson correlation coefficient with the KPN vs. APN signatures. Samples with a correlation score > 0.1 were assigned to the positive group, those with a correlation score < -0.1 to the negative group. Kaplan-Meier plots were generated using the survival R package and the survival distributions were compared with the log-rank test.

GSEA

Geneset enrichment analyses (GSEA) were run using the GSA R package with sample permutation (10,000 permutations) and gene standardization using all genes in the expression dataset, taking the unadjusted p values as output. The input genesets are listed in Table S5. The two TGF-β genesets used to asses TGF-β signaling activity in organoids where obtained from (Plasari et al., 2009; Calon et al., 2012).

NOTCH-score

The NOTCH-score was calculated by summing the standardized expression of a panel of NOTCH related genes (Kwon et al., 2016): *JAG1, JAG2, DLL1, DLL3, DLL4, NOTCH1, NOTCH2, NOTCH3, NOTCH4, HES1, HES2, HEY1, HEY2* and *DTX1*. For the recurrence-free survival analysis of CRC patients from the TCGA dataset, patients were stratified using the within-group median NOTCH-score. *KRAS* mutants were called when mutations encoding amino acids G12, G13 and A143 were present. Kaplan-Meier plots were generated using the survival R package and the survival distributions were compared with the log-rank test.

WNT-score

The WNT-score was calculated by summing the standardised expression of a panel of WNT related genes: *ASCL2, AXIN2, BMP4, MRTO4, HILPDA, NOP16, KITLG, LGR5, MYC, NOP2, PPIF, SOX4, PAAF1, ZIC2 & ZNRF3*.

KPN/KP-score

A KPN (n=3) vs. KP (n=3) organoid gene signature was generated by quantifying differential gene expression data using the DESeq2 R package. We selected the 100 most significantly up-regulated genes (log fold change > 1). No formal p value cut-off was used but all Benjamini-Hochberg adjusted p values were below $1x10^{-2}$. The KPN/KP-score was calculated by summing the standardised expression of the genes in the KPN vs. KP score (Table S7).

Human Serrated Signature

The human serrated signature was derived by comparing WNT target gene expression (Van der Flier et al., 2007) in serrated and tubular adenomas (GSE45270 (n=13) and GSE79460 (n=16)) (Fessler et al., 2016) using the limma R package. The microarray data were normalized summarized and log2 transformed using robust multi array analysis (rma) and batch effects were removed using Combat. After normalization the probe sets were annotated using the hgu133plus2.db annotation R package. In case of multiple probe sets interrogating a specific gene, the probe set with the highest mean intensity was selected as representative for that gene. The mouse model signatures were generated using the DESeq2 R package (Love et al., 2014).

Whole Genome Sequencing

Organoid (tumor derived) and tail DNA were extracted using DNAeasy kit (Qiagen, 69504) as per the manufacturer's instructions. DNA concentration and quality were determined by Nanodrop spectrophotometry and by PicoGreen (Invitrogen, P11496). Whole genome sequencing was performed using 151bp paired-end sequencing on the Illumina HiSeq X platform. Short insert libraries were constructed using prepared flow cells, and clusters generated using standard methods. Samples were sequenced at an average depth of 39x with a minimum coverage of 26x. Data were mapped to the mouse reference genome (GRCm38) using the bwa-mem alignment tool (Li and Durbin, 2009). All whole genome sequencing data have been deposited in the European Nucleotide Archive (ENA) under ENA accession ID: ERP040713.

Somatic Mutation Detection

Somatic variants were detected using CaVEMan, an expectation maximization–based somatic substitution detection algorithm (Jones et al., 2016). Candidate somatic variants were then filtered for quality and to remove known mouse genome variations (Keane et al., 2011). Single point mutations overlapping known structural variants in any of the mouse genomes were also removed due to high misalignment rates in these regions. Small insertion and deletion (indel) detection was performed using the cgp-pindel pipeline (v0.2.4w) (Ye et al., 2009). Detected indels were then filtered for quality, sequence coverage in both tumor and normal, strand bias and for overlap with known simple repeats or indels in the in-house normal panel. Selected mutations were confirmed by Sanger sequencing.

Copy Number Detection

Tumor specific copy number changes were reported using Control free software (Boeva et al., 2012).

DATA AND CODE AVAILABILITY

The datasets generated during this study are available at the European Nucleotide Archive: ERP040713 and ArrayExpress: E-MTAB-6363.

The codes supporting the current study have not been deposited in a public repository because of dependencies on in-house software and data infrastructure, but are available from the corresponding author on request.

QUANTIFICATION AND STATISTICAL ANALYSIS

Statistical analyses were performed using GraphPad Prism software (v7.03 GraphPad software, La Jolla, CA, USA) and R (version 3.4.3) performing tests as indicated and were considered statistically significant, with * p<0.05, ** p<0.01, *** p<0.001, **** p<0.001.

Cancer Cell, Volume *36*

Supplemental Information

Epithelial NOTCH Signaling Rewires the Tumor Microenvironment of Colorectal Cancer to Drive Poor-Prognosis Subtypes and Metastasis

Rene Jackstadt, Sander R. van Hooff, Joshua D. Leach, Xabier Cortes-Lavaud, Jeroen O. Lohuis, Rachel A. Ridgway, Valérie M. Wouters, Jatin Roper, Timothy J. Kendall, Campbell S. Roxburgh, Paul G. Horgan, Colin Nixon, Craig Nourse, Matthias Gunzer, William Clark, Ann Hedley, Omer H. Yilmaz, Mamunur Rashid, Peter Bailey, Andrew V. Biankin, Andrew D. Campbell, David J. Adams, Simon T. Barry, Colin W. Steele, Jan Paul Medema, and Owen J. Sansom

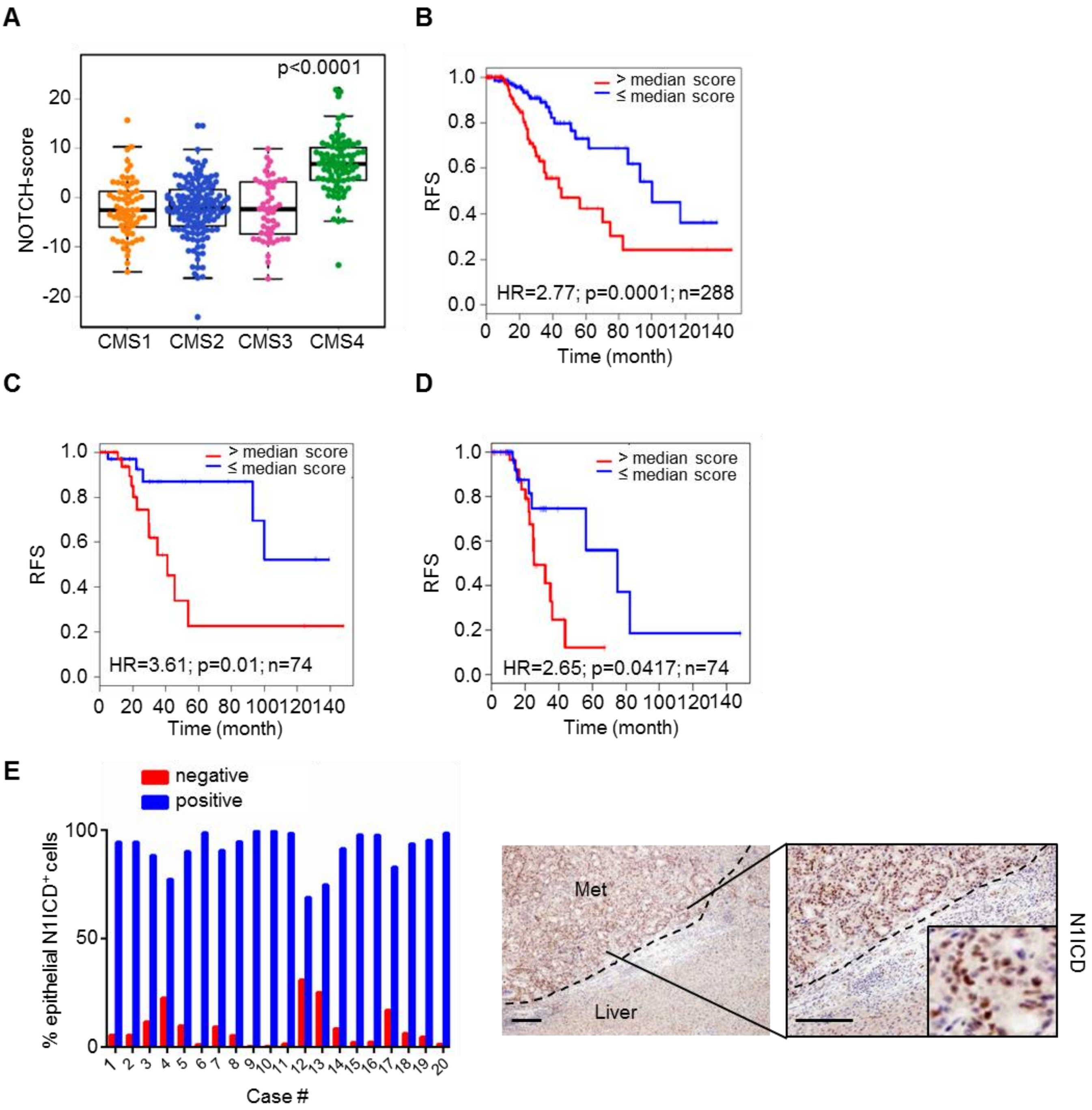

Figure S1 related to Figure 1. (A) NOTCH-score in TCGA CRC tumors separated by CMS. p value was calculated using a two sample two-tailed Welch t-test, comparing CMS4 to CMS1/2/3. The boxes indicate interquartile range (IQR), horizontal black lines median. The whiskers range to a maximum of 1.5 times the IQR. Data points outside 1.5 IQR are represented by individual dots. **(B)** Recurrence free survival (RFS) of CRC patients (TCGA), stratified using the NOTCH-score, all CMS stratified patients; blue line shows expression ≤ median score, red line shows expression > median score. **(C)** RFS of CRC patients (TCGA), stratified using the NOTCH-score, of all four CMSs in patients with *KRAS* mutations; blue line shows expression ≤ median score, red line shows expression > median score. **(D)** RFS of CRC patients (TCGA), stratified using the NOTCH-score, CMS4 stratified patients; blue line shows expression ≤ median score, red line shows expression > median score. **(E)** Left panel: Quantification of percentage positive or negative nuclear N1ICD expression (IHC) in human CRC liver metastases. Right panel: representative images of N1ICD expression (IHC) in human CRC liver metastases; scale bars, 200 µm.

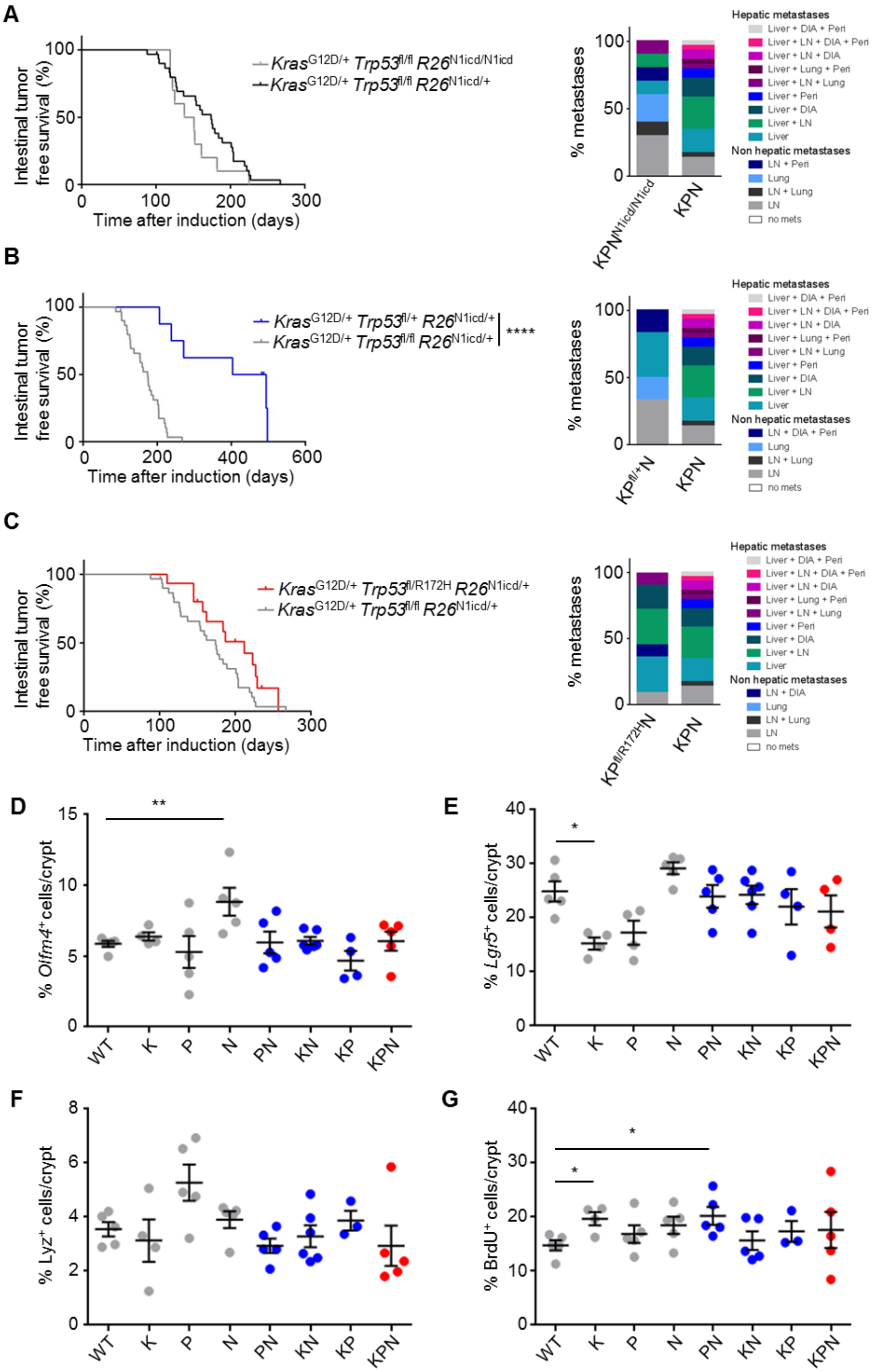

Figure S2 related to Figure 1. (A) Left panel: Kaplan-Meier survival curves of intestinal tumor free survival of KPN mice or KPN mice homozygote for *Rosa26*^{N1icd}. Right panel: Percent incidence of macroscopic metastases per genotype of *villin*Cre^{ER} *Kras*$^{G12D/+}$ *Trp53*$^{fl/fl}$ *R26*$^{N1icd/N1icd}$ (KPN$^{N1icd/N1icd}$) n=10, *villin*Cre^{ER} *Kras*$^{G12D/+}$ *Trp53*$^{fl/fl}$ *R26*$^{N1icd/+}$ (KPN) n=29 mice. **(B)** Left panel: Kaplan-Meier survival curves of intestinal tumor free survival of KPN mice or KPN mice heterozygote for floxed *Trp53*, analyzed by log-rank (Mantel–Cox) test. Right panel: Percent incidence of macroscopic metastases per genotype of *villin*Cre^{ER} *Kras*$^{G12D/+}$ *Trp53*$^{fl/+}$ *R26*$^{N1icd/+}$ (KP$^{fl/+}$N) n=6, *villin*Cre^{ER} *Kras*$^{G12D/+}$ *Trp53*$^{fl/fl}$ *R26*$^{N1icd/+}$ (KPN) n=29 mice. **(C)** Left panel: Kaplan-Meier survival curves of intestinal tumor free survival of KPN mice or KPN mice with *Trp53*$^{fl/R172H}$. Right panel: Percent incidence of macroscopic metastases per genotype of *villin*Cre^{ER} *Kras*$^{G12D/+}$ *Trp53*$^{fl/R172H}$ *R26*$^{N1icd/+}$ (KP$^{fl/R172H}$N) n=12, *villin*Cre^{ER} *Kras*$^{G12D/+}$ *Trp53*$^{fl/fl}$ *R26*$^{N1icd/+}$ (KPN) n=29 mice. In (A-C) DIA = diaphragm, LN = Lymph-node, Peri = peritoneal carcinomatosis. Quantification of percentage of positive cells per crypt stained for **(D)** *Olfm4*, **(E)** *Lgr5*, **(F)** Lysozyme (Lyz) and **(G)** BrdU. WT = *villin*Cre^{ER}; K = *villin*Cre^{ER} *Kras*$^{G12D/+}$; P = *villin*Cre^{ER} *Trp53*$^{fl/fl}$; N = *villin*Cre^{ER} *Rosa26*$^{N1icd/+}$; PN = *villin*Cre^{ER} *Trp53*$^{fl/fl}$ *Rosa26*$^{N1icd/+}$; KN = *villin*Cre^{ER} *Kras*$^{G12D/+}$ *Rosa26*$^{N1icd/+}$; KP = *villin*Cre^{ER} *Kras*$^{G12D/+}$ *Trp53*$^{fl/fl}$; KPN = *villin*Cre^{ER} *Kras*$^{G12D/+}$ *Trp53*$^{fl/fl}$ *Rosa26*$^{N1icd/+}$. Error bars represent mean ± SEM, analyzed by Mann–Whitney U test, two-tailed.

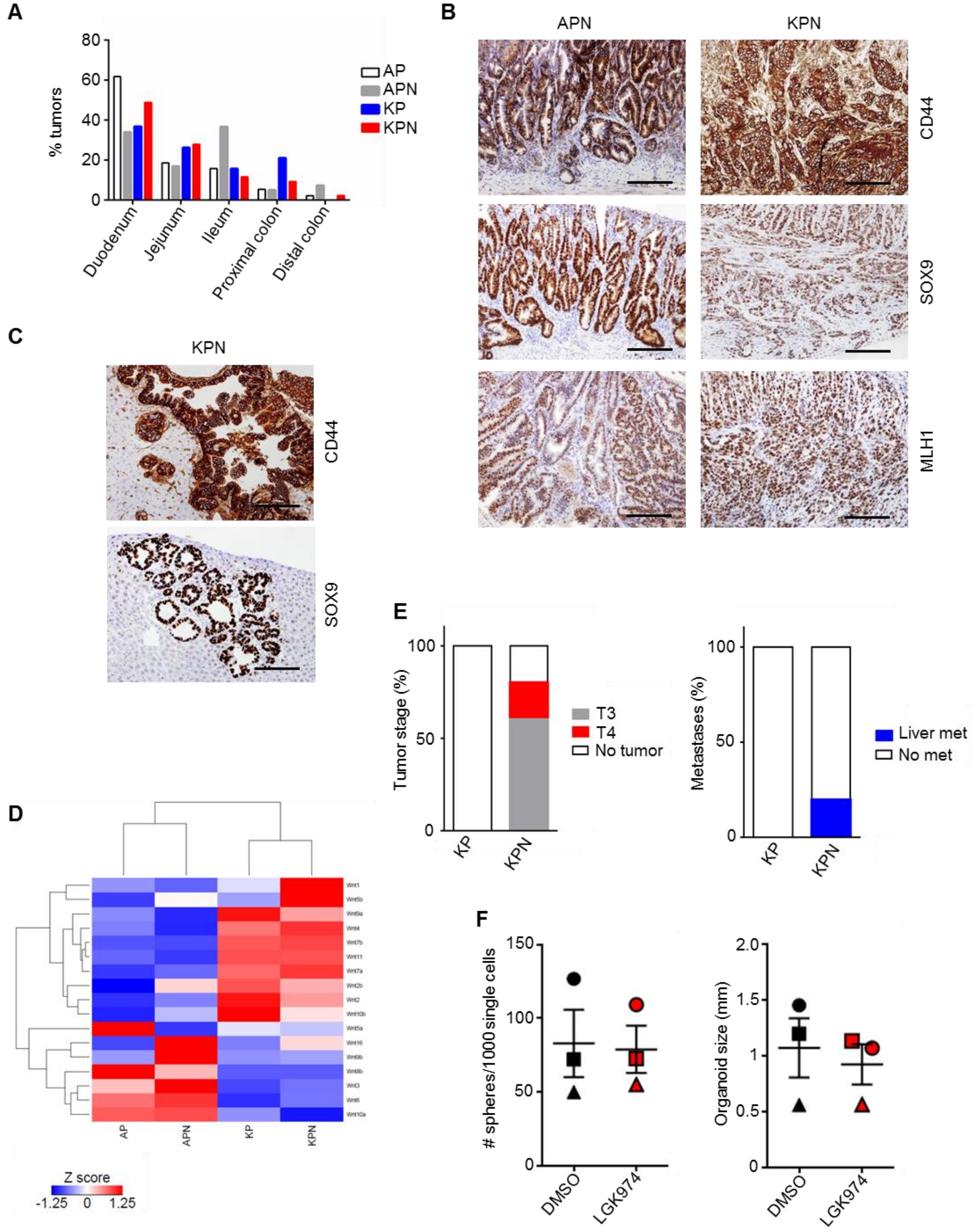

Figure S3 related to Figure 2, 3 and 4. (A) Distribution of primary tumors in the intestines of mice with indicated genotypes. AP n=10; APN n=12; KP n=14; KPN n=28. **(B)** Representative IHC for the indicated markers on primary tumors from APN and KPN mice at endpoint; scale bars, 100 µm. **(C)** Representative IHC for the indicated markers on liver metastases of KPN mice at endpoint; scale bars, 100 µm. **(D)** Heat-map showing WNT ligand expression in primary tumors of intestinal GEMMs. **(E)** Quantification of primary tumor stage and percentage of mice with metastasis at day 85 after tamoxifen administration; KP n=5; KPN n=5. **(F)** Average KP organoid size or number seven days post single cell seeding under indicated conditions; LGK974 10 µM. Each sample was generated from a separate tumor n=3; error bars represent mean ± SEM.

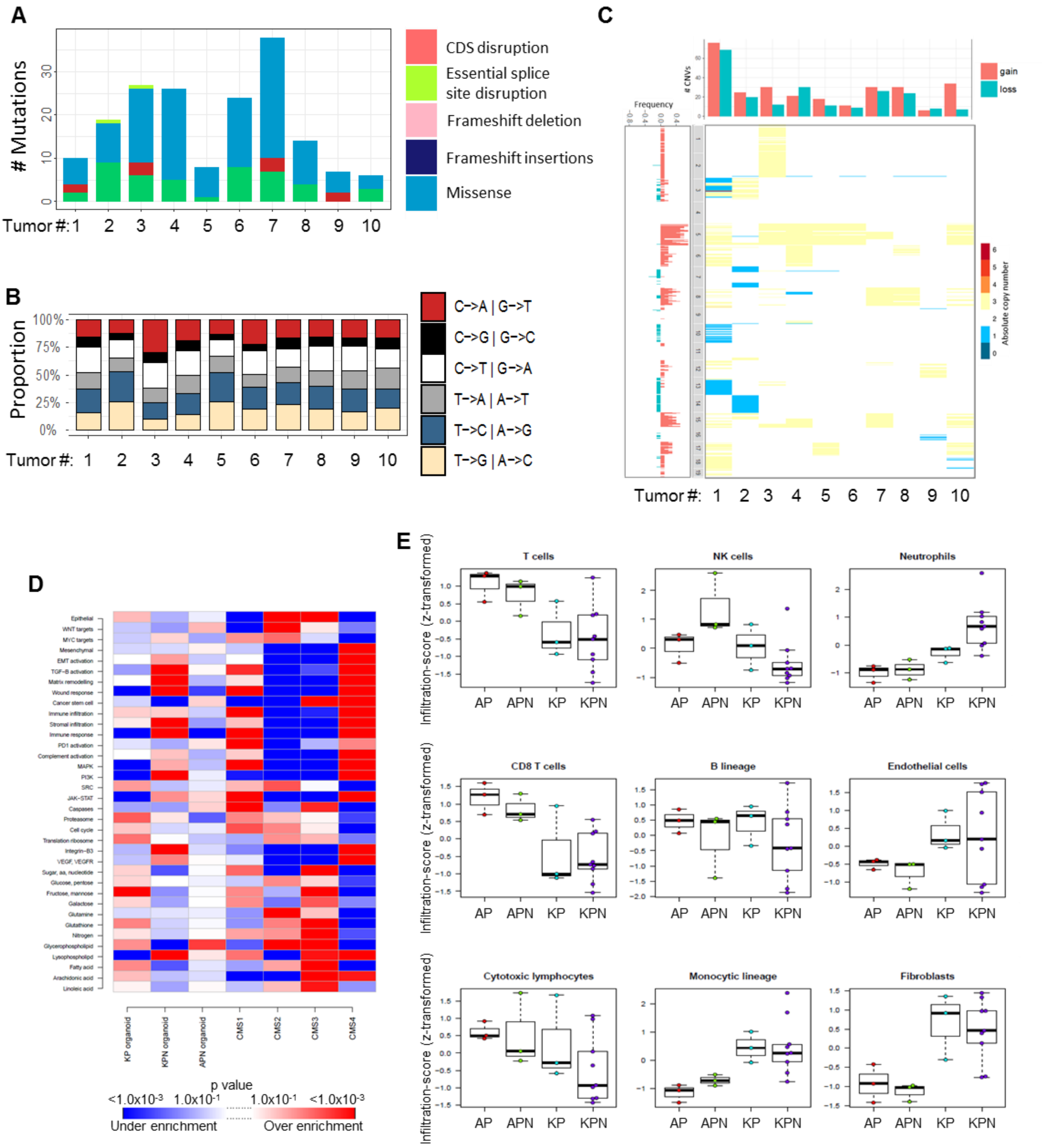

Figure S4 related to Figure 3 and 4. (A) Landscape of somatic mutations in KPN tumor derived organoids; number of somatic point mutations per sample. **(B)** Nucleotide changes per sample. **(C)** Landscape of somatic copy number changes (bottom right panel). Number of gains and losses per sample (top panel). Absolute copy number profile (bottom left panel). Gain and loss frequency across different chromosomes. **(D)** GSEA results for organoids with indicated genotypes and CMS1-4 CRC patient tumors. Replicates: APN (organoids) n=4; KP (organoids) n=3; KPN (organoids) n=3. **(E)** Box plots showing standardized infiltration scores (calculated with MCPcounter) in primary tumor derived expression profiles for indicated genotypes. The boxes indicate interquartile range (IQR), horizontal black lines median. The whiskers range to a maximum of 1.5 times the IQR. Data points outside 1.5 IQR are represented by individual dots.

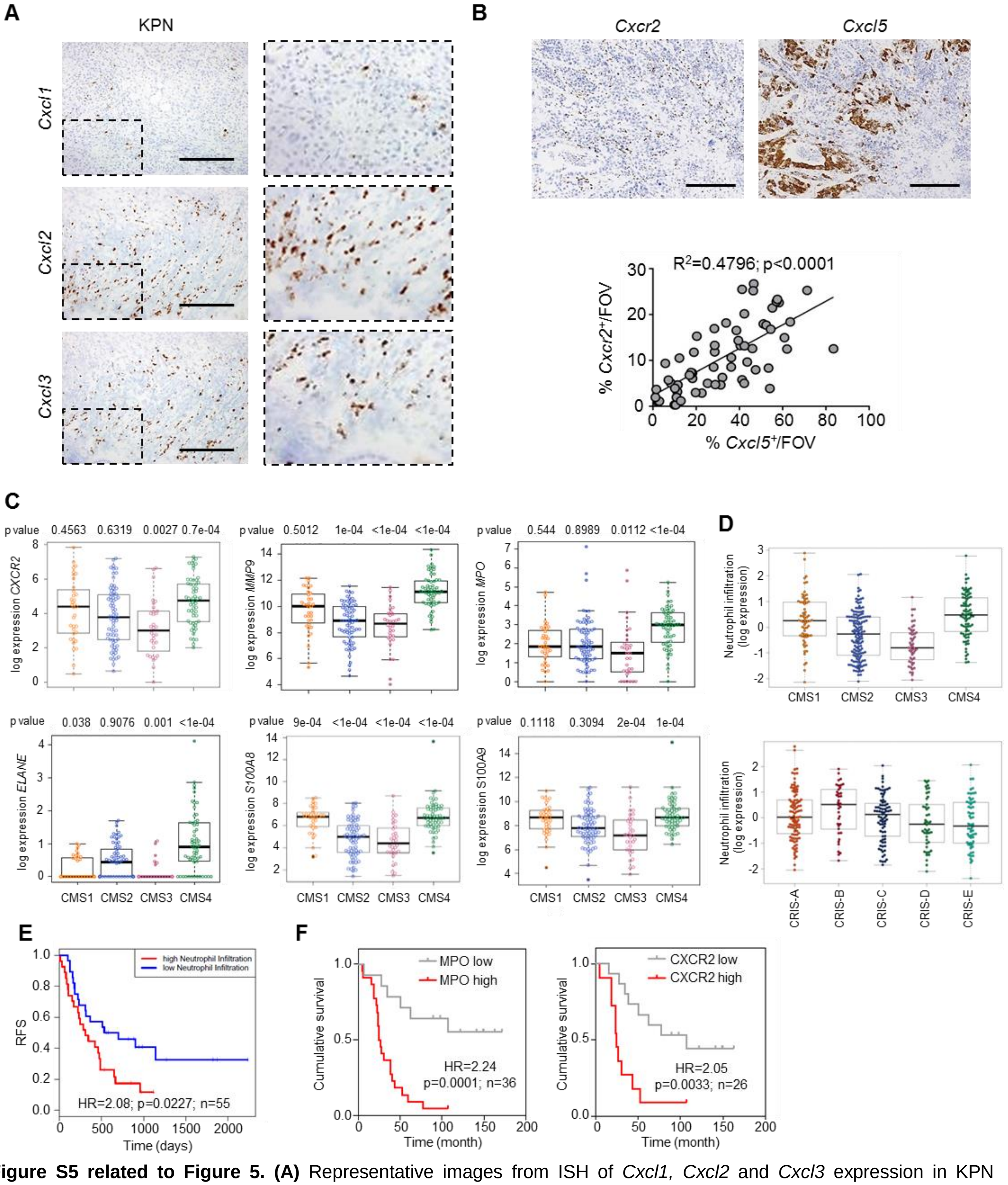

Figure S5 related to Figure 5. (A) Representative images from ISH of *Cxcl1, Cxcl2* and *Cxcl3* expression in KPN primary tumors; scale bars, 100 µm. **(B)** Representative image (top) and expression correlation analysis (bottom) of *Cxcl5* and *Cxcr2* expression (ISH) in KPN primary intestinal tumors per field of view (n=2 KPN primary tumors; 30 FOVs per tumor were analyzed); p value by Pearson correlation; scale bars, 100 µm. **(C)** Expression analysis of neutrophil markers encoding genes as indicated in CRC patient tumors (TCGA) per CMS. P values were calculated using the ANOVA-test. **(D)** Top: Neutrophil infiltration in CRC patient tumors (TCGA) per CMS; $p=3x10^{-8}$, CMS4 vs. others. Bottom: Expression analysis of Neutrophil infiltration in CRC patient tumors (TCGA) per CRIS; p=0.01, CRIS-B vs. others. P values were calculated using the ANOVA-test. **(E)** Recurrence free survival of patients with treatment naïve CRC metastases (E-TABM-1112), stratified using the neutrophil infiltration-score. Blue line shows expression ≤ median score (low), red line shows expression > median score (high). **(F)** Cumulative survival of patients (Glasgow-cohort), stratified using IHC for MPO or CXCR2 in metastases post synchronous resection of colorectal primary tumors and liver metastases. P values were calculated by log-rank (Mantel–Cox) test. The boxes in C and D indicate interquartile range (IQR), horizontal black lines median. The whiskers range to a maximum of 1.5 times the IQR. Data points outside 1.5 IQR are represented by individual dots.

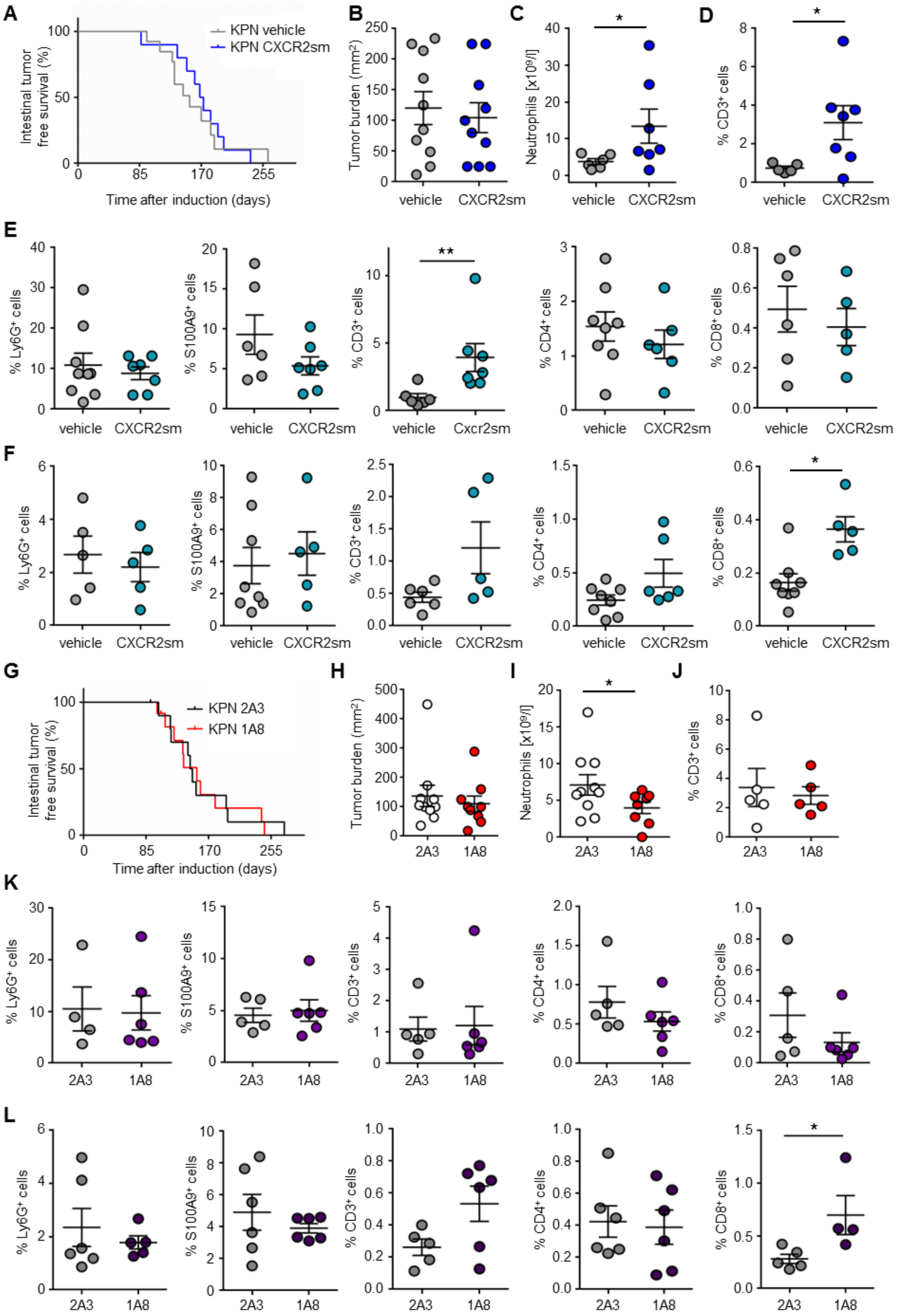

Figure S6 related to Figure 5. (A) Kaplan-Meier survival curves for intestinal tumor free survival of KPN mice treated with vehicle (n=13) or CXCR2 small molecule (CXCR2sm) (n=10), treatments started on day 85 after tamoxifen administration. **(B)** Tumor burden quantified in vehicle (n=10) and CXCR2sm (n=10) treated KPN mice at endpoint. **(C)** Blood neutrophil counts at endpoint for vehicle (n=6) and CXCR2sm (n=7) treated mice; error bars represent mean ± SEM, analyzed by Mann–Whitney U test, one-tailed. **(D)** Quantification of IHC for CD3 positive cells per KPN liver after one week treatment (n≥5). Error bars represent mean ± SEM, analyzed by Mann–Whitney U test, two-tailed. **(E)** Quantification of IHC analysis of indicated markers per primary tumor in KPN mice treated with vehicle or CXCR2sm from day 85 after tamoxifen administration (n≥5). Error bars represent mean ± SEM, analyzed by Mann–Whitney U test, two-tailed. **(F)** Quantification of IHC analysis of indicated markers per liver of KPN mice treated with vehicle or CXCR2sm from day 85 after tamoxifen administration (n≥5). Error bars represent mean ± SEM, analyzed by Mann–Whitney U test, two-tailed. **(G)** Kaplan-Meier survival curves for intestinal tumor free survival of KPN mice treated with isotype control (2A3) (n=11) or Ly6G blocking antibody (1A8) (n=12). Treatment started 85 days after tamoxifen administration. **(H)** Tumor burden of 2A3 (n=10) or 1A8 (n=9) treated KPN mice at endpoint. **(I)** Blood neutrophil count at endpoint for indicated treatments (2A3 n=10, 1A8 n=8). Error bars represent mean ± SEM., analyzed by Mann–Whitney U test, one-tailed. **(J)** Quantification of IHC for CD3 positive cells per KPN liver after one week treatment (n=5). Error bars represent mean ± SEM, analyzed by Mann–Whitney U test, two-tailed. **(K)** Quantification of IHC analysis of indicated markers per primary tumor of KPN mice treated with 2A3 or 1A8 from day 85 post tamoxifen administration (n≥4). Error bars represent mean ± SEM. **(L)** Quantification of IHC analysis of indicated markers per liver of KPN mice treated with 2A3 or 1A8 from day 85 after tamoxifen administration (n≥4). Error bars represent mean ± SEM, analyzed by Mann–Whitney U test, two-tailed.

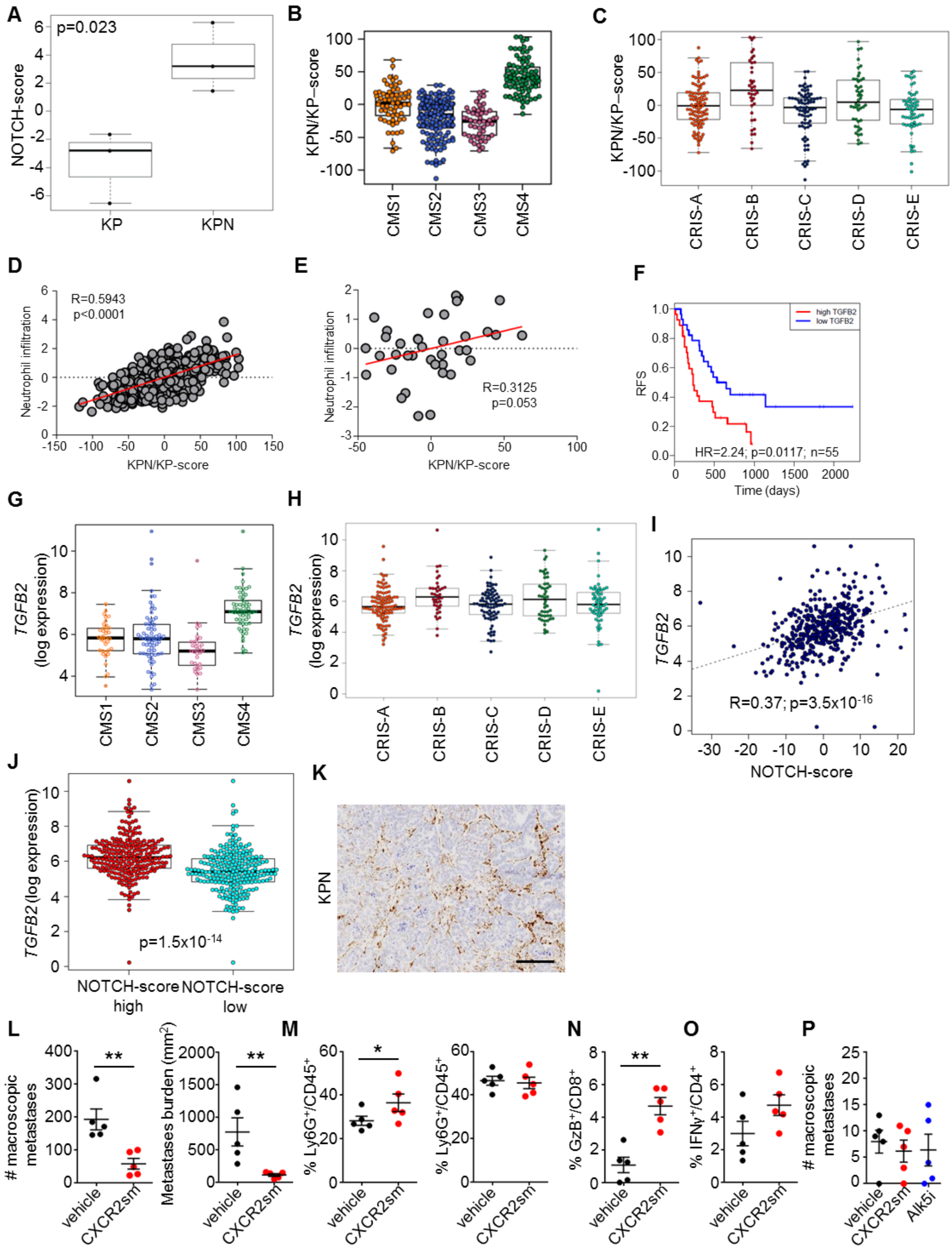

Figure S7 related to Figure 6 and 7. (A) NOTCH-score expression in KP (n=3) and KPN (n=3) organoids. P value was calculated with a t-test. **(B)** KPN/KP-score in CRC patient tumors (TCGA) per CMS. P value was calculated using the ANOVA-test; p=5.31x10^{-33}, CMS4 vs. others. **(C)** Expression analysis of KPN/KP-score in CRC patient tumors (TCGA) per CRIS. P value was calculated using the ANOVA-test; p=0.00027, CRIS-B vs. others. **(D)** Correlation of the KPN/KP-score and neutrophil infiltration in human CRC patient tumors (TCGA). P value was calculated by Pearson correlation. **(E)** Correlation of the KPN/KP-score and neutrophil infiltration in human serrated adenoma. P value was calculated by Pearson correlation. **(F)** Recurrence free survival of patients with treatment naïve CRC metastases (E-TABM-1112), stratified using *TGFB2* expression. Blue line shows expression ≤ median score (low), red line shows expression > median score (high). **(G)** Expression analysis of *TGFB2* in CRC patient tumors (TCGA) per CMS. P value was calculated using the ANOVA-test; p=1x10^{-4}, CMS4 vs. others. **(H)** Expression analysis of *TGFB2* in CRC patient tumors (TCGA) per CRIS. P value was calculated using the ANOVA-test; p=0.01, CRIS-B vs. others. **(I)** Correlation of *TGFB2* expression and the NOTCH-score in CRC patient tumors (TCGA). P value was calculated by Pearson correlation. **(J)** Expression analysis of *TGFB2* in CRC patient tumors (TCGA) with high or low NOTCH-score. P value was calculated with a t-test. **(K)** Representative ISH analysis of *Tgfb1* expression in KPN primary tumor; scale bar, 100 µm. **(L)** Number and burden of macroscopic liver metastases four weeks post intra splenic transplantation of KPN organoids (vehicle n=5, CXCR2sm n=5). **(M)** Quantification of flow cytometry analysis for neutrophils in blood (left) or liver metastases (right) four weeks post transplantation (vehicle n=5, CXCR2sm n=5), error bars represent mean ± SEM, analyzed by Mann–Whitney U test, one-tailed **(N)** Quantification of flow cytometry analysis for T cell subsets (% Granzyme B (GzB)$^+$/CD8$^+$) in liver metastases four weeks post transplantation (vehicle n=5, CXCR2sm n=5). **(O)** Quantification of flow cytometry analysis for T cell subsets (% Interferon-γ (IFN-γ)$^+$/CD4$^+$) in liver metastases four weeks post transplantation (vehicle n=5, CXCR2sm n=5). **(P)** Number of macroscopic liver metastases four weeks post intra splenic transplantation of KPN organoids in immune-deficient nude mice (vehicle n=5, CXCR2sm n=5, Alk5i n=5). In S7L and S7N error bars represent mean ± SEM, analyzed by Mann–Whitney U test, two-tailed. The boxes in A, B, C, G, H and J indicate interquartile range (IQR), horizontal black lines median. The whiskers range to a maximum of 1.5 times the IQR. Data points outside 1.5 IQR are represented by individual dots.

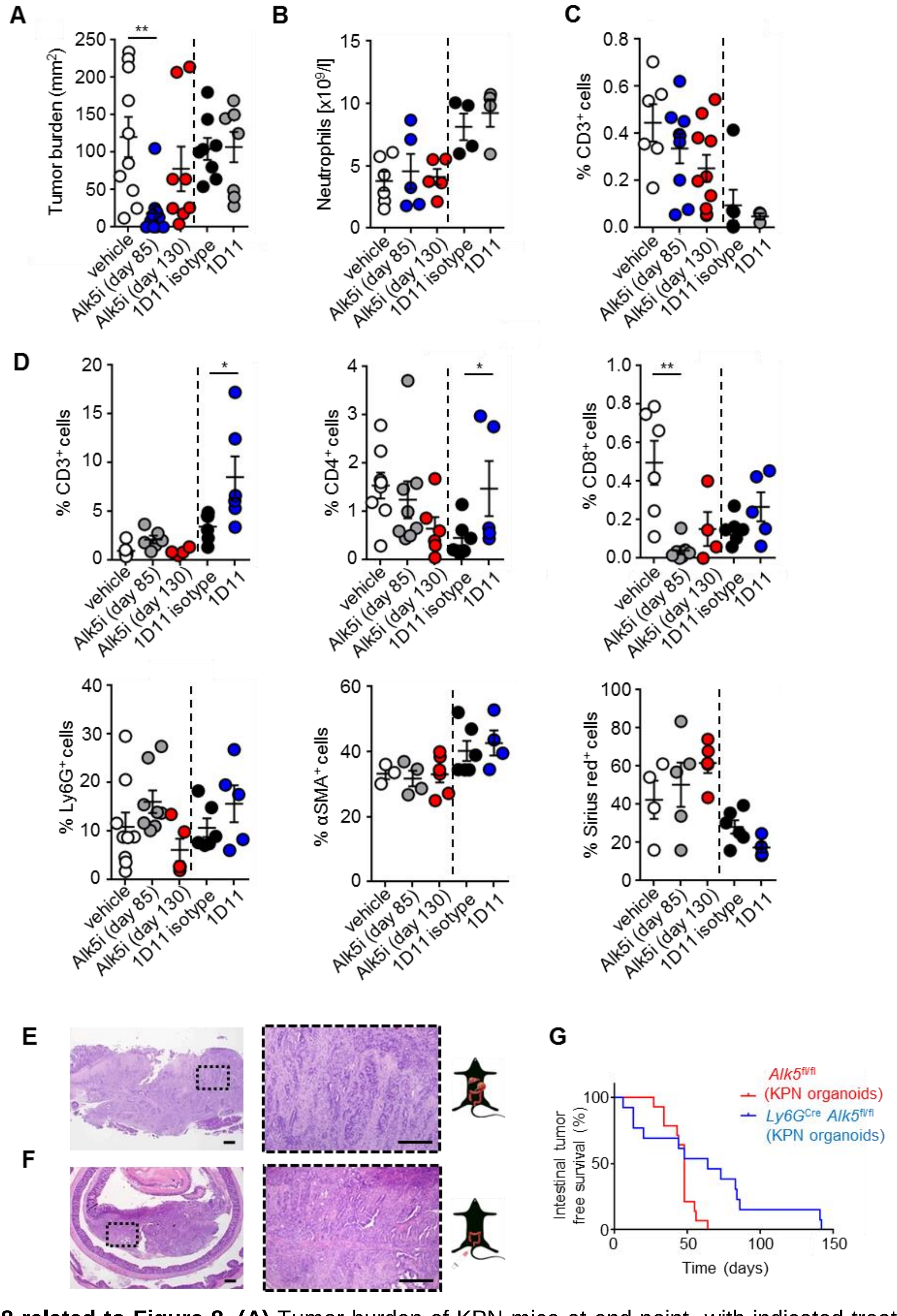

Figure S8 related to Figure 8. (A) Tumor burden of KPN mice at end point with indicated treatments (vehicle from day 85, Alk5i from day 85 or day 130, 1D11 or 1D11 isotype control from day 85 after tamoxifen administration), error bars represent mean ± SEM, analyzed by Mann–Whitney U test, two-tailed. **(B)** Blood neutrophil counts at endpoint of KPN mice treated as indicated in A; error bars represent mean ± SEM. **(C)** Quantification of IHC for CD3 positive cells per KPN liver at endpoint (n≥3), treated as indicated in A. Error bars represent mean ± SEM. **(D)** Quantification of IHC for Ly6G, CD3, CD4, CD8, αSMA and Sirius red positive cells per KPN primary tumor at endpoint after treatment as indicated in A; error bars represent mean ± SEM, analyzed by Mann–Whitney U test, two-tailed. **(E)** Representative H&E images of a KPN GEMM colonic primary tumor at end point; scale bars, 100 µm. **(F)** Representative H&E images of intra colonic transplanted KPN organoids at end point; scale bars, 100 µm. **(G)** Kaplan-Meier survival curves of intestinal tumor free survival from mice with indicated genotypes (*Alk5*$^{fl/fl}$ n=14; *Ly6G*^{Cre} *Alk5*$^{fl/fl}$ n=13) and KPN organoid transplantation.
